# A multifaceted intervention to improve diagnosis and early management of hospitalised patients with suspected acute brain infections in Brazil, India, and Malawi: an international multicentre intervention study

**DOI:** 10.1016/S0140-6736(25)00263-6

**Published:** 2025-03-10

**Authors:** Bhagteshwar Singh, Gareth D Lipunga, Premkumar Thangavelu, Shalley Dhar, Lorena Ferreira Cronemberger, Kundavaram Paul Prabhakar Abhilash, Asha Mary Abraham, Carlos Alexandre Antunes de Brito, Maria Lúcia Brito Ferreira, Nagarathna Chandrashekar, Rui Duarte, Anna Fajardo Modol, Ben Chirag Ghale, Gagandeep Kang, Vykuntaraju K Gowda, Kevin Kuriakose, Suzannah Lant, Macpherson Mallewa, Emmie Mbale, Shona C Moore, Gloria Mwangalika, Prasanna B T Kamath, Patricia Navvuga, Alinane Linda Nyondo-Mipando, Tamara J Phiri, Camila Pimentel Lopes de Melo, B S Pradeep, Rebecca Rawlinson, Irene Sheha, Priya Treesa Thomas, Charles R Newton, Patricia Carvalho de Sequeira, James J Sejvar, Tarun Dua, Lance Turtle, Valsan Philip Verghese, Luciano Wagner de Melo Santiago Arraes, Nicola Desmond, Ava Easton, Jessica Anne Jones, Richard J Lilford, M Netravathi, Fiona McGill, Benedict D Michael, Victor Mwapasa, Michael J Griffiths, Christopher M Parry, Vasanthapuram Ravi, Girvan Burnside, Brain Infections Global Intervention Study Group, Ajith Sivadasan, Reginald G Alex, Alex Shabani, Aline de Moura Brasil Matos, Anandhi Arumugam, Anbu Suresh Rao, Andre Silva Lira de Lucena, Angel Miraclin, Anitha Aswathanarayan, Anna Rosala-Hallas, Anna Simon, Anushri Somasundaran, Aparna Vasudev, Archana G E, Arnold E Kapachika, Arvind Natarajan, Audrin Lenin, Balaji Veeraraghavan, Blessings Kadzuwa, Brigitte Denis, Catherine Anscombe, Chimwemwe Maluwa, Chishala Chafunya, Chitra Pattabiraman, Clifford Chitala, Daisy Sampreetha, Debasis Das Adhikari, Dhanalakshmi, Divya Deodhar, Divya Mathew, Durjoy Lahiri, Elizabeth Rodgers, Eva Maria Hodel, Evelyn López, Eveness Chiipanthenga, Felix Jamu, G V Basavaraja, Gina Chandy, Gnanadurai John Fletcher, Gopalkrishna Gururaj, Greta Wood, Gurrapu Rakesh, Hannah Persis Jeyakumar, J Vignesh Kumar, Jagadeesh Munichannappa, Jagan S, Jailson B Correia, James Tovey, Janet Harrison, Jenala Njirammadzi, Jenevi Margaret Mendosa, John Jude Anthony Prakash, Jones Kadewere, Jyoti Sharma, Karen Lobo, Karthik Gunasekaran, Kasi Sekar, Kaustubh Somalwar, Keshav Murthy, Lakshmi, Liam Whittle, Lucia Jansi Rani S, Madalitso Kalima, Madhu Sudan, Mahantesh, Mandara Ganganakudige Manjappaiah, Maria Ellen da Silva Antonio, Matthew Smyth, Mavis Menyere, Memory Siwombo, Monica Kamwana, Morganna Costa Lima, SR Muniraju, Nalini Newbigging, Nathalie van den Brekel, C Navya, Nihal Thomas, Philip Sajiwa, K Prabhakar, Prasannakumar Palanikumar, Priscilla Salley, Priyalatha SK, Rachael Brookes, Radhika, Ritika Thakur, Rituwij Kumar, Samuel George Hansdak, Sanjith Aaron, Santosh Chaturvedi, Santhoshkumar Rajendran, Sathish Kumar, Sathya Prabhu, Shoba Mammen, Sithembile Bilima, Sithembinkosi Mhlanga, Sneha Deena Varkki, Sofia R Valdoleiros, Sri Hari Alapati, Sreenath S, Stephen Ray, Sudha Reddy V R, Thaise Yasmine Vasconcelos de Lima Cavalcanti, Tina Damodar, Trudie Lang, Uddhav Kinhal, Vasundharaa S Nair, Vijaykumar SN, Vikram Holla, Jennifer Cornick, Rafael Freitas de Oliveira França, Anita S Desai, Priscilla Rupali, Tom Solomon

**Affiliations:** Institute of Infection, Veterinary, and Ecological Sciences, University of Liverpool, Liverpool, UK; Tropical and Infectious Diseases Unit, Royal Liverpool University Hospital, Liverpool, UK; Christian Medical College, Vellore, Tamil Nadu, India; Liverpool School of Tropical Medicine, Liverpool, UK; Malawi-Liverpool-Wellcome Trust Clinical Research Programme, Blantyre, Malawi; Christian Medical College, Vellore, Tamil Nadu, India; National Institute of Mental Health and Neuro Sciences, Bangalore, Karnataka, India; Department of Sociology, Federal University of Pernambuco, Recife, Brazil; Oswaldo Cruz Foundation Pernambuco, Recife, Brazil; Christian Medical College, Vellore, Tamil Nadu, India; Christian Medical College, Vellore, Tamil Nadu, India; Department of Clinical Medicine, Federal University of Pernambuco, Recife, Brazil; Hospital das Clinicas, Recife, Brazil; Autoimmune Research Institute, Recife, Brazil; Department of Neurology, Hospital da Restauração, Recife, Brazil; National Institute of Mental Health and Neuro Sciences, Bangalore, Karnataka, India; Institute of Population Health, University of Liverpool, Liverpool, UK; Institute of Infection, Veterinary, and Ecological Sciences, University of Liverpool, Liverpool, UK; Christian Medical College, Vellore, Tamil Nadu, India; Christian Medical College, Vellore, Tamil Nadu, India; Department of Neurology, Indira Gandhi Institute of Child Health, Bangalore, Karnataka, India; Institute of Infection, Veterinary, and Ecological Sciences, University of Liverpool, Liverpool, UK; Institute of Infection, Veterinary, and Ecological Sciences, University of Liverpool, Liverpool, UK; National Institute of Health and Care Research Health Protection Research Unit in Emerging and Zoonotic Infections, University of Liverpool, Liverpool, UK; Kamuzu University of Health Sciences, Blantyre, Malawi; Kamuzu University of Health Sciences, Blantyre, Malawi; Queen Elizabeth Central Hospital, Blantyre, Malawi; Institute of Infection, Veterinary, and Ecological Sciences, University of Liverpool, Liverpool, UK; National Institute of Health and Care Research Health Protection Research Unit in Emerging and Zoonotic Infections, University of Liverpool, Liverpool, UK; The Pandemic Institute, Liverpool, UK; Malawi-Liverpool-Wellcome Trust Clinical Research Programme, Blantyre, Malawi; Sri Devaraj Urs Academy of Higher Education and Research, Kolar, India; Sri Devaraj Urs Medical College, Kolar, India; R L Jalappa Hospital, Kolar, India; Institute of Infection, Veterinary, and Ecological Sciences, University of Liverpool, Liverpool, UK; Malawi-Liverpool-Wellcome Trust Clinical Research Programme, Blantyre, Malawi; Kamuzu University of Health Sciences, Blantyre, Malawi; Kamuzu University of Health Sciences, Blantyre, Malawi; Queen Elizabeth Central Hospital, Blantyre, Malawi; Oswaldo Cruz Foundation Pernambuco, Recife, Brazil; National Institute of Mental Health and Neuro Sciences, Bangalore, Karnataka, India; Institute of Infection, Veterinary, and Ecological Sciences, University of Liverpool, Liverpool, UK; National Institute of Health and Care Research Health Protection Research Unit in Emerging and Zoonotic Infections, University of Liverpool, Liverpool, UK; Malawi-Liverpool-Wellcome Trust Clinical Research Programme, Blantyre, Malawi; National Institute of Mental Health and Neuro Sciences, Bangalore, Karnataka, India; Department of Psychiatry, University of Oxford, Oxford, UK; KEMRI-Wellcome Trust Research Programme, Kilifi, Kenya; Laboratory of Arboviruses and Haemorrhagic Viruses, Oswaldo Cruz Institute, Oswaldo Cruz Foundation, Rio de Janeiro, Brazil; Division of High-Consequence Pathogens and Pathology, National Center for Emerging and Zoonotic Infectious Diseases, US Centers for Disease Control and Prevention, Atlanta, GA, USA; Brain Health Unit, World Health Organization, Geneva, Switzerland; Institute of Infection, Veterinary, and Ecological Sciences, University of Liverpool, Liverpool, UK; National Institute of Health and Care Research Health Protection Research Unit in Emerging and Zoonotic Infections, University of Liverpool, Liverpool, UK; Tropical and Infectious Diseases Unit, Royal Liverpool University Hospital, Liverpool, UK; Christian Medical College, Vellore, Tamil Nadu, India; Hospital Correia Picanço, Recife, Brazil; Malawi-Liverpool-Wellcome Trust Clinical Research Programme, Blantyre, Malawi; Liverpool School of Tropical Medicine, Liverpool, UK; Institute of Infection, Veterinary, and Ecological Sciences, University of Liverpool, Liverpool, UK; Encephalitis International, Malton, UK; Alder Hey Children’s Hospital, Liverpool, UK; Liverpool School of Tropical Medicine, Liverpool, UK; Glan Clwyd Hospital, Betsi Cadwaladr University Health Board, Bangor, UK; University of Birmingham, Birmingham, UK; National Institute of Mental Health and Neuro Sciences, Bangalore, Karnataka, India; Leeds Teaching Hospitals NHS Trust, Leeds, UK; Institute of Infection, Veterinary, and Ecological Sciences, University of Liverpool, Liverpool, UK; National Institute of Health and Care Research Health Protection Research Unit in Emerging and Zoonotic Infections, University of Liverpool, Liverpool, UK; The Walton Centre NHS Foundation Trust, Liverpool, UK; Kamuzu University of Health Sciences, Blantyre, Malawi; Institute of Infection, Veterinary, and Ecological Sciences, University of Liverpool, Liverpool, UK; National Institute of Health and Care Research Health Protection Research Unit in Emerging and Zoonotic Infections, University of Liverpool, Liverpool, UK; Centre for Child and Adolescent Health Research, University of Sydney, Sydney, NSW, Australia; Liverpool School of Tropical Medicine, Liverpool, UK; National Institute of Mental Health and Neuro Sciences, Bangalore, Karnataka, India; Institute of Population Health, University of Liverpool, Liverpool, UK; Glan Clwyd Hospital, Betsi Cadwaladr University Health Board, Bangor, UK; Institute of Infection, Veterinary, and Ecological Sciences, University of Liverpool, Liverpool, UK; Malawi-Liverpool-Wellcome Trust Clinical Research Programme, Blantyre, Malawi; Oswaldo Cruz Foundation Pernambuco, Recife, Brazil; National Institute of Mental Health and Neuro Sciences, Bangalore, Karnataka, India; Christian Medical College, Vellore, Tamil Nadu, India; Institute of Infection, Veterinary, and Ecological Sciences, University of Liverpool, Liverpool, UK; National Institute of Health and Care Research Health Protection Research Unit in Emerging and Zoonotic Infections, University of Liverpool, Liverpool, UK; The Walton Centre NHS Foundation Trust, Liverpool, UK; The Pandemic Institute, Liverpool, UK

## Abstract

**Background:**

Brain infections pose substantial challenges in diagnosis and management and carry high mortality and morbidity, especially in low-income and middle-income countries. We aimed to improve the diagnosis and early management of patients admitted to hospital (adults aged 16 years and older and children aged >28 days) with suspected acute brain infections at 13 hospitals in Brazil, India, and Malawi.

**Methods:**

With hospital stakeholders, policy makers, and patient and public representatives, we co-designed a multifaceted clinical and laboratory intervention, informed by an evaluation of routine practice. The intervention, tailored for each setting, included a diagnostic and management algorithm, a lumbar puncture pack, a testing panel, and staff training. We used multivariable logistic regression and interrupted time series analysis to compare the coprimary outcomes—the percentage of patients achieving a syndromic diagnosis and the percentage achieving a microbiological diagnosis before and after the intervention. The study was registered at ClinicalTrials.gov (NCT04190303) and is complete.

**Findings:**

Between Jan 5, 2021, and Nov 30, 2022, we screened 10 462 patients and enrolled a total of 2233 patients at 13 hospital sites connected to the four study centres in Brazil, India, and Malawi. 1376 (62%) were recruited before the intervention and 857 (38%) were recruited after the intervention. 2154 patients (96%) had assessment of the primary outcome (1330 [62%] patients recruited pre-intervention and 824 [38%] recruited post-intervention). The median age across centres was 23 years (IQR 6–44), with 1276 (59%) being adults aged 16 years or older and 888 (41%) children aged between 29 days and 15 years; 1264 (59%) patients were male and 890 (41%) were female. Data on race and ethnicity were not recorded. 1020 (77%) of 1320 patients received a syndromic diagnosis before the intervention, rising to 701 (86%) of 813 after the intervention (adjusted odds ratio [aOR] 1·81 [95% CI 1·40–2·34]; p<0·0001). A microbiological diagnosis was made in 294 (22%) of 1330 patients pre-intervention, increasing to 250 (30%) of 824 patients post-intervention (aOR 1·46 [95% CI 1·18–1·79]; p=0·00040). Interrupted time series analysis confirmed that these increases exceeded a modest underlying trend of improvement over time. The percentage receiving a lumbar puncture, time to appropriate therapy, and functional outcome also improved.

**Interpretation:**

Diagnosis and management of patients with suspected acute brain infections improved following introduction of a simple intervention package across a diverse range of hospitals on three continents. The intervention is now being implemented in other settings as part of the WHO Meningitis Roadmap and encephalitis control initiatives.

**Funding:**

UK National Institute for Health and Care Research.

## Introduction

Brain infections such as meningitis and encephalitis are a major cause of mortality and morbidity, with disproportionately large impact in low-income and middle-income countries (LMICs).^1^ Improved diagnosis and optimal management are thus a focus of WHO initiatives on defeating meningitis and reducing the burden of encephalitis.^2,3^ Challenges in diagnosis and management include delays in performing a lumbar puncture to confirm a brain infection syndromic diagnosis,^4–6^ ensuring the right cerebrospinal fluid (CSF) and other pathogen-specific tests are performed,^7,8^ and starting appropriate antimicrobial therapy.^9^ Even with augmented testing of patients in a research setting, microbiological diagnosis is made in at most one-third of patients,^10^ which restricts the ability to choose appropriate treatment.

The challenges in brain infection diagnosis and management, cutting across multiple elements of hospital care, require multifaceted and complex interventions.^11^ Such interventions have been attempted in higher-income settings with varying results. These have included single-country studies employing lumbar puncture packs,^7^ a meningitis care bundle,^12^ and a training-focused management intervention.^13^ In LMICs there have been a small number of studies focused on specific syndromes or target populations, but no attempt to improve care more broadly.^14,15^ In other conditions (eg, surgical site infections),^16^ multifaceted interventions have had a major effect on outcomes in LMICs; for example, an intervention combining various infection prevention measures and operating room discipline significantly reduced the risk of surgical site infections in five hospitals in Africa.^16^

To improve the diagnosis and early management of patients with suspected acute brain infections, we designed, implemented, and evaluated a tailored multifaceted intervention in hospitals in Brazil, India, and Malawi.

## Methods

### Study design and setting

We conducted this multicentre before-versus-after intervention study in 13 hospitals organised through four centres of the National Institute for Health and Care Research (NIHR) Global Health Research Group on Acute Brain Infections; the Oswaldo Cruz Foundation (Fiocruz), Recife, Brazil; the National Institute of Mental Health and Neuro Sciences, Bangalore, India; the Christian Medical College, Vellore, India; and the Malawi-Liverpool-Wellcome Trust Clinical Research Programme and Kamuzu University of Health Sciences in Blantyre, Malawi. We chose Brazil, India, and Malawi to encompass a diverse range of hospitals from countries on different continents with different World Bank income strata (upper-middle, lower-middle, and low, respectively) in order to maximise the generalisability of our findings. Of the 13 hospitals, six (46%) were located in urban areas and seven (54%) were in rural or semi-rural areas; nine (69%) were public hospitals and four (31%) were private non-profit institutions. Six hospitals (46%) provided secondary care and seven (54%) offered tertiary care; two of these seven exclusively provided specialist referral services. The number of inpatient beds ranged from 70 to 2250.

During the pre-intervention phase current practice was described using quantitative and qualitative observation of the in-hospital journeys of patients and their CSF samples, assessments of hospitals’ laboratory capacity and capability to diagnose brain infections, and simple descriptive analyses of patient data (appendix 1 pp 1–2; appendix 2 p 2). These findings informed the intervention design (appendix 2 p 3), which was developed in collaboration with an independent advisory panel, a patient and public involvement (PPI) panel comprising representatives from each country, and local and national policy makers—the latter of whom ensured only changes deemed sustainable were implemented. The study was approved by local and national ethics committees, plus the University of Liverpool Central University Research Ethics Committee (approval number 5350). Main ethical approval numbers are shown in appendix 1 (p 37). This study is registered with ClinicalTrials.gov (NCT04190303) and is complete.

### Participants

Adults aged 16 years and older and children older than 28 days presenting to study hospitals with suspected acute brain infection (meningitis, encephalitis, or both) were screened. Patients were considered eligible if they had an illness duration of less than 4 weeks, and had at least three of the following features (or two features, in the absence of an alternative explanation apparent on initial clinical assessment): fever or history of febrile illness; headache; signs of meningeal irritation (neck stiffness, photophobia, or a bulging or tense fontanelle); an altered mental state (including new confusion, disorientation, coma, or inability to talk); one or more new seizures; or new focal neurological findings. Additionally, patients not meeting these criteria could be enrolled on clinical suspicion of acute brain infection without an alternative explanatory diagnosis. Patients were excluded if they had an indwelling ventricular, meningeal, or brain implant; had undergone neurosurgery in the preceding 12 months; or had a simple febrile seizure.^17^ All patients or their legal representatives provided written informed consent or assent in accordance with Good Clinical Practice and each country’s ethics committee regulations, using forms incorporating input from our PPI panel.

### Procedures

To describe current practice and inform intervention design, we first collected sociodemographic, clinical, laboratory, imaging, and outcome data for all patients on REDCap (Vanderbilt University, Nashville TN, USA).^18^ As we did not plan to analyse subgroups or design the intervention based on race or ethnicity, these data were not recorded. Detailed real time analysis of journeys of patients from presentation to discharge, and patient CSF samples from collection to test results, were performed on at least eight patients per hospital, with space to include more patients if possible. We ensured important subgroups of patients were included in these journey observations, including age (children older and younger than 12 months and adults), sex (with self-reported options of male or female), HIV status, and how and when the patient presented to hospital. Full criteria are shown in appendix 1 (p 5). Data were collected with a bespoke tool designed with input from our PPI panel (appendix 1 p 6). Hospital laboratory capability to diagnose brain infections was assessed by a specialist team using a bespoke tool based on the WHO Laboratory Assessment Tool and Essential Diagnostics List.^19,20^ Integrated results from this exercise were presented to an intervention working group, including hospital stakeholders, who used a systematic process to design the intervention with oversight from a cross-centre team, PPI panel, policy working group, and external advisory panel (appendix 1 pp 1–2, 7–9).

We introduced three core intervention components, tailored to each hospital’s needs. First, a clinical algorithm providing decision support for diagnosis and management of patients with possible acute brain infection (appendix 2 p 4); second, a lumbar puncture pack to assist with optimal collection, transport, and storage of CSF specimens (appendix 2 p 5); and third, a microbiological testing panel using new or existing diagnostic tests, based on brain infection pathogen epidemiological data for each country and the laboratory capacity assessments (appendix 2 pp 6–8). These components were supported by training and mentoring of staff, facilitated by hospital brain infection champions who were nominated by each hospital to facilitate and advocate for the improvements in care. The pathogen detection panel used a stepwise approach which prioritised treatable and common pathogens, with support for clinical interpretation being provided in real time by the intervention team. The incremental cost to the health-care system of each additional resource (including human, material, and capital resources) required for the intervention was documented. We followed the EQUATOR template for intervention description and replication (TIDieR) guidelines for intervention description, including making our tools available on a website to allow replication and adaptation.^21^

### Outcomes

The coprimary outcomes were achievement of a syndromic diagnosis, and achievement of a microbiological diagnosis. Criteria for these diagnoses were predefined and piloted by the study team in simulated patients and the first 20 patients recruited in each centre, and assessors’ judgements were calibrated before use in this study. Syndromic diagnosis criteria were adapted from existing definitions for brain infection syndromes.^22–24^ Microbiological diagnosis was classified as confirmed, probable, or possible, based on criteria modified from earlier work.^25,26^ Each patient was assessed by two independent investigators, masked from each other’s assessment (appendix 1 pp 2–3, 10–17).

Secondary outcomes were the percentages of patients who had a lumbar puncture; time to lumbar puncture; percentage of CSF samples undergoing appropriate basic tests (CSF cell count microscopy and tests for protein, glucose, bacterial culture, and paired blood glucose); all-cause mortality at 30 days; length of stay in hospital; time to appropriate empirical and definitive anti-infective therapy (as defined by two independent assessors using country-specific and pathogen-specific criteria [appendix 1 pp 18–26]); quality of life at 30 days and at discharge from hospital using the EQ-5D-Y questionnaire for children (aged 8–15 years) and EQ-5D-3L for adults over 16 years;^27,28^ and functional outcome at discharge and 30 days, assessed using the Liverpool Outcome Score.^29^ All questionnaires were administered in a language in which the patient or their representative was fluent.

Process measures were used to assess fidelity and coverage of the intervention, including the percentage of patients having PCR or serological tests for any pathogen and the percentage of patients having tests for key pathogens in the first step of the pathogen panel.

### Statistical analysis

Qualitative patient and CSF sample journey observation data were analysed by at least two investigators using a modified framework approach to identify facilitators and challenges to optimal care.^30^ Descriptive statistics were used to summarise quantitative journey data. The integrated results, with comparison across hospitals and centres, were presented to the study group and hospital stakeholders and policy makers to inform design of the intervention.

For the primary outcomes the analysis included a comparison of percentage of patients diagnosed before versus after the intervention, as well as interrupted time series analysis.^31^ Multilevel multivariable logistic regression models were developed for the overall pooled dataset (ie, including all four centres), and for each study centre. Two models were made. The first calculated the odds ratio (OR) with 95% CIs for achievement of a diagnosis before versus after the intervention, with adjusted ORs (aORs) incorporating multiple specific covariates that could influence the likelihood of achieving a diagnosis (appendix 1 pp 3–5). The second model estimated the probability of a step change (immediate) and slope change (sustained) in diagnosis achievement due to the intervention, with aORs and p values for each (appendix 1 pp 3–4). Additional predefined subgroup analyses were performed for the larger subgroups.

Similar models were developed for secondary outcomes that were measured in percentages. For time-to-event outcomes, Kaplan–Meier curves were made, followed by multivariable Cox proportional hazards regression, with resulting univariate and adjusted hazard ratios (aHRs) and 95% CIs; death was included as a competing risk in the time-to-discharge model. Quality of life utility values were calculated using the EQ-5D-3L and EQ-5D-Y questionnaires using the UK EQ-5D-3L value set in the eq5d package in R (version 4.3.1), with results as mean difference and 95% CI, and a p value obtained using a two-sample *t* test. The lowest score in any domain of the Liverpool Outcome Score, in which 1 represents death and 5 represents full recovery, was analysed using ordinal logistic regression, with resulting aOR and 95% CI for a higher score post-intervention. As for primary outcomes, secondary outcomes were analysed for the overall dataset and for individual centres, and regression models used predefined covariates relevant for each outcome (appendix 1 pp 4–5). Process measures were compared pre-intervention versus post-intervention using χ^2^ tests.

The total cost of the intervention per centre was averaged across patients. Where there was a significant increase in the percentage of patients achieving a diagnosis, we calculated the number needed to treat (ie, the number of patients [95% CI] who would need to receive the intervention to achieve one additional diagnosis), and reported number needed to treat for harm and for benefit where relevant. All cost measures were calculated for individual centres and were reported in US$, using exchange rates on Dec 17, 2023.

Sample size was calculated using the coprimary outcome of microbiological diagnosis achievement. The percentage of patients with suspected acute brain infection receiving a microbiological diagnosis pre-intervention was estimated to be 25%, based on availability of diagnostics at included sites and a review of the literature. We set a post-intervention target percentage of 45%, on the basis that this was a plausible percentage based on published results of previous studies,^32–34^ and that this would be compelling enough to justify a change in practice. Assuming monthly assessment timepoints, 80% statistical power, an α value of 0·05, and a 1:1 ratio between the number of pre-intervention and post-intervention patients, with 8 months of observation per recruitment phase, a monthly recruitment of 55 patients would be required, resulting in a total of 450 patients pre-intervention and 450 patients post-intervention in each centre. This was calculated using previously published Stata code.^35^

Statistical analyses for all predefined outcomes were performed in R version 4.3.1. To validate the analyses of the coprimary outcomes, two statisticians who were masked to each other’s code and results performed the analyses in parallel, one using SAS version 9.4M8 and the other using R version 4.3.1.

### Role of the funding source

The funder had no role in study design, data collection, data analysis, data interpretation, or writing of the report.

## Results

**Figure 1:**
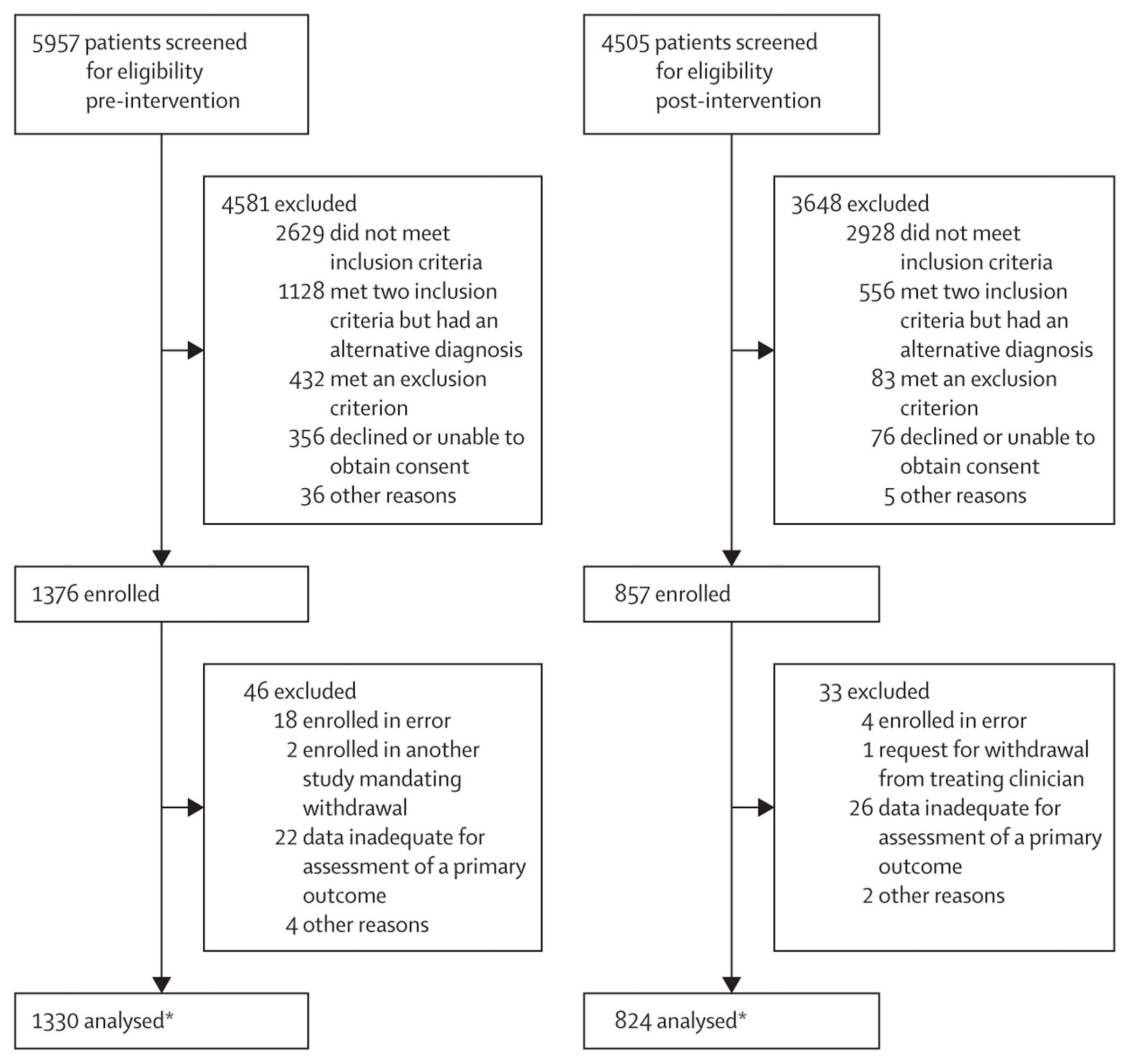
Study profile *A further ten patients who were enrolled pre-intervention and 11 patients who were enrolled post-intervention had inadequate data for assessment of syndromic diagnosis, resulting in 1320 pre-intervention patients and 813 post-intervention patients being included in the analysis for that outcome.

Between Jan 5, 2021, and Nov 30, 2022, we screened 10 462 patients and enrolled a total of 2233 patients at 13 hospital sites connected to the four study centres in Brazil, India, and Malawi (figure 1). Of the 2233 patients, 1376 (62%) were recruited before the intervention and 857 (38%) were recruited after the intervention. 2154 patients (96%) had assessment of the primary outcome (1330 [62%] patients recruited pre-intervention and 824 [38%] recruited post-intervention). Due to the COVID-19 pandemic the study was interrupted, with a resulting reduction in recruitment and a shorter post-intervention phase, the effect of which was greater in Brazil and Malawi compared with the centres in India.

Of the 2154 total patients, 1448 patients (67%) were enrolled in the two centres in India (751 [35%] in Bangalore and 697 [32%] in Vellore), 397 patients (18%) were enrolled in Brazil, and 309 (14%) were enrolled in Malawi (table 1). The median age across centres was 23 years (IQR 6–44), with 1276 (59%) being adults aged 16 years or older and 888 (41%) children aged between 29 days and 15 years, which was similar across centres (54–67%). The majority of patients were male (1264 [59%] *vs* 890 [41%] female), resided in an urban area (1183 patients [55%]), and were transferred to a study hospital from another hospital (1220 patients [57%]), although only 526 (24%) of 2150 had documentation of receiving antimicrobial therapy before presenting at the study hospital. 202 patients (10%) of the 2094 with available data had HIV infection at presentation.

From the 2137 patients with complete data on clinical features, the most common clinical presentations across centres were possible meningoencephalitis (755 [35%]) and possible encephalitis (748 [35%]; defined in appendix 1 p 11). The majority of patients (1377 [64%] of 2152) presented within 7 days of illness onset; 1787 patients (83%) of 2154 had fever and 1613 (75%) had an altered mental state. Other common clinical features included 1106 patients (51%) with headache, 996 (46%) with seizures, and 873 (41%) with focal neurological signs; 936 (44%) of 2137 patients with available data presented with features of meningeal irritation. These findings were broadly similar for patients before and after the intervention, but differed between study centres (table 1): Malawi had more patients presenting within 7 days and with encephalopathy than other centres, and had higher HIV prevalence; Malawi and Bangalore had fewer patients residing in urban areas; transfer from another hospital was more common in Brazil and Bangalore; and previous treatment was most common in Bangalore.

Overall, a brain infection syndromic diagnosis was achieved in 1428 (66%) of 2154 patients, among whom meningoencephalitis was the most common confirmed syndrome (629 [44%] of 1428), followed by encephalitis (561 [39%]) and meningitis (159 [11%]); a non-brain infection syndrome was diagnosed in 293 (14%) of 2154 patients (appendix 2 p 22).

**Figure 2:**
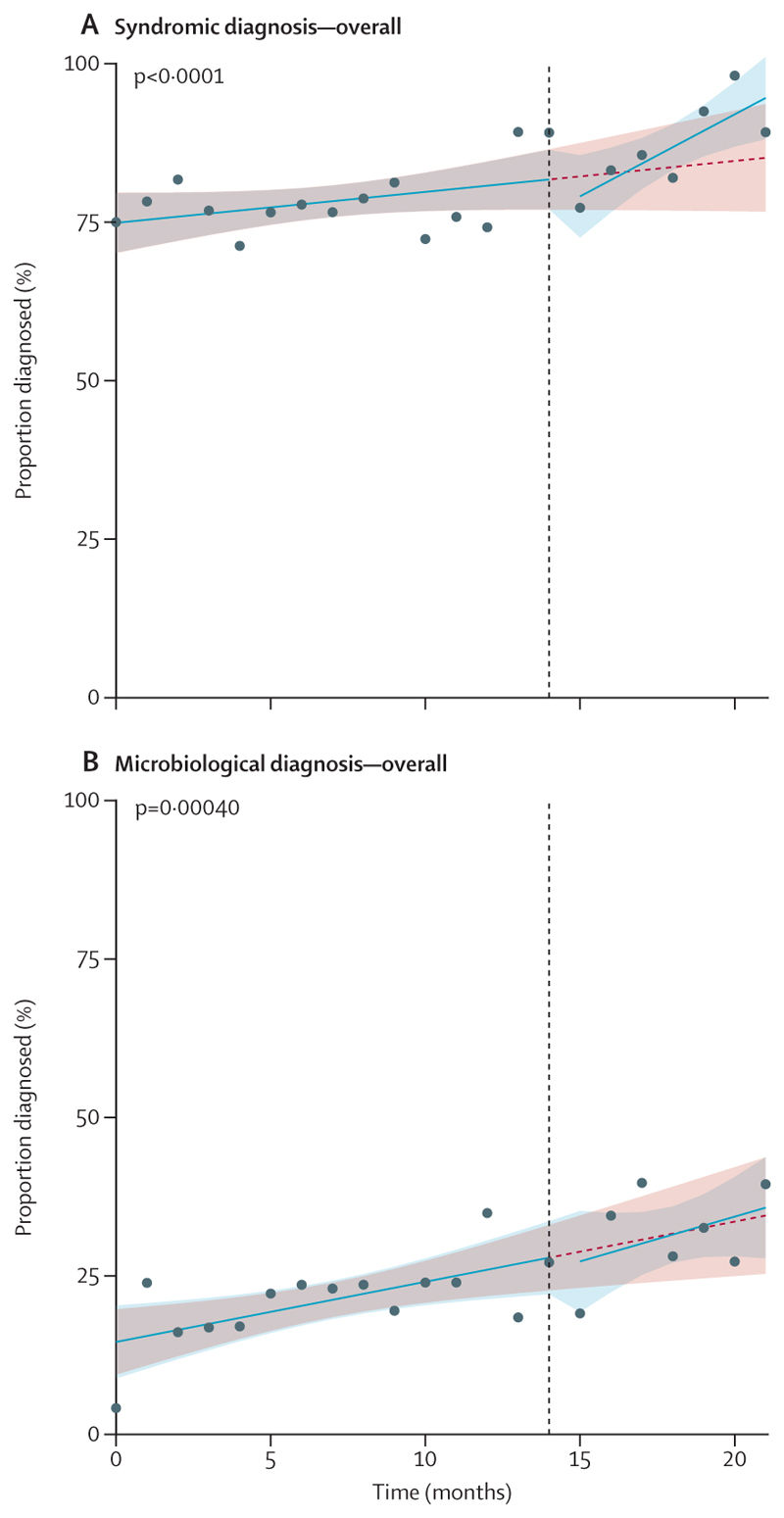
Achievement of the co-primary outcomes of syndromic diagnosis and microbiological diagnosis (A) Percentage of patients achieving a syndromic diagnosis, per month, pooled across all centres. (B) Percentage of patients achieving a microbiological diagnosis, per month, pooled across all centres. Dots represent percentages of patients achieving a diagnosis in each month of recruitment. The blue line represents the observed trend across these points. The shaded blue around this line represents the 95% CI around these percentages. The dashed red line represents the counterfactual situation: a predicted trend assuming no intervention was delivered, based on pre-intervention data. The shaded red around this line represents the 95% CI around these percentages. The vertical dashed black line represents the timepoint at which the intervention was implemented.

The total percentage of patients receiving a syndromic diagnosis increased significantly from 1020 (77%) of 1320 before the intervention to 701 (86%) of 813 after the intervention (aOR 1·81 [95% CI 1·40–2·34]; p<0·0001; table 2). When hospital was included as an additional variable in the analysis, the effect remained similar (1·65 [1·24–2·18]; p=0·00049; appendix 2 p 23). Most of the increased diagnoses were of brain infection syndromes, from 62% pre-intervention to 73% post-intervention (825 of 1330 to 603 of 824; appendix 2 p 22). The interrupted time series analysis showed an increase in the slope of the percentages diagnosed per month, on top of a modest underlying trend of improvement over time (p=0·027; figure 2, table 2).

The most common confirmed or probable microbiological diagnoses were *Orientia tsutsugamushi* (scrub typhus) in the Indian centres, *Streptococcus pneumoniae* in Brazil, and *Cryptococcus neoformans* in Malawi. Overall, bacteria were more commonly identified than other pathogens, with *Mycobacterium tuberculosis* and *S pneumoniae* diagnoses being made in all centres (appendix 2 pp 10, 16). Some viruses were only diagnosed post-intervention: adenovirus, chikungunya, cyto-megalovirus, Epstein–Barr virus, enterovirus, polyomavirus 2 (also known as John Cunningham [JC] virus) and West Nile virus. *M tuberculosis* and *C neoformans* were more common in patients with HIV, whereas *O tsutsugamushi* and *S pneumoniae* were more common in patients without HIV. Pathogens tested for in the first step of the testing panel accounted for 78% of confirmed and probable diagnoses.

For the four centres together, a significant increase in microbiological diagnosis was observed, from 294 (22%) of 1330 patients pre-intervention to 250 (30%) of 824 post-intervention (aOR 1·46 [95% CI 1·18–1·79]; p=0·00040; table 2). The slope of improvement increased significantly post-intervention (p=0·026; figure 2). When adjusting for hospital site, the before-versus-after increase (1·33 [1·07–1·65]; p=0·0089) and slope change (p=0·048) remained similar. The before-versus-after increase also remained significant when including possible causative pathogens in addition to probable and confirmed pathogens (517 [39%] of 1330 to 417 [51%] of 824, aOR 1·46 [95% CI 1·21–1·77]; p<0·0001). For patients with HIV, 44 (39%) of 113 were diagnosed with a probable or confirmed pathogen, which increased to 43 (48%) of 89 post-intervention.

When the primary outcomes were analysed for adults and children as distinct subgroups, similar before-versus-after analysis results were observed, for both syndromic diagnosis (adults aOR 1·63, [95% CI 1·16–2·31], p=0·0055 and children 2·00 [1·35–2·97], p=0·00055) and for microbiological diagnosis (adults aOR 1·36 [1·03–1·78], p=0·028; children 1·58 [1·14–2·20], p=0·0063; appendix 2 p 23). When HIV infection was added to the models as a covariate in a post hoc sensitivity analysis, the results also remained similar (aOR 1·73 [95% CI 1·33–2·25] for syndromic diagnosis and 1·42 [1·15–1·76] for microbiological diagnosis; appendix 2 p 23).

Results for secondary outcomes are summarised in table 3 for the overall data, and for each centre individually in appendix 2 (pp 17–19). Multiple secondary outcomes improved post-intervention when combining all four centres, in line with the observed increases in diagnoses. The percentage of patients having a lumbar puncture increased from 1055 (79%) of 1330 patients pre-intervention to 733 (89%) of 824 patients post-intervention (aOR 2·13 [95% CI 1·63–2·77]; p<0·0001), with interrupted time series analysis confirming a significant step change (p=0·00016). Time to lumbar puncture shortened post-intervention from a median 13 h (IQR 3–41) to 9 h (3–28; aHR 1·34, 1·22–1·49; p<0·0001; appendix 2 p 12). Time to appropriate empirical therapy was significantly shorter post-intervention: 977 (73%) of 1330 versus 673 (82%) of 824 patients received this on the day of, or the day after, presentation (aHR 1·15 [95% CI 1·04–1·29]; p=0·0093, appendix 2 p 13). Liverpool Outcome Score at discharge just met statistical significance for higher values post-intervention compared with pre-intervention (aOR 1·22 [95% CI 1·00–1·49]; p=0·049). This improved score was confirmed at 30-day follow-up (1·36 [1·08–1·71]; p=0·0088; table 3). While the median score did not change pre-intervention versus post-intervention, at discharge there was a modest increase in patients achieving a good outcome (mild or no disability) from 352 (36%) of 970 to 198 (39%) of 511, and at follow-up the percentage of patients with a good outcome was larger: 464 (59%) of 786 versus 270 (67%) of 402. There was no significant difference in other secondary outcomes in the overall dataset, including mortality (table 3).

Centre-level analyses demonstrated improvements for a range of different outcomes. For the coprimary outcomes—syndromic diagnosis and microbiological diagnosis—increases in the percentage of patients achieving these two measures were observed in three of the four centres (syndromic diagnoses improved in Vellore, Brazil, and Malawi and microbiological diagnoses improved in Bangalore, Vellore, and Malawi). In each centre, either syndromic diagnosis or microbiological diagnosis increased post-intervention, although the size of the effect varied between centres (table 2). For the centres with higher recruitment, this was associated with a significant increase on multivariable analysis, for example syndromic diagnosis in Vellore (2·34 [1·30–4·21]; p=0·0048) and microbiological diagnosis in the Bangalore centre (1·60 [1·08–2·35]; p=0·018; table 2; appendix 2 pp 9, 11). Interrupted time series analysis also detected a significant step increase for syndromic diagnoses in Vellore.

Of the secondary outcomes, increases in the number of lumbar punctures being performed was observed across all centres; reduction in time to lumbar puncture was observed in three of the four centres (appendix 2 p 12). The percentage of patients receiving appropriate empirical therapy on the day of, or the day after, presentation increased in all centres, although the medians remained the same; in Vellore this increase was confirmed in the multivariable analysis (appendix 2 p 13, 18). Time to discharge from hospital was significantly shorter post-intervention in Brazil, but not in other centres (appendix 2 pp 17–19). EQ-5D scores increased in Brazil at discharge and follow-up, but not in other centres. Analyses for Liverpool Outcome Score suggested an improvement post-intervention in Bangalore and Brazil (appendix 2 p 19).

Process measures showed an increase in pathogen tests pre-intervention versus post-intervention overall and in each centre (all but one were significant statistically; appendix 2 p 20): 433 (33%) of 1330 patients versus 616 (75%) of 824 patients received any PCR test, 714 (54%) versus 677 (82%) received any serological test, and 97 (7%) versus 499 (61%) received tests for priority pathogens. Examples of pathogens diagnosed more frequently post-intervention include *O tsutsugamushi* from 14 (4%) of 380 to 41 (11%) of 371 and dengue from two (1%) of 380 to ten (3%) of 371 in Bangalore; and *S pneumoniae* with 12 (3%) of 451 pre-intervention versus 17 (7%) of 246 post-intervention in Vellore.

The average cost of the intervention per patient was US$112 in Bangalore, $257 in Malawi, $291 in Vellore, and $302 in Brazil (appendix 2 p 20). The number needed to treat (ie, number of patients needing to receive the intervention in order to achieve one additional diagnosis) was 11 patients (95% CI 8–18) for syndromic diagnosis and 12 patients (8–22) for microbiological diagnosis.

## Discussion

Our multifaceted intervention increased the percentage of patients with suspected acute brain infections achieving a syndromic diagnosis from 77% to 86% and a microbiological diagnosis from 22% to 30% across a variety of hospitals in Brazil, India, and Malawi. This improvement was maintained when adjusting for underlying trends and multiple potential confounders and in our post hoc sensitivity analyses. While the absolute increase in diagnosis was modest, especially for syndromic diagnoses, this is true of most real-life studies evaluating interventions delivered at a system level.^36^ There were also improvements in time to lumbar puncture and time to appropriate empirical anti-infective therapy. Functional outcome as measured using a dichotomised Liverpool Outcome Score improved, but the median remained unchanged, suggesting that any improvement might have been small.

In addition to improving across the four centres analysed as a whole, syndromic or microbiological diagnosis also increased in each centre when analysed individually. Adjusted analyses showed significant increases in Bangalore for microbiological diagnosis and Vellore for syndromic diagnosis; other increases were not significant after adjustment for covariates in Brazil and Malawi. Other outcomes showed significant improvements, and the direction of effect (ORs >1) was similar across centres in most outcomes, although the size of the effect varied between centres. This suggests that the intervention was effective, and is probably generalisable across LMIC settings, but sample size limitations prevented this from being confirmed in analyses adjusting for multiple variables, especially in the centres in Brazil and Malawi which had lower recruitment.

A key attribute of the intervention, which probably contributed to its effectiveness in different settings, was that it was designed and implemented by local clinicians and administrative leads themselves, based on published literature and local knowledge. Core components were simple, and included a clinical algorithm, a lumbar puncture pack, and structured pathogen testing, accompanied by short (from 1 h to 1·5 days) training and orientation to the intervention. This intervention package is now being deployed more widely with tailoring to each new hospital.

The increased number of diagnoses were likely the result of improvements in key investigations such as the number and speed of lumbar punctures, and increased pathogen testing.^37^ Several pathogens were detected more frequently post-intervention. *O tsutsugamushi*, the cause of scrub typhus, is recognised increasingly across southeast Asia and is treatable. Few patients with CNS scrub typhus have the classically described eschar, and so rather than testing only when there is clinical suspicion, we propose scrub typhus be included as a first-line test in algorithms for diagnosing brain infections in endemic countries.^38,39^ Diagnosis of *S pneumoniae* and *M tuberculosis* might have increased due to quicker performance of lumbar punctures and more appropriate volumes and transport of CSF, all of which are known to increase yield.^40,41^ Additionally the introduction or systematic use of PCR tests (introducing testing for these pathogens for the first time to some hospitals, where culture is not available) probably contributed to the improvements in number of diagnoses.

Some viruses, only diagnosed after the introduction of the intervention, are of particular public health importance: enteroviruses are long-standing causes of devastating epidemics, while chikungunya has recently emerged globally as a CNS pathogen.^42,43^ The increase in Liverpool Outcome Score observed post-intervention was consistent with improved diagnoses leading to better management, including appropriate antimicrobial therapy. Even though the neurological functional outcome score improved, the overall effect appeared to be modest and the study was not powered to investigate this nor the other downstream outcomes of mortality and quality of life, which did not change post-intervention.

One strength of our approach is that it led to an improvement in diagnosis and early management of adults and children (aged >28 days) with a range of brain infection presentations across various hospital types in countries representing each of the World Bank’s LMIC groups. Previous studies of multifaceted interventions for brain infections have focused on specific age groups or syndromes, and have mostly been conducted in single high-income countries with modest sample sizes. In the UK, one single-centre study from our group showed an improvement in CSF testing in patients with suspected brain infections using a lumbar puncture pack;^7^ in another UK-based multicentre cluster-randomised trial we could not demonstrate any benefit from a training-focused package for improving encephalitis management.^13^ Two studies implementing a care bundle for bacterial meningitis in adults reported improvements in emergency management, with one study from Italy describing a possible mortality benefit, although without adjusting for recruitment over several years;^12^ and the other reported improved delivery of protocols in Malawi, but did not demonstrate an effect on diagnosis or clinical outcomes.^14^ A multifaceted intervention for improving diagnosis demonstrated reduced mortality in adults living with HIV in Tanzania and Cameroon, but not in Malawi.^15^

By involving hospital management staff, patient and public representatives, and local and national policy makers from the start, our intervention was designed with feasibility and sustainability in mind. Many previous studies of CNS infections in LMICs have increased the diagnostic yield for study patients through extensive microbiological investigations,^38,44^ or next-generation sequencing,^45^ but the investigations are not then available subsequently for routine patient care.^46^ Although giving important information about the range of brain infection aetiologies, these interventions do little to help the management of future patients. In contrast, we only included interventions that policy makers agreed would be affordable beyond the end of the study, and we embedded them into routine care to help ensure their sustainability. While the absolute increases in diagnosis were modest, the relative increase in microbiological diagnosis of over one-third means that scale-up of the intervention is likely to have a large effect at a population level.

Although focused on brain infections, many elements of our intervention, for example on sample transport and processing and laboratory capacity, will improve the diagnosis of infections more broadly. Unusual clusters of patients with brain infections can sometimes herald the onset of emerging infections,^47–49^ that often occur in LMICs with poor diagnostic capabilities. Our approach to improving brain infection diagnostic capabilities should be broadened to include other infection syndromes, thus strengthening near-patient capacity for identifying emerging infections in LMICs, as advised by WHO.^50^ Improving diagnosis of infections also enables focusing of therapy, enhancing antimicrobial stewardship efforts to address the global threat of antimicrobial resistance.

The study has some limitations. Its duration and recruitment were affected by the COVID-19 pandemic, resulting in an inability to achieve the planned sample size in individual centres and overall. Despite this, the study results proved to be significant statistically not only with a before-versus-after comparison, but also using a more stringent interrupted time series analysis. We were able to assess up to 7 months after the intervention; longer-term sustainability will need further reinforcement and refresher training, as with many clinical care initiatives.

Our study has shown that improvements in brain infection diagnosis and management in LMICs are possible with simple optimisation of routine hospital care. We delivered significant changes across the four centres, even though the settings were quite different; this underscores the generalisability of our approach. There are now plans to roll out the intervention to new centres and countries, supported by WHO’s *Defeating Meningitis by 2030* global roadmap, and its initiative to reduce the burden of encephalitis.^2,3^ The Brain Infections Global Diagnostic Toolkit including clinical algorithms, guidance on creating a lumbar puncture pack and a pathogen testing panel, patient and sample flow charts, and the laboratory capacity assessment tool is now freely available on the Brain Infections Global website, adaptable for local settings.

## Supplementary Material

Supplementary Materials

## Figures and Tables

**Table 1:** Demographic and clinical characteristics

	Pre-intervention cohort						Post-intervention cohort				
	Bangalore, India (n=38O)	Vellore, India (n=451)	Brazil (n=29O)	Malawi (n=2O9)	Overall (n=133O)		Bangalore (n=371)	Vellore (n=246)	Brazil (n=107)	Malawi (n=100)	Overall (n=824)
Age, years	27 (10-45)	19 (4-46)	26 (9-46)	27 (6-41)	25 (6-45)		25 (8-45)	21 (3-45)	6 (3-18)	30 (6-42)	21 (5-43)
Age group											
Child aged <lyear	38 (10%)	47 (10%)	20 (7%)	4 (2%)	109 (8%)		20 (5%)	39 (16%)	11 (10%)	2 (2%)	72 (9%)
Child aged 1-4years	31 (8%)	75 (17%)	30 (10%)	42 (20%)	178 (13%)		40 (11%)	32 (13%)	28 (26%)	20 (20%)	120 (15%)
Child aged 5-15 years	68 (18%)	84 (19%)	58 (20%)	24 (11%)	234 (18%)		86 (23%)	37 (15%)	38 (36%)	14 (14%)	175 (21%)
Adult aged 16-59 years	201 (53%)	173 (38%)	147 (51%)	129 (62%)	650 (49%)		193 (52%)	109 (44%)	26 (24%)	59 (59%)	387 (47%)
Adult aged ≥60years	42 (11%)	72 (16%)	35 (12%)	10 (5%)	159 (12%)		32 (9%)	29 (12%)	4 (4%)	5 (5%)	70 (8%)
Sex											
Female	158 (42%)	194 (43%)	125 (43%)	78 (37%)	555 (42%)		163 (44%)	83 (34%)	37 (35%)	50 (50%)	333 (40%)
Male	222 (58%)	257 (57%)	165 (57%)	131 (63%)	775 (58%)		207 (56%)	163 (66%)	69 (65%)	50 (50%)	489 (59%)
Urban residence	114 (30%)	294 (65%)	250 (86%)	51 (25%)	709 (53%)		117 (32%)	222 (90%)	85 (79%)	50 (50%)	474 (58%)
HIV infection*	24/378 (6%)	6/449 (1%)	18/227 (6%)	65/191 (31%)	113/1295 (8%)		32/368 (9%)	10/246 (4%)	2/91 (2%)	45/94 (45%)	89/799 (11%)
Transferred from another hospital	304(80%)	134 (30%)	261 (90%)	51 (24%)	750 (56%)		282 (76%)	67 (27%)	93 (87%)	28 (28%)	470 (57%)
Received anti-infective therapy previously^†^	157/379 (41%)	100/451 (22%)	17/290 (6%)	36/209 (17%)	310/1329 (23%)		123/369 (33%)	50/246 (20%)	19/106 (18%)	24/100 (24%)	216/821 (26%)
Clinical presentation^‡^											
Possible encephalitis	124/379 (33%)	183/451 (41%)	84/285 (29%)	76/206 (36%)	467/1321 (35%)		134/367 (36%)	91/246 (37%)	24/105 (23%)	32/98 (32%)	281/816 (34%)
Possible meningitis	24/379 (6%)	26/451 (6%)	39/285 (14%)	14/206 (7%)	103/1321 (8%)		17/367 (5%)	18/246 (7%)	27/105 (26%)	16/98 (16%)	78/816 (9%)
Possible meningoencephalitis	159/379 (42%)	178/451 (39%)	79/285 (28%)	52/206 (25%)	468/1321 (35%)		147/367 (40%)	99/246 (40%)	17/105 (16%)	24/98 (24%)	287/816 (35%)
Encephalopathy	57/379 (15%)	59/451 (13%)	62/285 (22%)	57/206 (27%)	235/1321 (18%)		62/367 (17%)	30/246 (12%)	13/105 (12%)	23/98 (23%)	128/816 (16%)
Other	15 (4%)	5 (1%)	21 (7%)	7 (3%)	48 (4%)		7 (2%)	8 (3%)	24 (23%)	3 (3%)	42 (5%)
Duration of illness𝕊											
Acute (<7 days)	208/380 (55%)	314/451 (70%)	170/290 (59%)	171/208 (82%)	863/1329 (65%)		193/371 (52%)	167/246 (68%)	81/107 (76%)	73/99 (74%)	514/823 (62%)
Subacute (7-28 days)	172/380 (45%)	137/451 (30%)	120/290 (41%)	37/208 (18%)	466/1329 (35%)		178/371 (48%)	79/246 (32%)	26/107 (24%)	26/99 (26%)	309/823 (38%)
Clinical features at presentation											
Fever	298 (78%)	402 (89%)	193(67%)	162 (78%)	1055 (79%)		322 (87%)	232 (94%)	94 (88%)	84 (84%)	732 (89%)
Headache	186 (49%)	168 (37%)	213 (73%)	123 (59%)	690 (52%)		169 (46%)	106 (43%)	72 (67%)	69 (69%)	416 (50%)
Features of meningeal irritation^¶^	183/379 (48%)	204/451 (45%)	118/285 (41%)	66/206 (32%)	571/1321 (43%)		164/367 (45%)	117/246 (48%)	44/105 (42%)	40/98 (41%)	365/816 (44%)
Altered mental state	292 (77%)	375 (83%)	205 (71%)	143 (68%)	1015 (76%)		297 (80%)	189 (77%)	47 (44%)	65 (65%)	598 (73%)
Seizure^∥^	164 (43%)	234 (52%)	115 (40%)	133 (64%)	646 (49%)		167 (45%)	122 (50%)	18 (17%)	43 (43%)	350 (42%)
Focal neurological sign^∥^	173 (46%)	184 (41%)	123 (42%)	57 (27%)	537 (40%)		199 (54%)	91 (37%)	21 (20%)	25 (25%)	336 (41%)
Reduced consciousness level	224 (59%)	313 (69%)	169 (58%)	109 (52%)	815 (61%)		229 (62%)	153 (62%)	37 (35%)	50 (50%)	469 (57%)
Rash or eschar	43 (11%)	47 (10%)	9 (3%)	5 (2%)	104 (8%)		17 (5%)	17 (7%)	5 (5%)	5 (5%)	44 (5%)

Data are n (%), n/N (%), or median (IQR).

* 60 patients (3%) had a missing value for HIV infection.

† Four patients (<1%) had a missing value for previous therapy.

‡ 17 patients (1%) had a missing value for clinical presentation.

¶ Two patients (<1%) had a missing value for duration of illness at the point of presentation to the study hospital. ¶17 patients (1%) had a missing value for features of meningeal irritation.

∥ Single or multiple.

**Table 2:** Primary outcome results by country and location pre-intervention and post-intervention

	Pre-intervention, n/N (%)	Post-intervention, n/N (%)	Univariate analysis pre-intervention versus post-intervention			Multivariable analysis pre-intervention versus post-intervention			Step change			Slope change		
			OR (95% CI)	p value		aOR (95% CI)	p value		aOR (95% CI)	p value		aOR (95% CI) of pre-intervention slope	aOR (95% CI) of post-intervention slope	p value
**Syndromic diagnosis achieved**														
Bangalore, India	325/379 (86%)	309/367 (84%)	0.89 (0.59-1.32)	0.55		1.30 (0.76-2.23)	0.34		0.38 (0.08-1.73)	0.21		1. 10 (0.93-1.30)	1.22 (0.99-1.51)	0.13
Vellore, India	390/450 (87%)	229/246 (93%)	2.07 (1-21-3.74)	0.011*		2.34 (1.30-4.21)	0.0048*		3.51 (1.17-10.57)	0.026*		0.99 (0.91-1.07)	0.90 (0.70-1.16)	0.68
Brazil	220/285 (77%)	98/103 (95%)	5.79 (2.48-16.93)	0.00025*		2.03 (0.70-5.88)	0.19		0.76 (0.13-4.33)	0.76		1.07 (0.96-1.19)	1.25 (0.75-2.10)	0.36
Malawi	85/206 (41%)	65/97 (67%)	2.89 (1.76-4.84)	<0.0001*		1.41 (0.69-2.89)	0.35		2.09 (0.58-7.57)	0.26		1.02 (0.93-1.12)	0.82 (0.59-1.15)	0.48
Overall	1020/1320 (77%)	701/813 (86%)	1.84 (1.46-2.34)	<0.0001*		1.81 (1.40-2.34)	<0.0001*		0.99 (0.59-1.67)	0.98		1.02 (0.98-1.07)	1.15 (1.02-1.29)	0.027*
**Microbiological diagnosis achieved**														
Bangalore, India	79/380 (21%)	119/371 (32%)	1.80 (1.30-2.51)	0.00049*		1.60 (1.08-2.35)	0.018*		0.67 (0.20-2.23)	0.52		1.06 (0.93-1.20)	1.17 (1.01-1.36)	0.12
Vellore, India	143/451 (32%)	86/246 (35%)	1.16 (0.83-1.61)	0.38		1.14 (0.81-1.62)	0.45		0.82 (0.41-1.62)	0.57		1.05 (0.99-1.11)	1.01 (0.87-1.16)	0.24
Brazil	28/290 (10%)	11/107 (10%)	1.07 (0.49-2.18)	0.85		0.50 (0.21-1.19)	0.12		1.89 (0.32-11.11)	0.48		0.99 (0.87-1.12)	0.68 (0.47-1.00)	0.14
Malawi	44/209 (21%)	34/100 (34%)	1.93 (1.13-3.28)	0.015*		1.20 (0.65-2.22)	0.55		0.95 (0.27-3.28)	0.93		1.05 (0.96-1.15)	0.94 (0.69-1.28)	0.52
Overall	294/1330 (22%)	250/824 (30%)	1.53 (1.26-1.87)	<0.0001*		1.46 (1.18-1.79)	0.00040*		0.88 (0.57-1.37)	0.58		1.05 (1.01-1.09)	1.06 (0.97-1.15)	0.026*

aOR=adjusted odds ratio. OR=odds ratio.

* Statistically significant change.

**Table 3:** Secondary outcome results on the overall dataset

	Pre-intervention (n=1330)	Post-intervention (n=824)
Lumbar puncture performed		
Patients, n (%)	1055 (79%)	733 (89%)
Univariate OR (95% CI) versus	NA	2 ·10 (1 ·63 to 2 ·72)
pre-intervention Univariate p value	NA	<0 ·0001
aOR (95% CI) versus pre-intervention	NA	2 ·13 (1-63 to 2 ·77)
Adjusted p value	NA	<0 ·0001
Step change versus pre-intervention*	NA	0 ·00016
Slope change versus pre-intervention*	NA	0 ·081
Time to lumbar puncture, h		
Median (IQR)	13 (3 to 41)	9 (3 to 28)
Univariate HR (95% CI) versus	NA	1 ·38 (1 ·25 to 1 ·52)
pre-intervention Univariate p value	NA	<0 ·0001
aHR (95% CI) versus pre-intervention	NA	1 ·34 (1 ·22 to 1 ·49)
Adjusted p value	NA	<0 ·0001
Basic CSF tests performed—allf		
Participants with all CSF tests performed,	448/1049 (43%)	324/730 (44%)
n/N (%) Univariate OR (95% CI) versus	NA	1 ·09 (0 ·90 to 1 ·32)
pre-intervention		
Univariate p value	NA	0 ·39
aOR (95% CI) versus pre-intervention	NA	0 ·93 (0 ·70 to 1 ·22)
Adjusted p value	NA	0 ·59
Basic CSF tests performed—excluding paired blood glucoset		
Participants with all CSF tests performed,	581/1049 (55%)	471/730 (65%)
n/N (%)		
Univariate OR (95% CI) versus	NA	1 ·47 (1 ·21 to 1 ·79)
pre-intervention		
Univariate p value	NA	0 ·00010
aOR (95% CI) versus pre-intervention	NA	1 ·02 (0 ·79 to 1 ·32)
Adjusted p value	NA	0 ·88
Time to appropriate empirical therapy, days		
Median (IQR)	0 (0 to 1)	0 (0 to 1)
Received on day of, or day after, presentation	977 (73%)	673 (82%)
Univariate HR (95% CI) versus	NA	1 ·16 (1 ·04 to 1 ·29)
pre-intervention		
Univariate p value	NA	0 ·0056
aHR (95% CI) versus pre-intervention	NA	1 ·15 (1 ·04 to 1 ·29)
Adjusted p value	NA	0 ·0093
Time to appropriate definitive therapy, days		
Median (IQR)	0 (0 to 1)	0 (0 to 1)
Univariate HR (95% CI) versus	NA	0·88 (0·72 to 1·06)
pre-intervention		
Univariate p value	NA	0·18
aHR (95% CI) versus pre-intervention	NA	0·97 (0·79 to 1·20)
Adjusted p value	NA	0·79
Time to discharge from hospital, days		
Median (IQR)	7 (4 to 13)	7 (4 to 12)
Univariate HR (95% CI) versus pre-intervention	NA	1-11 (1 ·00 to 1-23)
Univariate p value	NA	0 ·041
aHR (95% CI) versus pre-intervention	NA	1 ·04 (0-94 to 1-14)
Adjusted p value	NA	0 ·48
EQ-5D score at discharge	794 (60%)	396 (48%)
Mean (SD)	0 ·58 (0.47)	0 ·56 (0.47)
Mean difference (95% CI) versus pre-intervention	NA	-0 ·02 (-0 ·08 to 0 ·03)
p value	NA	0 ·40
EQ-5D score at follow-up	668 (50%)	332 (40%)
Mean (SD)	0 ·76 (0.34)	0 ·79 (0 ·39)
Mean difference (95% CI) versus pre-intervention	NA	0 ·02 (-0 ·02 to 0 ·07)
p value	NA	0 ·32
Liverpool Outcome Score at discharge	970 (73%)	511 (62%)
Median (IQR)	3 (2 to 5)	3 (2 to 5)
Good outcome^‡^	352/970 (36%)	198/511 (39%)
aOR (95% CI) versus pre-intervention	NA	1 ·22 (1 ·00 to 1-49)
Adjusted p value	NA	0 ·049
Liverpool Outcome Score at follow-up	786 (59%)	402 (49%)
Median (IQR)	4 (3 to 5)	4 (3 to 5)
Good outcome, n/N (%)^‡^	464/786 (59%)	270/402 (67%)
aOR (95% CI) versus pre-intervention	NA	1 ·36 (1 ·08 to 1-71)
Adjusted p value	NA	0 ·0088
Mortality		
Partients, n (%)	96 (7%)	58 (7%)
Univariate OR (95% CI) versus pre-intervention	NA	0 ·98 (0 ·69 to 1 ·36)
Univariate p value	NA	0 ·88
aOR (95% CI) versus pre-intervention	NA	0 ·99 (0 ·69 to 1 ·41)
Adjusted p value	NA	0 ·95

aHR=adjusted hazard ratio. aOR=adjusted odds ratio. CSF=cerebrospinal fluid. HR=hazard ratio. NA=not applicable. OR=odds ratio.

* From interrupted time series analysis, performed only for outcomes measured as proportions, for which a pre-intervention versus post-intervention comparison yielded a significant improvement.

† Only includes patients who had a lumbar puncture; of these, data on appropriate tests were not available for six (1%) of 1055 patients pre-intervention and three (<1%) of 733 participants post-intervention.

‡ A Liverpool Outcome Score of 4 or 5 was considered a good outcome.

## Data Availability

Data can be requested from BS and TS at biglobal@liverpool.ac.uk. De-identified participant data, a data dictionary, and other specified datasets can be requested. The study protocol, statistical analysis plan, and informed consent form will also be made available on request. Specific requests for data will require the submission of a proposal with a valuable research question as assessed by the study steering committee. A data access agreement should be signed.

## Research in context

### Evidence before this study

We searched PubMed and Cochrane CENTRAL from database inception to May 31, 2024, for studies reporting intervention packages for diagnosis or management of patients with acute brain infections, with no language restrictions, using a predefined search strategy including the terms “bundle”, “package”, and “intervention”, in combination with one of the following: “complex”, “multifaceted”, “system”, “service”, or “quality”. We found six relevant studies, with publication dates ranging from 1998 to 2023; these mostly focused on single countries (predominantly high income), age groups, or syndromes, and thus did not have broad applicability. While some improvements in outcomes were reported, only two studies targeted both diagnosis and management, and none were generalisable across low-income and middle-income countries.

### Added value of this study

We developed a simple, context-informed, multifaceted clinical and laboratory intervention, in collaboration with local and national policy makers and patient, public, and hospital stakeholders, to improve diagnosis and management of patients with suspected acute brain infections in 13 hospitals in Brazil, India, and Malawi. The intervention included a diagnostic and management algorithm, a lumbar puncture pack, testing panel, and staff training. It improved the percentage of patients with a syndromic diagnosis and the percentage with a microbiological diagnosis (the two co-primary outcomes) across the three countries. Improvements were observed in secondary outcomes focusing on management and functional measures. Numbers needed to treat for achievement of an additional diagnosis were favourable.

### Implications of all the available evidence

This study shows that a package of simple enhancements to usual care improved diagnosis and management of patients with suspected acute brain infections presenting to hospitals in low-income and middle-income countries. With the involvement of national and international policy makers from the outset, the intervention is already feeding into national practice guidelines and WHO meningitis and encephalitis initiatives, and was designed to be scaled up internationally. The approach is also being adapted to improve diagnosis of other severe infection syndromes, and to support detection of emerging infections.

## References

[1] Hay SI, Abajobir AA, Abate KH (2017). Global, regional, and national disability-adjusted life-years (DALYs) for 333 diseases and injuries and healthy life expectancy (HALE) for 195 countries and territories, 1990–2016: a systematic analysis for the Global Burden of Disease Study 2016. Lancet.

[2] WHO (2023). Why encephalitis matters? Report of the virtual meeting, 28–29 June 2022.

[3] WHO (2021). Defeating meningitis by 2030: a global road map.

[4] Michael B, Menezes BF, Cunniffe J (2010). Effect of delayed lumbar punctures on the diagnosis of acute bacterial meningitis in adults. Emerg Med J.

[5] Salazar L, Hasbun R (2017). Cranial imaging before lumbar puncture in adults with community-acquired meningitis: clinical utility and adherence to the infectious diseases Society of America guidelines. Clin Infect Dis.

[6] Saylor D, Elafros M, Bearden D (2023). Patient, provider, and health systems factors leading to lumbar puncture nonperformance in Zambia: a qualitative investigation of the “tap gap”. Am J Trop Med Hyg.

[7] Michael BD, Powell G, Curtis S (2013). Improving the diagnosis of central nervous system infections in adults through introduction of a simple lumbar puncture pack. Emerg Med J.

[8] Ellis J, Harvey D, Defres S (2022). Clinical management of community-acquired meningitis in adults in the UK and Ireland in 2017: a retrospective cohort study on behalf of the National Infection Trainees Collaborative for Audit and Research (NITCAR). BMJ Open.

[9] Bodilsen J, Dalager-Pedersen M, Schønheyder HC, Nielsen H (2016). Time to antibiotic therapy and outcome in bacterial meningitis: a Danish population-based cohort study. BMC Infect Dis.

[10] Ravi V, Hameed SKS, Desai A (2022). An algorithmic approach to identifying the aetiology of acute encephalitis syndrome in India: results of a 4-year enhanced surveillance study. Lancet Glob Health.

[11] Skivington K, Matthews L, Simpson SA (2021). A new framework for developing and evaluating complex interventions: update of Medical Research Council guidance. BMJ.

[12] Viale P, Scudeller L, Pea F (2015). Implementation of a meningitis care bundle in the emergency room reduces mortality associated with acute bacterial meningitis. Ann Pharmacother.

[13] Backman R, Foy R, Diggle PJ (2018). A pragmatic cluster randomised controlled trial of a tailored intervention to improve the initial management of suspected encephalitis. PLoS One.

[14] Wall EC, Mukaka M, Denis B (2017). Goal directed therapy for suspected acute bacterial meningitis in adults and adolescents in sub-Saharan Africa. PLoS One.

[15] Mfinanga S, Kanyama C, Kouanfack C (2023). Reduction in mortality from HIV-related CNS infections in routine care in Africa (DREAMM): a before-and-after, implementation study. Lancet HIV.

[16] Allegranzi B, Aiken AM, Zeynep Kubilay N (2018). A multimodal infection control and patient safety intervention to reduce surgical site infections in Africa: a multicentre, before-after, cohort study. Lancet Infect Dis.

[17] Solomon T, Dung NM, Vaughn DW (2000). Neurological manifestations of dengue infection. Lancet.

[18] Harris PA, Taylor R, Minor BL (2019). The REDCap consortium: building an international community of software platform partners. J Biomed Inform.

[19] WHO (2012). Laboratory assessment tool.

[20] WHO (2019). Second WHO model list of essential in vitro diagnostics.

[21] Hoffmann TC, Glasziou PP, Boutron I (2014). Better reporting of interventions: template for intervention description and replication (TIDieR) checklist and guide. BMJ.

[22] Venkatesan A, Tunkel AR, Bloch KC (2013). Case definitions, diagnostic algorithms, and priorities in encephalitis: consensus statement of the International Encephalitis Consortium. Clin Infect Dis.

[23] Solomon T, Thao TT, Lewthwaite P (2008). A cohort study to assess the new WHO Japanese encephalitis surveillance standards. Bull World Health Organ.

[24] World Health Organization (2018). Meningococcus: vaccine preventable diseases surveillance standards.

[25] Granerod J, Cunningham R, Zuckerman M (2010). Causality in acute encephalitis: defining aetiologies. Epidemiol Infect.

[26] Singh B, Lant S, McGill F (2025). Defining causality in acute brain infections.

[27] Rabin R, de Charro F (2001). EQ-5D: a measure of health status from the EuroQol Group. Ann Med.

[28] Wille N, Badia X, Bonsel G (2010). Development of the EQ-5D-Y: a child-friendly version of the EQ-5D. Qual Life Res.

[29] Lewthwaite P, Begum A, Ooi MH (2010). Disability after encephalitis: development and validation of a new outcome score. Bull World Health Organ.

[30] Gale NK, Heath G, Cameron E, Rashid S, Redwood S (2013). Using the framework method for the analysis of qualitative data in multi-disciplinary health research. BMC Med Res Methodol.

[31] Bernal JL, Cummins S, Gasparrini A (2017). Interrupted time series regression for the evaluation of public health interventions: a tutorial. Int J Epidemiol.

[32] Granerod J, Ambrose HE, Davies NW (2010). Causes of encephalitis and differences in their clinical presentations in England: a multicentre, population-based prospective study. Lancet Infect Dis.

[33] Mallewa M, Vallely P, Faragher B (2013). Viral CNS infections in children from a malaria-endemic area of Malawi: a prospective cohort study. Lancet Glob Health.

[34] Ho Dang Trung N, Le Thi Phuong T, Wolbers M (2012). Aetiologies of central nervous system infection in Viet Nam: a prospective provincial hospital-based descriptive surveillance study. PLoS One.

[35] Hawley S, Ali MS, Berencsi K, Judge A, Prieto-Alhambra D (2019). Sample size and power considerations for ordinary least squares interrupted time series analysis: a simulation study. Clin Epidemiol.

[36] Lilford RJ, Chilton PJ, Hemming K, Girling AJ, Taylor CA, Barach P (2010). Evaluating policy and service interventions: framework to guide selection and interpretation of study end points. BMJ.

[37] McGill F, Griffiths MJ, Bonnett LJ (2018). Incidence, aetiology, and sequelae of viral meningitis in UK adults: a multicentre prospective observational cohort study. Lancet Infect Dis.

[38] Damodar T, Singh B, Prabhu N (2023). Association of scrub typhus in children with acute encephalitis syndrome and meningoencephalitis, southern India. Emerg Infect Dis.

[39] Alam AM, Gillespie CS, Goodall J (2022). Neurological manifestations of scrub typhus infection: a systematic review and meta-analysis of clinical features and case fatality. PLoS Negl Trop Dis.

[40] Griffiths MJ, McGill F, Solomon T (2018). Management of acute meningitis. Clin Med (Lond).

[41] Thwaites G, Fisher M, Hemingway C, Scott G, Solomon T, Innes J (2009). British Infection Society guidelines for the diagnosis and treatment of tuberculosis of the central nervous system in adults and children. J Infect.

[42] Ooi MH, Wong SC, Lewthwaite P, Cardosa MJ, Solomon T (2010). Clinical features, diagnosis, and management of enterovirus 71. Lancet Neurol.

[43] Miraclin AT, Singh B, Rupali P (2024). Central nervous system infections in the tropics. Curr Opin Infect Dis.

[44] Pommier JD, Gorman C, Crabol Y (2022). Childhood encephalitis in the Greater Mekong region (the Southeast Asia Encephalitis Project): a multicentre prospective study. Lancet Glob Health.

[45] Benjamin LA, Lewthwaite P, Vasanthapuram R (2011). Human parvovirus 4 as potential cause of encephalitis in children, India. Emerg Infect Dis.

[46] Granerod J, Huang Y, Davies NWS (2023). Global landscape of encephalitis: key priorities to reduce future disease burden. Clin Infect Dis.

[47] Lanciotti RS, Roehrig JT, Deubel V (1999). Origin of the West Nile virus responsible for an outbreak of encephalitis in the northeastern United States. Science.

[48] Chua KB, Goh KJ, Wong KT (1999). Fatal encephalitis due to Nipah virus among pig-farmers in Malaysia. Lancet.

[49] Solomon T, Ni H, Beasley DWC, Ekkelenkamp M, Cardosa MJ, Barrett ADT (2003). Origin and evolution of Japanese encephalitis virus in southeast Asia. J Virol.

[50] WHO (2023). Strengthening the global architecture for health emergency prevention, preparedness, response and resilience.

