# Supplementary material for: A multifaceted intervention to improve diagnosis and early management of hospitalised patients with suspected acute brain infections in Brazil, India, and Malawi: an international multicentre intervention study
Source: Lancet. Author manuscript; Available in PMC 2025 Oct 28. (PMC7618301; doi:10.1016/S0140-6736(25)00263-6)
Supplement: Supplementary Materials [file EMS209560-supplement-Supplementary_Materials.zip › 1-s2.0-S0140673625002636-mmc1.pdf]

THE LANCET

## Supplementary appendix 1

This appendix formed part of the original submission and has been peer reviewed. We post it as supplied by the authors.

Supplement to: Singh B, Lipunga GD, Thangavelu P, et al. A multifaceted intervention to improve diagnosis and early management of hospitalised patients with suspected acute brain infections in Brazil, India, and Malawi: an international multicentre intervention study. *Lancet* 2025; published online March 10. https://doi.org/10.1016/S0140-6736(25)00263-6.

# Supplementary Appendix 1: Additional Methods

**Table of contents**


1. SUPPLEMENTARY METHODS ........ 1
   *Table S1.1: Sampling criteria documentation table for journey observations in each hospital* ........ 5
   *Table S1.2: Covariates used in regression models for each outcome* ........ 5
2. **TOOL FOR VISUALISATION OF PATIENT JOURNEY – EXAMPLE** ........ 6
3. **PRIORITISATION TOOL FOR POTENTIAL *BRAIN INFECTIONS GLOBAL* STUDY INTERVENTION COMPONENTS** ........ 7
4. **DIAGNOSIS ASSESSMENT TOOL: OVERVIEW, AND SYNDROMIC DIAGNOSIS** ........ 10
5. **MICROBIOLOGICAL DIAGNOSIS ASSESSMENT TOOL** ........ 17
   *Table S1.3: Microbiological diagnosis principles for non-brain infection syndromes* ........ 17
6. **TOOL FOR ASSESSMENT OF APPROPRIATE ANTI-INFECTIVE THERAPY REGIMENS** ........ 18
   *Table S1.4: Features and principles of the therapy outcome assessment* ........ 19
   *Table S1.5: Brazil empirical therapy* ........ 21
   *Table S1.6: India empirical therapy* ........ 22
   *Table S1.7: Malawi empirical therapy* ........ 23
   *Table S1.8: Definitive anti-infective therapy regimens* ........ 24
7. **REFERENCES** ........ 27
8. BRAIN INFECTIONS GLOBAL INTERVENTION STUDY GROUP ........ 29
9. ETHICS COMMITTEE APPROVALS ........ 37


## 1. Supplementary Methods

### Intervention development

The intervention followed an overall structure based on the WHO health system building blocks.[1] The intervention design process followed the Medical Research Council's guidance on complex interventions, following the stepwise 6-SQUID approach.[2,3] Intervention design was overseen across centres by a multidisciplinary core working group, with members from each centre, to ensure alignment of approach between centres. This group met frequently with the patient and public involvement panel to ensure relevance to public and patient priorities. Individual intervention components were proposed based on integrating results from the journey observations, laboratory assessments and insights from quantitative data from patients recruited pre-intervention. Investigators at each centre worked with clinicians, laboratory leads and policymakers at each hospital and in the wider health system to draft the intervention using a prioritisation tool, based on a matrix which guided systematic consideration of the likely impact, cost, feasibility and sustainability of each intervention component (appendix 1 pp 7-9). We used the COM-B behaviour change wheel as a framework to consider potential impact of intervention components on capability, opportunity and motivation, and their ability to serve the key functions of enablement and training to improve service provision as the primary policy goal, in a feasible and sustainable way.[4] The intervention had to be affordable for the study budget at each centre, but also

buy-in for potential continuation of the intervention beyond the study was obtained prospectively from the study's policy working group, which included policymakers from each centre/country (including some at state and national level) and policy-specialist investigators.

**Intervention implementation**

Clinical algorithms were shared electronically with all relevant clinicians, and put up as posters on walls in key clinical areas, primarily emergency departments and acute care wards. Lumbar puncture packs were set up, or existing packs were modified, and placed with guidance sheets in clinical areas alongside the clinical algorithms. Clinical training was delivered in person to key groups of clinicians by experienced clinical investigators, which included orientation to the new or updated algorithms and lumbar puncture packs, and, at some hospitals, training in performing lumbar punctures. Various e-learning modules on brain infections were made available to clinical staff on the Brain Infections Global website. Clinicians leading the training were available for queries relating to the algorithms and lumbar puncture packs, and management of individual patients. All of the clinical algorithms and lumbar puncture pack guidance sheets are available on the Brain Infections Global website (https://braininfectionsglobal.tghn.org/resources/brain-infections-global-tools/).

Laboratory training was delivered in person to groups of technical staff from hospital laboratories by experienced specialists in tests and techniques relevant to each hospital (ranging from microscopy for cell counts and bacteria, to serological and PCR tests), using standard operating procedures (SOPs). Training sessions ranged from half a day to two days in duration; in some centres, there were two sessions: one at the central laboratory and a follow-up session in the hospital laboratory. The specialists were available for troubleshooting and reinforcement of techniques; central laboratories provided reference testing for evaluating unexpected results. Laboratory algorithms were placed in laboratories (and in some cases were formatted and incorporated as SOPs). Kits, reagents and consumables were provided by the study teams, using locally accessible suppliers; some hospitals placed the orders themselves. Low-cost equipment (centrifuges, refrigerators/freezers) was bought for some laboratories by the study teams; some hospitals bought their own equipment thanks to buy-in from hospital management. Logistical solutions were set up for transport of CSF and/or blood specimens to another study hospital or central laboratory if tests could not be set up at a given hospital. Pathogen tests in the stepwise diagnostic testing panels for each country are provided in appendix 2 (pp 5—7) and on the Brain Infections Global website (https://braininfectionsglobal.tghn.org/resources/brain-infections-global-tools/). Clinical interpretation was provided for positive and equivocal results. Some pathogen detection tests could not be set up: these are indicated in footnotes in the figure. The majority of tests that could not be implemented were in 'step 2' of the panel, and had therefore not been prioritised in terms of commonality or treatability of the pathogens.

During implementation of the intervention, at least one member of the group (BS, +/- TS) was physically present in each centre to facilitate implementation and align approaches across centres, while acknowledging the need for this approach to be individualised based on local contextual factors. Study teams in each centre were on hand to support clinicians and laboratory staff with implementation of the intervention after initial orientation and training, and re-orientate staff as needed. Teams were multi-disciplinary, including those with clinical (multiple specialties), laboratory and social and behavioural science expertise. One member of the group (BS) visited each site formally part-way through the intervention to ensure the intervention was in place and functioning as intended, and to obtain informal feedback from end-users, which then informed modification of the intervention by study teams in collaboration with hospital stakeholders, to improve adherence and/or impact.

**Outcome assessment and measurement**

Syndromic diagnosis criteria defined confirmation of a brain infection syndrome through one or more of the following: 1) demonstration of raised cerebrospinal leukocyte count; 2) typical imaging features; 3) one of a few specific pathogen tests (e.g. for rabies virus or Cryptococcus) that would confirm a brain infection in the absence of criteria 1 and 2; or 4) histopathological evidence of an infectious process in brain or meningeal tissue. Specific tests could also be used to provide evidence of an alternative, non-brain infection, diagnosis. Criteria for microbiological diagnosis were developed through extension of existing criteria for encephalitis causation in the UK by adding pathogens relevant to tropical and low-resource settings, and modifying the criteria for use for patients with suspected meningitis, brain abscess and other brain infection and

non-brain infection syndromes.[5,6] This involved judgement of whether test results provided evidence of a confirmed, probable or possible causative pathogen, according to the likelihood that a pathogen was responsible for the presenting illness, and the level of evidence of its presence in central nervous system or elsewhere. The tools for assessing diagnosis outcomes, including the approach to microbiological diagnosis for patients not meeting a definition for a brain infection syndrome are provided in this appendix (appendix 1 pp 10–17), while the case definitions for microbiological diagnosis are described elsewhere.[6] For both primary outcomes, assessment followed a stringent process to minimise bias: multiple assessors were trained, and went through an initial process of calibration, followed by regular meetings of all assessors to maintain this. Each patient was assessed by two independent investigators blinded from each other's assessment, at least one of whom was not based at that study site, and, where possible, was not involved in design or implementation of the intervention. The assessments were compared by a statistician, blinded to the study. If there was a disagreement on either outcome, then this was resolved through discussion between the assessors. In cases of ongoing disagreement, or where the case was deemed to be difficult, a third senior assessor was asked to make a final judgement, who was blinded to the phase in which the participant was recruited.

Secondary outcomes included relevant process measures and key patient- and health system-oriented outcomes. Process outcomes comprised the proportion of participants receiving a lumbar puncture procedure to obtain CSF, and the timing of this from presentation to hospital; and the proportion of those having CSF taken undergoing appropriate investigations, defined as all of the following, regardless of syndromic or microbiological diagnosis: white blood cell count, protein concentration, glucose concentration and microscopy and culture for bacteria on CSF, and a blood glucose concentration paired with the CSF (appropriate CSF tests *without* paired blood glucose was added as an outcome post hoc, after it was realised that documentation of the paired blood glucose was not standardised and often difficult to locate). All-cause mortality was measured at 30 days from presentation, or at latest follow-up; length of stay in hospital was measured in days; time to appropriate empirical anti-infective therapy and appropriate anti-infective definitive therapy were measured from presentation to hospital in days, and using pre-defined criteria and two independent assessors as for the diagnosis outcomes (appendix 1 pp 18–26); quality of life was measured at discharge from hospital and at 30 days after presentation using the EQ-5D-Y for children aged 8 to 15 years and EQ-5D-3L for participants aged 16 years and over; and neurological function was measured at discharge from hospital and at 30 days after presentation using the Liverpool Outcome Score, which was designed originally for children with encephalitis in low-resource settings, and has adult and child versions. All questionnaires were administered in a language in which the participant or their representative was fluent.

**Analysis**

Analysis of data collected in the journey observations (appendix 1 p 6) involved initial qualitative analysis of ethnographic commentary by at least two investigators using a modified abbreviated framework approach. A matrix specially made in Microsoft Excel allowed themes relating to facilitators and challenges in patient diagnosis and management to be captured for individual journeys, and then across journeys in each hospital, using a combined deductive (pre-defined potential themes) and inductive (new emerging themes) approach. In parallel, descriptive statistics were used to summarise quantitative journey data, such as time to various key steps in the process, and again a team at each centre captured insights from these. Next, these were compared between hospitals in each centre, and across centres, among the 'current practice' social science team, to enable sharing of themes that may be relevant in different settings. These were then shared with investigators and hospital stakeholders, and the core intervention working group, to inform design of the intervention.

The primary analysis for the primary outcomes was planned to include a before-versus-after comparison, as well as an interrupted time series analysis, which assumes a potential increasing trend or predictable variability (due to seasonal variation) in the proportion receiving a diagnosis over time pre-intervention. This involves analysis for two elements: a step (also referred to as level) change, representing an immediate step up or down in the proportion achieving a diagnosis; and a slope change, signifying a more gradual and sustained increase or decrease in the gradient over time.[7] While some aspects of the multifaceted hospital-wide intervention may be expected to result in an immediate increase in diagnosis (e.g. new laboratory tests for specific pathogens), and therefore be modelled by a step change, others may have a more slow and sustained effect, such as training of clinical and laboratory staff, with the slope more likely to be affected. Therefore both step and slope changes were analysed. Two multi-level multivariable logistic regression models were developed, with individual patients as the unit of analysis, for the overall pooled dataset (i.e. across all centres), and two for

each study centre. Both had achievement of a diagnosis as the outcome and presence of the intervention as a predictor variable; in the second model the interaction between the intervention and time (in months) was added as another predictor variable to represent change in the slope before vs. after the intervention, in which the presence-of-intervention variable represented a step-change in diagnosis at implementation of the intervention. While it was anticipated that some variables would be matched in participants enrolled before vs. after implementation of the intervention, due to the study sites remaining the same, specific covariates hypothesised to predict likelihood of a diagnosis being made were included in the multi-level models: age group (as categorical groups of <1 year; 1-5 years; 6-15 years; 16-59 years and ≥60 years); presence vs. absence of signs of meningeal irritation; duration of symptoms prior to presentation to the study hospital (< 7 days vs. ≥ 7 days); and whether the participant presented in a rainy month (or in a month immediately after a rainy month; i.e. rainy vs. not-rainy), based on observed meteorological records. Additionally, as the pathogens, participants and systems are quite different in each centre, and to a degree at individual hospitals in each centre, the likelihood of achieving a diagnosis was assumed to vary at baseline, so in the overall model centre was modelled as a random-effects variable, whereas in the centre-level models hospital was used as this random-effects variable. Months with very low denominators were removed from the interrupted time series (i.e. step and slope) models and graphs. For the overall analyses, the first month was removed; for Bangalore, the first two months and the last two months pre-intervention were removed; for Vellore and Brazil, the first two months were removed. As a sensitivity analysis for the overall dataset, centre was included as a fixed-effect variable with the other potential confounders, and hospital was included as a random-effect variable. As no significant autocorrelation was observed (similarity of measurements close to each other in time, or at recurring intervals), the analysis did not adjust for this.

For secondary outcomes that were proportions, we made models similar to the first set of models described for the primary outcomes, with resulting aORs representing odds of improvement in each outcome associated with the intervention (except mortality, for which this was the odds of increased mortality). These were adjusted for covariates other than centre/hospital (included in all models: centre as fixed-effect and hospital as random-effect), which were pre-defined individually based on what was decided to be relevant to each outcome. The second model assessing step and slope change was only run if there was a significant difference in the proportion achieving the outcome using the initial before versus after model. For time-to-event outcomes, Kaplan-Meier curves were made, followed by multivariable Cox proportional-hazards regression, using pre-defined covariates relevant to each outcome, with resulting univariate and adjusted hazard ratios (aHR) and 95% CI. For length of stay in hospital, in addition to this, a competing risks regression approach was used to account for death as a competing risk for discharge alive.[8] EQ-5D overall health values were calculated from scores on each of the five domains of the questionnaire using the widely-used UK adult (EQ-5D-3L) value set for all data, including adults and children, using the *eq5d* package in R, presenting results as a mean difference and 95% CI. While there are limitations to this approach, including the use of an old value set which is adult-specific and not made for the countries in which participants were recruited, value sets were not available for each country, and this approach has been taken by other studies.[9] Mean scores in the pre-intervention vs. post-intervention groups were compared using a two-sample t test. The lowest score in any domain of the Liverpool Outcome Score provides an ordinal scale from 1 (death) to 5 (full recovery & normal neurological examination), which was then analysed in a before-vs.-after comparison using ordinal logistic regression, with covariates as listed in Table S1.2 (appendix 1 p 5), with odds of higher score post-intervention reported as aOR and 95% CI. As for primary outcomes, the secondary outcomes were analysed for the overall across-centre dataset and for individual centres.

Costs of the intervention were documented in real-time during its design and implementation, to enable calculation of the incremental cost of the intervention to the health system. While actual amounts of money spent by each centre were calculated, where an existing cost to the system was available for a specific test or service, this was taken as a more reliable representation of the likely cost to the system of performing more such tests, even if during the intervention some of these were done by the study team. Total cost of the intervention per centre was then averaged across the post-intervention participants.

## Table S1.1: Sampling criteria documentation table for journey observations in each hospital

| **Characteristic**<br>***Categories*** | **Patient 1** | **Patient 2** | **Patient 3** | **Patient 4** | **Patient 5** | **Patient 6** | **Patient 7** | **Patient 8** | **Patient 9** | **Patient 10** | **Patient 11** | **Patient 12** |
|---|---|---|---|---|---|---|---|---|---|---|---|---|
| ***2 or 3 categories per characteristic*** | ***Enter which category each patient is classified into for each characteristic*** | | | | | | | | | | | |
| **Age**<br>*1 = Child <12m; 2 = Child >12m; 3 = Adult* | | | | | | | | | | | | |
| **HIV status**<br>*1 = Positive; 2 = Negative/Unknown* | | | | | | | | | | | | |
| **Presence of encephalopathy (altered mental status, seizure or focal neurological sign)**<br>*1 = Present; 2 = Absent* | | | | | | | | | | | | |
| **Duration of symptoms prior to presentation**<br>*1 = <5 days; 2 = ≥5 days* | | | | | | | | | | | | |
| **Time of day of presentation**<br>*1 = day; 2 = night* | | | | | | | | | | | | |
| **Received antibiotics before presentation**<br>*1 = Yes; 2 = No* | | | | | | | | | | | | |
| **Day of week of presentation**<br>*1 = Weekday; 2 = Weekend* | | | | | | | | | | | | |
| **Gender**<br>*1 = Female; 2 = Male* | | | | | | | | | | | | |

## Table S1.2: Covariates used in regression models for each outcome

| **Outcome** | **Age group** | **Signs of meningeal irritation** | **Duration of symptoms** | **Presented in a rainy/post-rainy month** | **Low blood platelet count[1]** | **Uncontrolled seizures[2]** | **Reduced conscious level[3]** | **Previous anti-infective treatment[4]** |
|---|---|---|---|---|---|---|---|---|
| **Syndromic diagnosis** | X | X | X | X | | | | |
| **Microbiological diagnosis** | X | X | X | X | | | | |
| **Lumbar puncture performed** | X | X | X | X | | | | |
| **Time to lumbar puncture** | X | X | X | | X | X | | |
| **Basic appropriate CSF tests performed** | X | X | | | | | | |
| **Mortality** | X | | | | | | X | |
| **Time to discharge from hospital** | X | | | | | | X | |
| **Time to appropriate empirical therapy** | X | X | X | X | | | | X |
| **Time to appropriate definitive therapy** | X | X | X | X | | | | X |
| **Liverpool Outcome Score** | X | | | | | | | |

In addition to the above, all analyses were performed with centre as a covariate for across-centre analyses on the overall dataset, and hospital was included as a covariate in all centre-level models. All models were built to be parsimonious, i.e. to prioritise key covariates without including too many.

[1]Less than $100x10^9$/L on the first result after presentation

[2]Five or more prior to, or in status epilepticus at presentation

[3]Recorded examination finding of reduced conscious level or coma, Glasgow Coma Scale score of less than 13, or Blantyre Coma Score of less than 4

[4]Received during the current illness, but prior to presentation to the study hospital

## 2. Tool for visualisation of patient journey – example

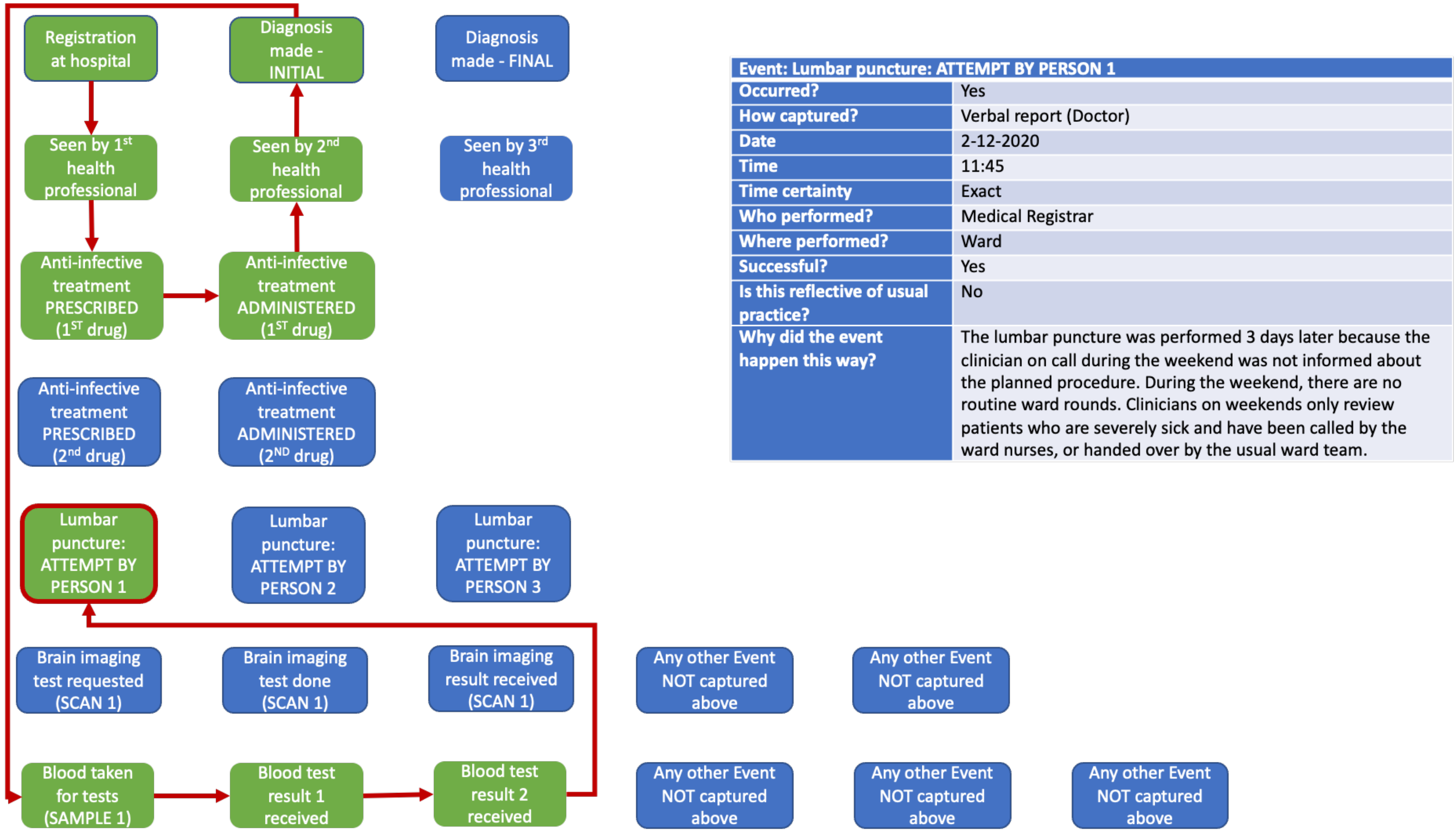


| Event: Lumbar puncture: ATTEMPT BY PERSON 1 | |
|---|---|
| Occurred? | Yes |
| How captured? | Verbal report (Doctor) |
| Date | 2-12-2020 |
| Time | 11:45 |
| Time certainty | Exact |
| Who performed? | Medical Registrar |
| Where performed? | Ward |
| Successful? | Yes |
| Is this reflective of usual practice? | No |
| Why did the event happen this way? | The lumbar puncture was performed 3 days later because the clinician on call during the weekend was not informed about the planned procedure. During the weekend, there are no routine ward rounds. Clinicians on weekends only review patients who are severely sick and have been called by the ward nurses, or handed over by the usual ward team. |

This is one slide taken from an example presentation, which was made for each patient journey observed. The box in the upper-right of the figure contains data recorded about each event in the online data collection tool. In this example, the event is the first attempt at a lumbar puncture, which is the green box with a red outline, to which the latest red arrow points. Green boxes represent events that have occurred to this point in the journey; red outline is used for the current event; and red arrows show the order of events, to this point in the journey.

## 3. Prioritisation Tool for Potential *Brain Infections Global* Study Intervention Components

HOSPITAL: ____________________ UPDATED ON DATE: ________________

*A new document should be completed for each hospital within each centre. Any opportunities for providing the intervention component at centre-level, or for more than one hospital within a centre, should be indicated in the Notes column below.*

| **Potential Component** | **Mapping to intervention structure (see below)** | | **Impact** (1-10) | **Cost** (1-5; inverse) | **Feasibility** (1-5) | **Sustain-ability** (1-5) | **TOTAL** (4-25) | **Notes** |
|---|---|---|---|---|---|---|---|---|
| | **Domain** | **Component** | | | | | | |
| *EXAMPLE: Lumbar puncture pack* | *Processes* | *LP pack* | *10* | *3* | *4* | *4* | *20* | *Hospital already has one. Improvement in this could aid sample collection & transport, with all consumables in one place, and guidance on how much to collect & how to send to each lab.* |
| | | | | | | | | |
| | | | | | | | | |
| | | | | | | | | |
| | | | | | | | | |
| | | | | | | | | |
| | | | | | | | | |
| | | | | | | | | |
| | | | | | | | | |
| | | | | | | | | |
| | | | | | | | | |
| | | | | | | | | |
| | | | | | | | | |
| | | | | | | | | |
| | | | | | | | | |
| | | | | | | | | |
| | | | | | | | | |

**Overall intervention structure**

The domains (columns with blue headers) contain various components (golden boxes).
Not all components will be used in each hospital, and their content and delivery will vary between hospitals.

| HUMAN RESOURCES | ACCESS & PROCUREMENT | PROCESSES |
|---|---|---|
| Clinical training | Diagnostic kits | Clinical algorithms |
| Lab training | Consumables | Lab algorithms |
| HR management | Equipment | Lumbar puncture pack |
| *Each component tailored to hospitals' needs & capacity* | Medicines | Logistics |

HR = human resources

**Definitions & scoring**

<u>Impact</u>
*1 = least impact; 10 = greatest impact*
This is a judgement of the potential impact of the intervention component on, in order of priority:

- Score 8-10 if there is likely to be substantial impact on <u>whether</u> a diagnosis is made at all
- Score 5-7 if the impact will focus on <u>how quickly</u> a diagnosis is made
- Score 1-4 if the impact is on other aspects of care

This characteristic has the highest priority, and thus is scored out of 10, rather than 5.

<u>Cost</u>
*1 = **<u>highest</u>** cost; 5 = **<u>lowest</u>** cost*
This may not be easy to calculate, but the investigators should record this in terms relative to other potential intervention components, acknowledging the overall funds available to pay for the intervention in each centre:

- Score 4-5 if:

- total cost is <20% of the total available for the intervention
  and
- <20% of the costs for an average patient's admission

- Score 2-3 if one of the above conditions is met
- Score 1 if neither of the above conditions are met

Feasibility
*1 = least feasibility; 5 = greatest feasibility*
This is the ease with which the intervention component can be developed (with input from hospital stakeholders) and implemented, but is separate from its financial cost.

- Score 4-5 if:
  - there is evidence of this or a similar intervention working in another setting
    and
  - it is likely that there will be easy buy-in from all relevant stakeholders
- Score 2-3 if one of the above conditions is met
- Score 1 if neither of the above conditions are met

Sustainability
*1 = least sustainability; 5 = greatest sustainability*
This is the perceived likelihood of the intervention component remaining in place once the study is complete.

- Score 4-5 if the intervention is likely to be easy to sustain in the hospital system as it is:
  - likely to quickly become part of standard practice
    and
  - low-cost enough to not require ongoing external funding
- Score 2-3 if one of the above conditions is met
- Score 1 if neither of the above conditions are met

## 4. Diagnosis assessment tool: overview, and syndromic diagnosis

**DIAGNOSIS ACHIEVEMENT ASSESSMENT: OVERVIEW**

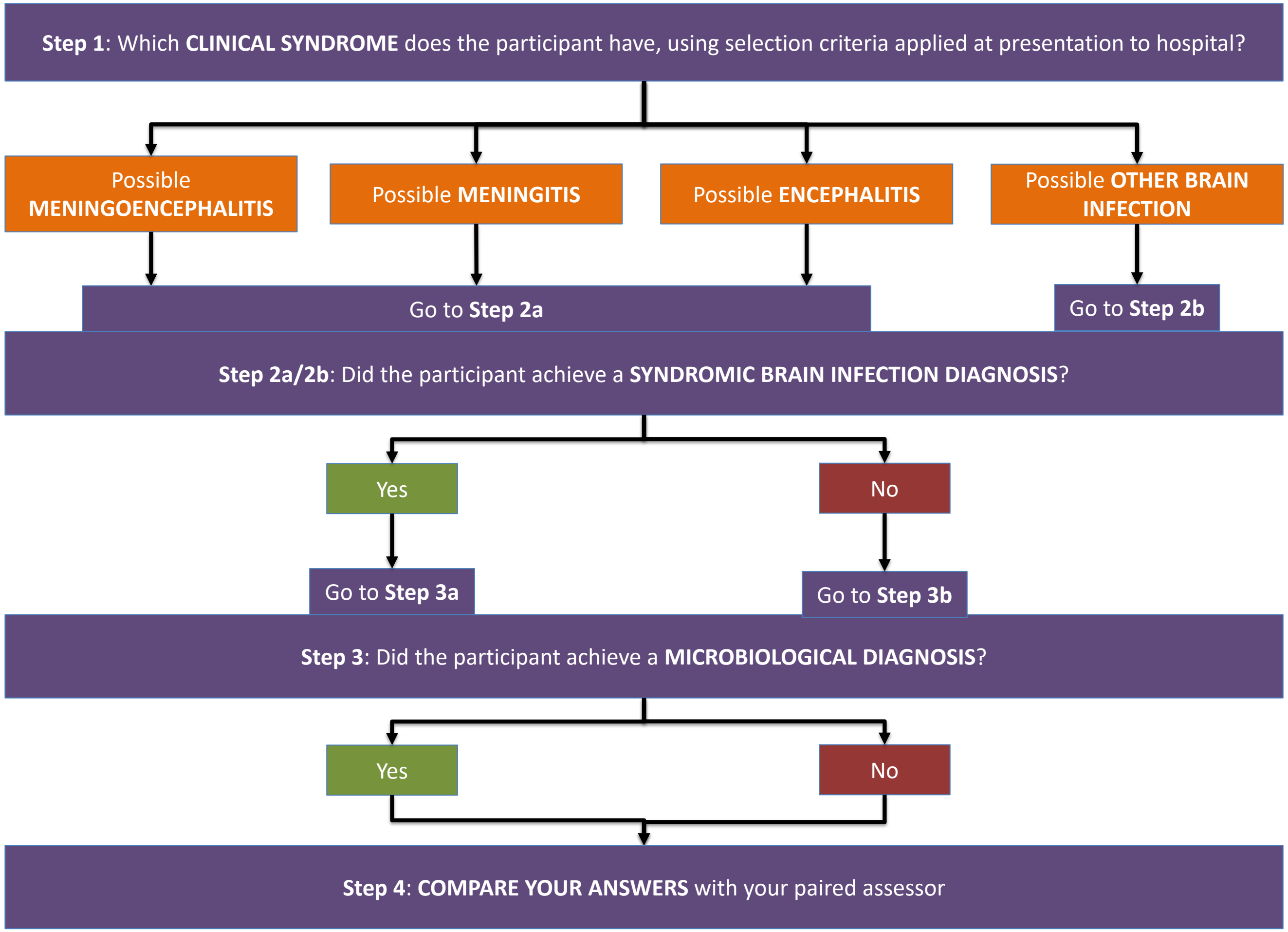

## DIAGNOSIS ACHIEVEMENT ASSESSMENT: STEP 1

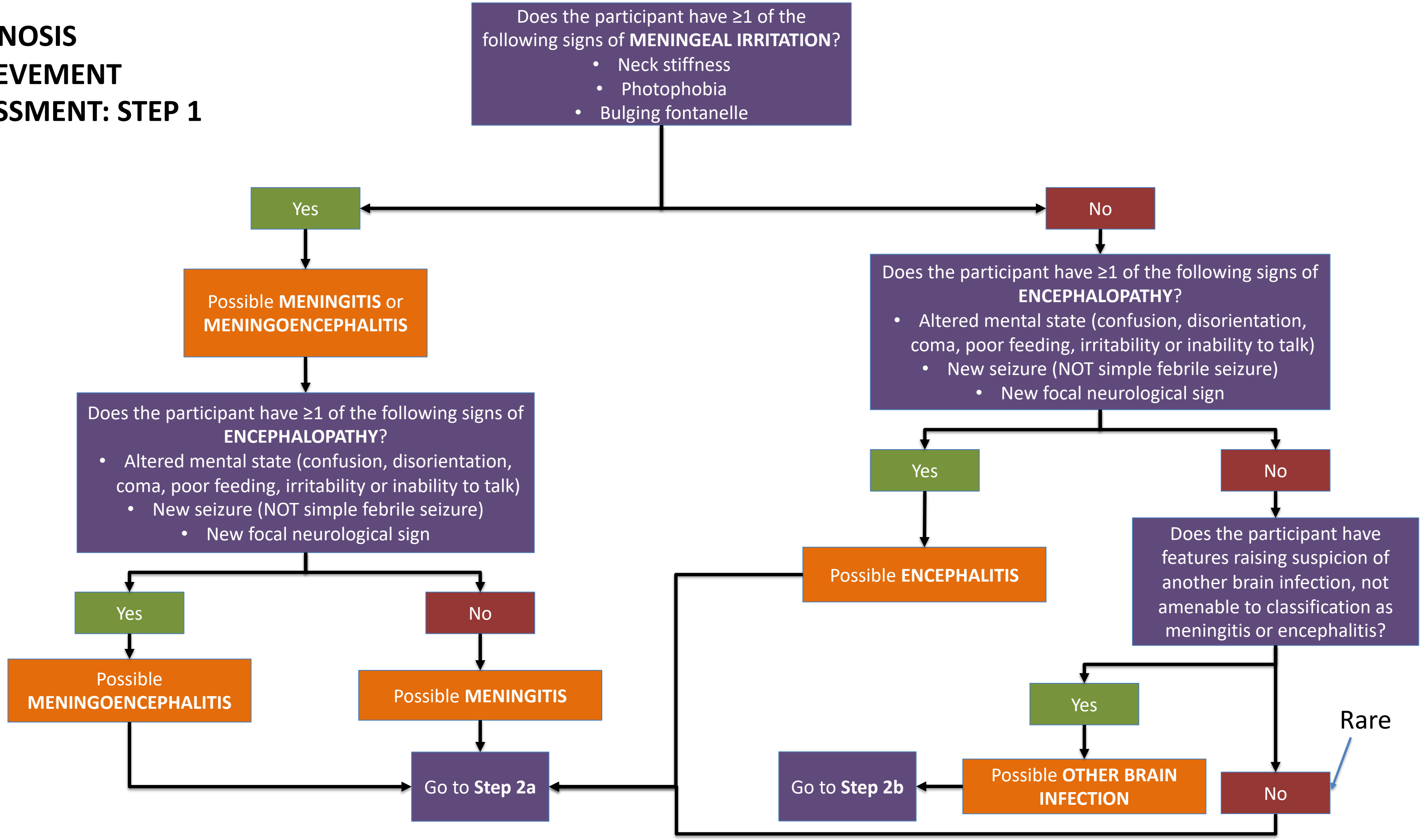

# DIAGNOSIS ACHIEVEMENT ASSESSMENT: STEP 2a

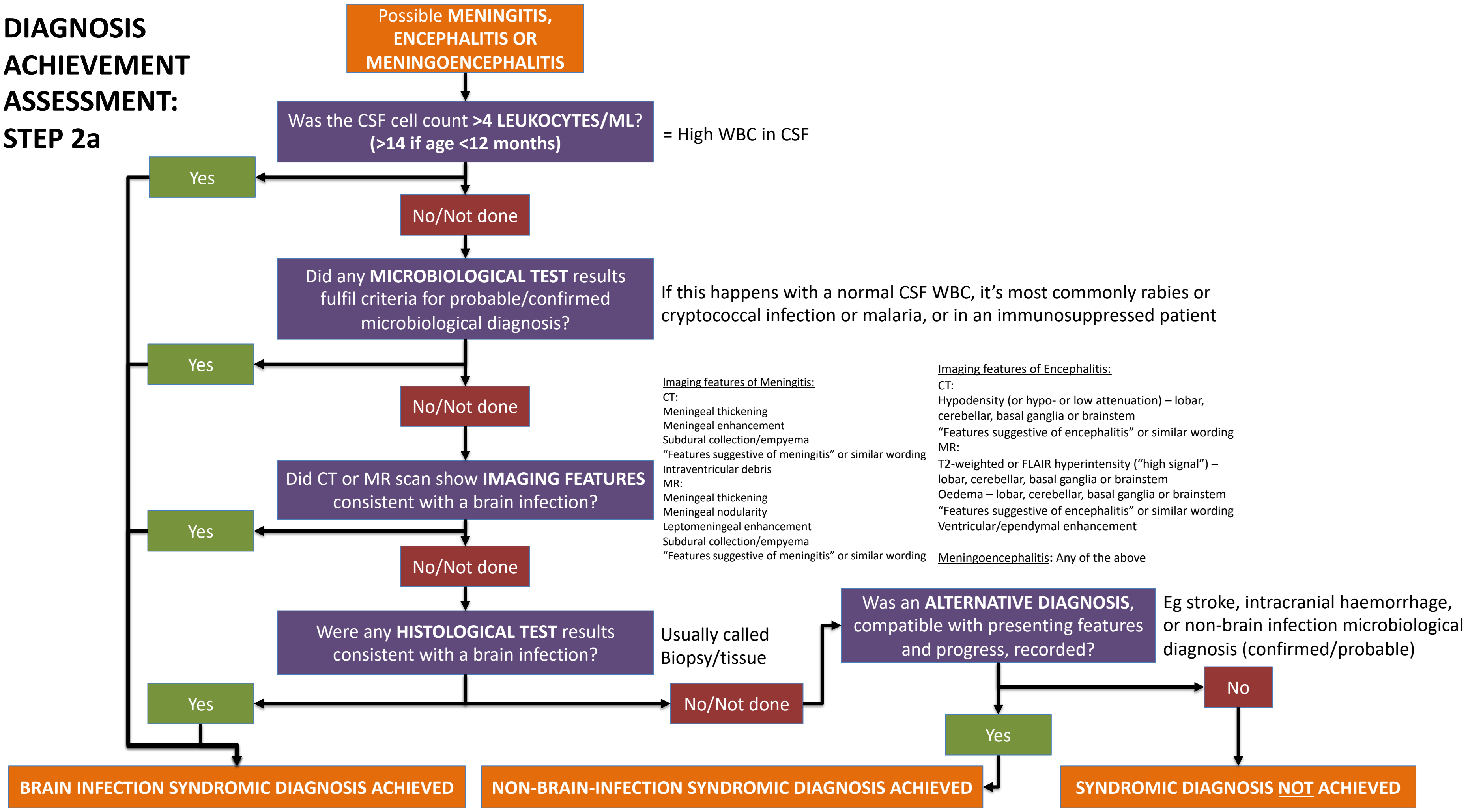

## DIAGNOSIS ACHIEVEMENT ASSESSMENT: STEP 2b

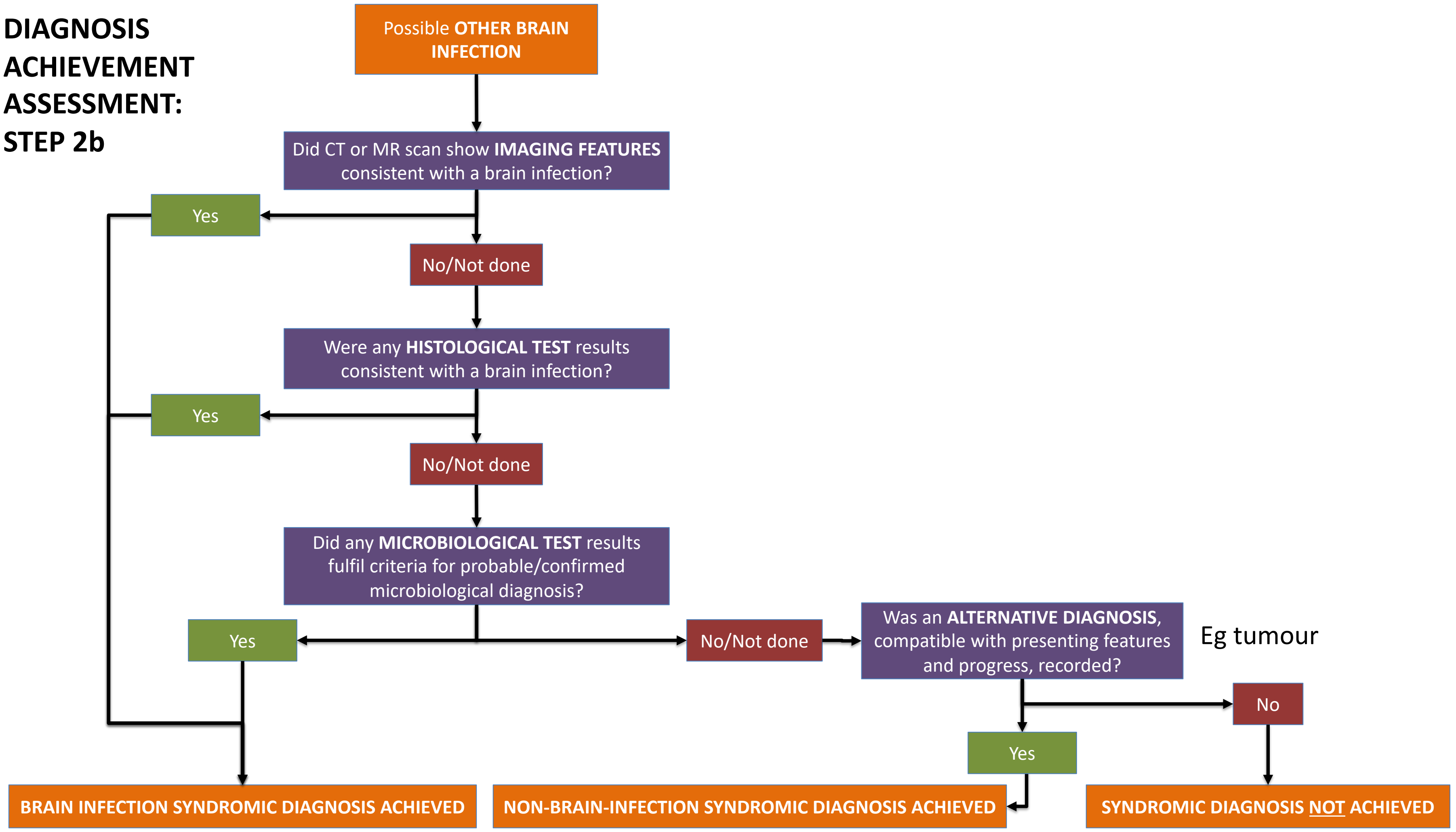

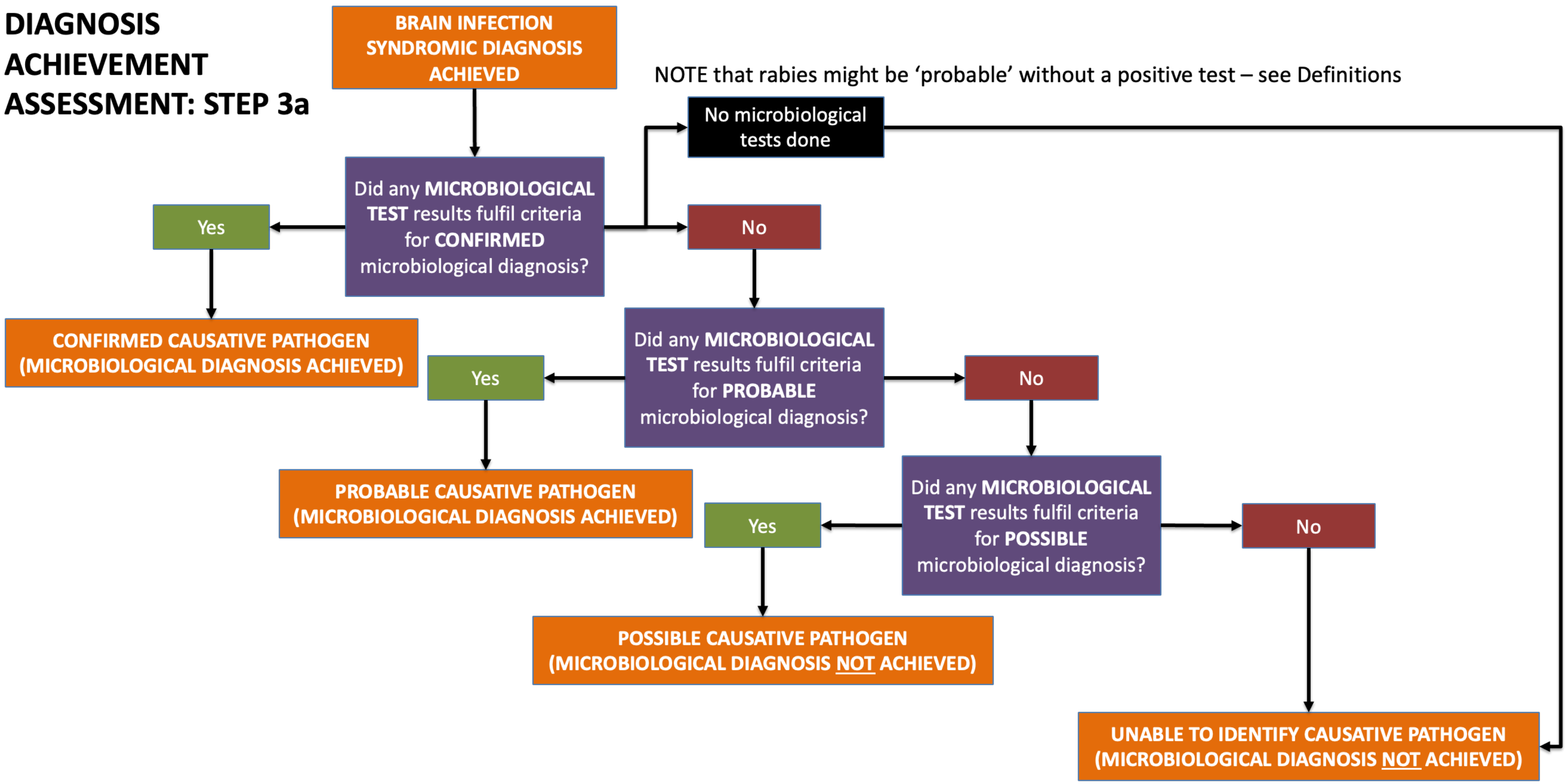
DIAGNOSIS ACHIEVEMENT ASSESSMENT: STEP 3a
BRAIN INFECTION SYNDROMIC DIAGNOSIS ACHIEVED
NOTE that rabies might be 'probable' without a positive test – see Definitions
No microbiological tests done
Did any MICROBIOLOGICAL TEST results fulfil criteria for CONFIRMED microbiological diagnosis?
Yes
No
CONFIRMED CAUSATIVE PATHOGEN (MICROBIOLOGICAL DIAGNOSIS ACHIEVED)
Did any MICROBIOLOGICAL TEST results fulfil criteria for PROBABLE microbiological diagnosis?
Yes
No
PROBABLE CAUSATIVE PATHOGEN (MICROBIOLOGICAL DIAGNOSIS ACHIEVED)
Did any MICROBIOLOGICAL TEST results fulfil criteria for POSSIBLE microbiological diagnosis?
Yes
No
POSSIBLE CAUSATIVE PATHOGEN (MICROBIOLOGICAL DIAGNOSIS NOT ACHIEVED)
UNABLE TO IDENTIFY CAUSATIVE PATHOGEN (MICROBIOLOGICAL DIAGNOSIS NOT ACHIEVED)

**DIAGNOSIS ACHIEVEMENT ASSESSMENT: STEP 3b**

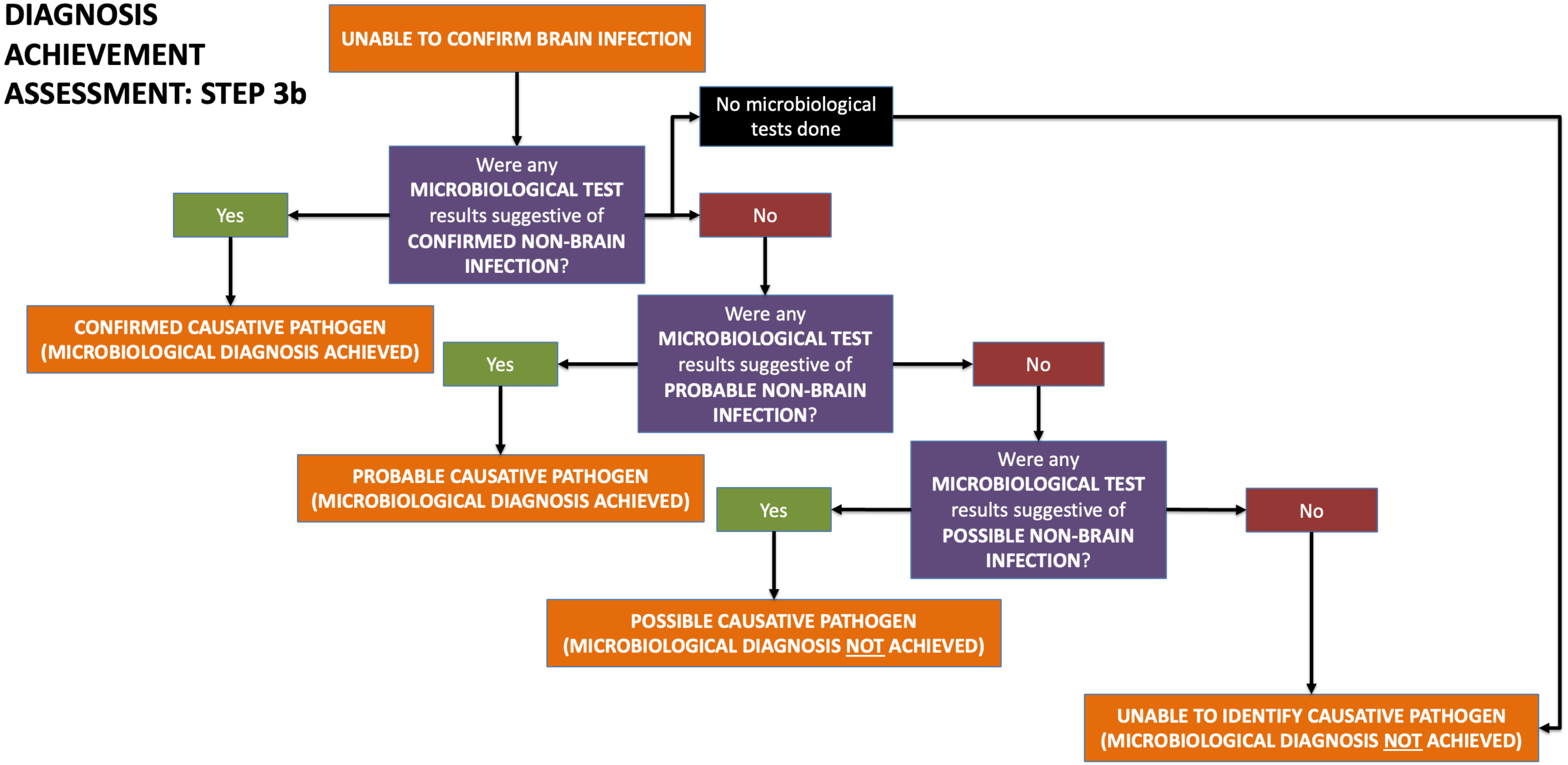

**DIAGNOSIS ACHIEVEMENT ASSESSMENT: STEP 4**

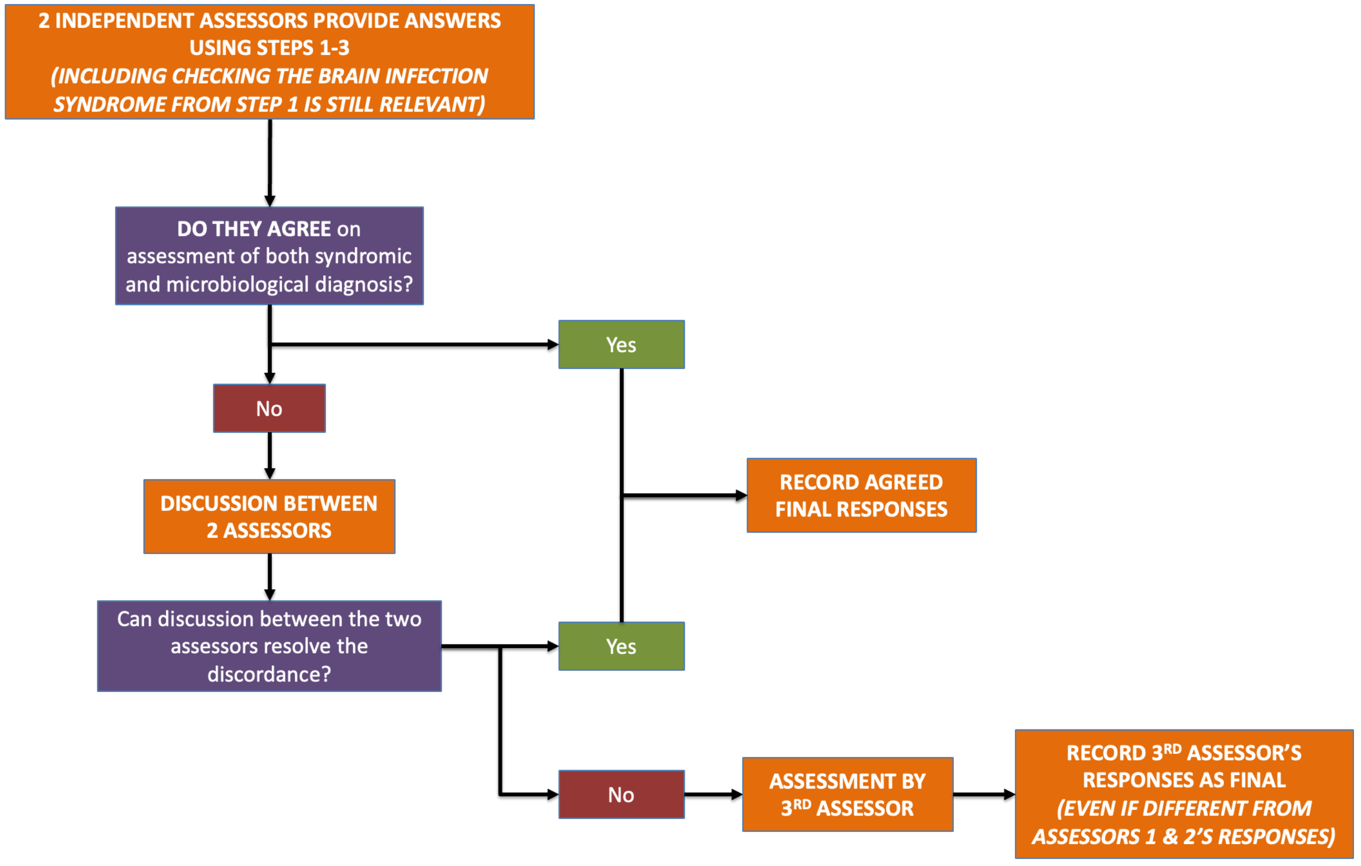

## 5. Microbiological diagnosis assessment tool

*For brain infection syndromes, case definitions for microbiological diagnosis are described in a separate paper.*[6]

### Table S1.3: Microbiological diagnosis principles for non-brain infection syndromes

| Diagnosis Outcome Label | Principles |
|---|---|
| **Confirmed** | Isolation by **culture** or detection of **nucleic acid** of a pathogen from a **sterile site** (blood, pleural, pericardial, synovial fluid), that is usually considered responsible for the presenting illness, and would usually be identified at that site.<br>OR<br>Evidence of seroconversion from IgG-negative to IgG-positive, or a 4-fold rise in IgG or neutralising antibody titres, in serum, against a specific pathogen, over a time frame relevant to that pathogen and the presenting illness. |
| **Probable** | Isolation by **culture** or detection of **nucleic acid** of a single (or predominating in a mixed result) pathogen from a **nonsterile site relevant to the presenting illness** (such as urine, stool, abscess pus, sputum), that is usually considered responsible for the illness.<br>OR<br>Detection of **antibodies in serum**, or other relevant sample, of **type** (IgM/IgG/total) appropriate to **time since symptom onset**, against a pathogen that is usually considered responsible for the presenting illness, either in the absence of an alternative cause, or with negative antibodies against similar pathogens, depending on the likelihood of cross-reactivity of antibodies.<br>OR<br>Detection of **antigen** of a pathogen in a **sample relevant to the illness** (such as serum, urine, stool, abscess pus, sputum), that is usually considered responsible for the presenting illness. |
| **Possible** | Isolation by **culture** or detection of **nucleic acid** of **mixed pathogens** from a **nonsterile site relevant to the presenting illness** (such as urine, stool, abscess pus, sputum), that would plausibly be responsible for the presenting illness.<br>OR<br>Isolation by **culture** or detection of **nucleic acid** of **a single pathogen** from a **nonsterile site <u>NOT</u> relevant to the presenting illness** (such as urine, stool, abscess pus, sputum), that is usually considered responsible for the presenting illness.<br>OR<br>Isolation by **culture** or detection of **nucleic acid** of **a single organism** from a **nonsterile site relevant to the presenting illness** (such as urine, stool, abscess pus, sputum), that is **<u>NOT</u> usually considered responsible** for the presenting illness.<br>OR<br>Detection of **antibodies in serum**, of **type** (IgM/IgG/total) **<u>NOT</u>** appropriate to **time since symptom onset**, against a pathogen that is usually considered responsible for the presenting illness.<br>OR<br>Detection of **antibodies in serum**, of **type** (IgM/IgG/total) **appropriate** to **time since symptom onset**, against an organism that is **<u>NOT</u> usually considered** responsible for the presenting illness.<br>OR<br>Detection of **antigen** of a pathogen in a **sample <u>NOT</u> relevant to the presenting illness**, that is usually considered responsible for the presenting illness.<br>OR<br>Detection of **antigen** of a pathogen in a **sample relevant to the presenting illness**, that is **<u>NOT</u> usually considered** responsible for the presenting illness. |

## 6. Tool for assessment of appropriate anti-infective therapy regimens

### Background & principles for using this tool

The following secondary outcomes of the *Brain Infections Global* study relate to whether participants received appropriate therapy for suspected acute brain infection:

- Receipt and time to appropriate **empirical** anti-infective therapy
- Receipt and time to appropriate **definitive** anti-infective therapy

These outcomes and their assessment are summarised in Table S1.4 (p 41).

Various guidelines, handbooks, position papers and consensus statements were consulted to inform the criteria within this tool.[10–22] However, due to their nature, there is some subjectivity in the judgement of whether these outcomes have been achieved for a given participant that meets the study's generic eligibility criteria, due to the clinical situation. This is especially so for the empirical therapy assessment. For example, a previously healthy adult presenting with a two-day history of abrupt onset suspected meningitis might justifiably be treated quite differently from an adult with newly diagnosed advanced HIV and 25 days of headache and neck stiffness. Therefore, while this protocol should cover most situations, sometimes it will be overridden.

There are two mechanisms built into the process to account for potential subjectivity, as with the diagnosis outcome assessment:

1. Two independent assessors will apply the protocol to each participant, blinded to each other's assessment.
2. If there is a difference in the assessments by assessor 1 and assessor 2, and consensus cannot be achieved through initial discussion, a third assessor will be asked to arbitrate, whose assessment will be taken as final.

## Table S1.4: Features and principles of the therapy outcome assessment

<table>
<tr><th></th><th>Empirical anti-infective therapy</th><th>Definitive anti-infective therapy</th></tr>
<tr><td>Definition</td><td>Initial therapy to cover various treatable pathogens commonly responsible for the presenting illness.</td><td>Specific therapy for a probable or confirmed pathogen causing the presenting illness.<br>Also known as directed therapy.</td></tr>
<tr><td>Which participants should be assessed?</td><td>All, regardless of what their final diagnosis is, according to clinicians or the study.</td><td>Only those with at least one confirmed or probable causative pathogen.<br>This can be for a brain infection, or a non-brain infection.</td></tr>
<tr><td>When is this therapy prescribed?</td><td>Usually before pathogen-specific results are available. Often it is the first anti-infective therapy received by the patient, though this may not be the case.</td><td>Usually when investigations identify a probable or confirmed pathogen responsible for the presenting illness.</td></tr>
<tr><td>Assessment steps</td><td>• A Yes/No/Not applicable answer, and a Date if Yes is selected, are required.<br>• Refer to Tables 1.5-1.7 to check whether, and if yes, when, an appropriate empirical therapy was prescribed, according to the participant’s presenting syndrome. This could be suspected meningitis, encephalitis, meningoencephalitis, brain abscess, or another brain infection, according to the diagnosis outcome assessment algorithms.</td><td>• A Yes/No/Not applicable answer, and a Date if Yes is selected, are required, IF the participant achieved a probable or confirmed microbiological diagnosis.<br>• Refer to Table 1.8 to check whether, and if yes, when, an appropriate definitive therapy was prescribed, according to the probable or confirmed pathogen.</td></tr>
<tr><td>Key principles</td><td colspan="2">• In each case, this should be the minimum reasonable therapy for a given presenting illness or pathogen, which should in theory be available at every hospital. The assessment does not consider availability or cost of the therapy.<br>• Dose of drugs is not captured, as often there is a range of appropriate doses and frequencies for anti-infective drugs, and they may be amended reasonably taking host, epidemiological and operational factors into account.<br>• Duration is also not considered, due to the focus on initial management, and because many patients are transferred to other facilities for ongoing care, so accurate course lengths are difficult to determine.</td></tr>
<tr><td>Special circumstances – each assessment</td><td>• The first anti-infective therapy prescribed may not be counted as appropriate empirical therapy. In this case, subsequent drugs should be assessed for appropriateness.<br>• Patients transferred with the appropriate therapy from another hospital would be counted as a Yes for appropriate empirical therapy on the date of presentation to the hospital, even if it is stopped at our study hospital for re-evaluation.<br>• Patients having same-day LP +/- imaging might feasibly have certain therapies withheld pending those results, and when these don’t reveal features of brain infections and/or alternative causes, they may never receive them. The answer for these patients will be ‘Not applicable’. For example:<br>    o Children between 6 months and 6 years old with fever and one (non-simple) seizure with no other neurological signs and who are systemically stable might have ceftriaxone withheld pending LP +/- CT brain.<br>    o Adults with encephalopathy and even focal neurological deficit might have aciclovir withheld pending early brain imaging and LP.</td><td>• The drug counted as appropriate definitive therapy could be for any of the probable or confirmed pathogens identified by our study assessment. The assessment stops at the first drug for any treatable pathogen identified in the assessment.<br>• Some pathogens have no specific therapy. For participants with only such pathogens as a probable or confirmed cause of their illness, the assessment should be answered as ‘Not applicable’.</td></tr>
<tr><td>Special circumstances – combined assessment</td><td colspan="2">• In some cases, especially bacterial pathogens causing brain infection, the appropriate empirical and definitive therapy may be the same drug.<br>• Sometimes a very narrow-spectrum anti-infective agent is used initially, which acts as appropriate definitive therapy, but does not provide appropriate empirical cover that is wide enough to treat common pathogens causing the presentation. In such cases, the assessor should decide if the information available to the clinician at the point of prescription (e.g. investigation results) meant that the narrow-spectrum treatment was also appropriate empirical cover for the remaining likely pathogens. However, this could also be judged as having achieved definitive but not empirical therapy, depending on the situation.</td></tr>
</table>

| | Empirical anti-infective therapy | Definitive anti-infective therapy |
|---|---|---|
| | • If a patient's circumstances mean that a judgement about a therapy is reached that differs from this protocol, a note should be made justifying why the assessment deviates from the protocol. | |

**Definition for suspected encephalitis**[23]

*This is for use in patients with an* ***absence of meningeal irritation****, who would otherwise attract a label of suspected meningoencephalitis.*

**1. Acute or sub-acute (less than four weeks) alteration in consciousness, cognition, personality or behaviour persisting for more than 24 hours**. Personality or behaviour change can include agitation, psychosis, somnolence, insomnia, catatonia, mood liability, altered sleep pattern and (in children) new-onset enuresis or irritability.

**AND**

**2. Any 2 of**:

- fever (≥38°C) or prodromal illness—acute or sub-acute
- new-onset seizures
- focal neurological signs of acute or sub-acute onset, including focal weakness, oromotor dysfunction, movement disorders (chorea, athetosis, dystonia, hemiballismus, stereotypies, orolingual dyskinesia and tics) Parkinsonism (bradykinesia, tremor, rigidity and postural instability) and amnesia
- pleocytosis (cerebrospinal fluid [CSF] white cell count of more than 4 cells per microliter)
- neuroimaging compatible with encephalitis (which includes normal imaging)
- electroencephalogram (EEG) compatible with encephalitis

## Table S1.5: Brazil empirical therapy

| *Age* | **Suspected Meningitis/Meningo-encephalitis** | **>65 years old or immune-compromised** | **Suspected Encephalitis** ***(See definition above)*** | **Suspected Abscess/Single brain lesion** | **Other** |
|---|---|---|---|---|---|
| ***<16 years*** | Cefotaxime/Ceftriaxone<br>OR<br>Meropenem | Not applicable | As Meningitis,<br>PLUS<br><br>Aciclovir | Cefotaxime/Ceftriaxone &<br>Metronidazole<br>OR<br>Meropenem | Judged on a case-by-case basis |
| ***≥16 years*** | Cefotaxime/Ceftriaxone<br>OR<br>Meropenem | As Meningitis,<br>PLUS<br><br>Ampicillin<br>OR<br>Meropenem (if Ceftriaxone given) | As Meningitis,<br>PLUS<br><br>Aciclovir | Cefotaxime/Ceftriaxone &<br>Metronidazole<br>OR<br>Meropenem | Judged on a case-by-case basis |

## Table S1.6: India empirical therapy

| *Age* | **Suspected Meningitis/ Meningo-encephalitis <5 days' duration** | **Suspected Meningitis/ Meningo-encephalitis ≥ 5 days' duration** | **>65 years old or immune-compromised** | **Rash, eschar or sepsis** | **Suspected Encephalitis *(See definition above)*** | **Suspected Abscess/Single brain lesion** | **Other** |
|---|---|---|---|---|---|---|---|
| ***<16 years*** | Cefotaxime/Ceftriaxone<br>OR<br>Meropenem | | Not applicable | As Meningitis,<br>PLUS<br><br>Doxycycline<br>OR Azithromycin | As Meningitis,<br>PLUS<br><br>Aciclovir | Cefotaxime/Ceftriaxone &<br>Metronidazole<br>OR<br>Meropenem | Judged on a case-by-case basis |
| ***≥16 years*** | Cefotaxime/Ceftriaxone<br>OR<br>Meropenem | Judged on a case-by-case basis | As Meningitis,<br>PLUS<br><br>Ampicillin<br>OR<br>Meropenem (if Ceftriaxone given) | As Meningitis,<br>PLUS<br><br>Doxycycline<br>OR Azithromycin | As Meningitis,<br>PLUS<br><br>Aciclovir | Cefotaxime/Ceftriaxone &<br>Metronidazole<br>OR<br>Meropenem | Judged on a case-by-case basis |

## Table S1.7: Malawi empirical therapy

| *Age* | **Suspected Meningitis/Meningo-encephalitis** | **Suspected Encephalitis** ***(See definition above)*** | **Suspected Abscess/Single brain lesion** | **Other** |
|---|---|---|---|---|
| ***<16 years*** | Cefotaxime/Ceftriaxone<br>OR<br>Meropenem | As Meningitis,<br>PLUS<br><br>Artesunate | Cefotaxime/Ceftriaxone<br>& Metronidazole<br>OR<br>Meropenem | Judged on a case-by-case basis |
| ***≥16 years*** | Cefotaxime/Ceftriaxone<br>OR<br>Meropenem | As Meningitis,<br>PLUS<br><br>Artesunate | Cefotaxime/Ceftriaxone<br>& Metronidazole<br>OR<br>Meropenem | Judged on a case-by-case basis |

## Table S1.8: Definitive anti-infective therapy regimens

| Pathogen | Appropriate Definitive Therapy | Notes |
|---|---|---|
| **BACTERIA** | | |
| *Escherichia coli* | Ceftriaxone/Cefotaxime<br>OR<br>Meropenem<br>*See Notes* | Other antibacterials may be appropriate in case of resistance to those listed here. However, not all antibacterials penetrate the blood-brain barrier adequately to reach high enough concentrations in the meninges or brain. A case-by-case review of this therefore may be needed when antibacterials not listed here are used for treatment. |
| *Haemophilus influenzae* | Ceftriaxone/Cefotaxime<br>OR<br>Meropenem | |
| *Klebsiella pneumoniae* | Ceftriaxone/Cefotaxime<br>OR<br>Ceftazidime<br>OR<br>Meropenem<br>*See Notes* | Other antibacterials may be appropriate in case of resistance to those listed here. However, not all antibacterials penetrate the blood-brain barrier adequately to reach high enough concentrations in the meninges or brain. A case-by-case review of this therefore may be needed when antibacterials not listed here are used for treatment. |
| *Leptospira* | Benzylpenicillin<br>OR<br>Ceftriaxone/Cefotaxime<br>OR<br>Doxycycline<br>OR<br>Meropenem | |
| *Mycobacterium tuberculosis* | Locally accepted regimen for anti-tuberculosis therapy, depending on risk for drug resistant TB. Accept 'ATT' or 'TB therapy' without mention of individual drugs. | A detailed evaluation of whether a regimen will adequately treat a resistant M.tuberculosis isolate is not required. |
| *Neisseria meningitidis* | Benzylpenicillin<br>OR<br>Ceftriaxone/Cefotaxime<br>OR<br>Chloramphenicol<br>OR<br>Meropenem | |
| *Orientia tsutsugamushi* | Doxycycline<br>OR<br>Azithromycin | |
| Other bacteria known to be community acquired nonsurgical brain infection pathogens (i.e. excluding coagulase-negative Staphylococci and similar organisms) | Judged on a case-by-case basis | |
| *Salmonellae* | Ceftriaxone/Cefotaxime<br>OR<br>Azithromycin<br>OR | Other antibacterials may be appropriate in case of resistance to those listed here. However, not all antibacterials penetrate the blood-brain barrier adequately to reach high enough concentrations in the meninges or brain. A case-by-case review of this therefore may be needed when antibacterials not listed here are used for treatment. |

| **Pathogen** | **Appropriate Definitive Therapy** | **Notes** |
|---|---|---|
| | Meropenem<br>*See Notes* | |
| *Streptococcus Group B* | Benzylpenicillin *(see Notes)*<br>OR<br>Ceftriaxone/Cefotaxime *(see Notes)*<br>OR<br>Meropenem *(see Notes)*<br>OR<br>Vancomycin | Benzylpenicillin, Ceftriaxone/Cefotaxime, or Meropenem might not be appropriate as monotherapy. Vancomycin or Rifampicin or other drugs may be needed in combination. Consider this on a case-by-case basis depending on background risk of penicillin/cephalosporin resistance and specific isolate susceptibilities. |
| *Streptococcus pneumoniae* | Benzylpenicillin *(see Notes)*<br>OR<br>Ceftriaxone/Cefotaxime *(see Notes)*<br>OR<br>Meropenem *(see Notes)*<br>OR<br>Vancomycin | Benzylpenicillin, Ceftriaxone/Cefotaxime, or Meropenem might not be appropriate as monotherapy. Vancomycin or Rifampicin or other drugs may be needed in combination. Consider this on a case-by-case basis depending on background risk of penicillin/cephalosporin resistance and specific isolate susceptibilities. |
| *Treponema pallidum* | Penicillin G<br>OR<br>Procaine penicillin<br>OR<br>Ceftriaxone | May be adequately treated with alternatives in case of severe beta-lactam allergy, such as Meropenem – case-by-case review of this therefore may be needed when antibacterials not listed here are used for treatment. |
| **FUNGI** | | |
| *Cryptococcus* | Amphotericin B (conventional or liposomal) & Flucytosine<br>OR<br>Amphotericin B (conventional or liposomal) & Fluconazole<br>*See Notes* | Voriconazole may be an alternative in specific cases – consider appropriateness of this if mentioned.<br>Fluconazole and/or flucytosine without amphotericin B would not be considered appropriate definitive therapy. |
| **PROTOZOA** | | |
| *Plasmodium falciparum* | Artesunate<br>OR<br>Quinine | |
| *Toxoplasma gondii* | Sulfadiazine-Pyrimethamine<br>OR<br>Co-trimoxazole/Sulfamethoxazole-Trimethoprim<br>OR<br>Clindamycin-Pyrimethamine<br>OR<br>Atovaquone-Pyrimethamine | |
| **VIRUSES** | | |
| *Chikungunya virus* | No known definitive therapy | |
| *Cytomegalovirus (CMV)* | Ganciclovir<br>OR<br>Foscarnet<br>OR | Valganciclovir (oral) may be considered appropriate in some cases. |

| **Pathogen** | **Appropriate Definitive Therapy** | **Notes** |
|---|---|---|
| | Cidofovir<br>*See Notes* | |
| *Dengue virus* | No known definitive therapy | |
| *Enteroviruses* | No known definitive therapy | |
| *Herpes simplex virus (HSV)* | Aciclovir | |
| *Japanese encephalitis virus (JEV)* | No known definitive therapy | |
| *Nipah virus* | No known definitive therapy (see Notes) | Ribavirin is experimental, so should not be expected to be used in all cases. |
| *Parechovirus* | No known definitive therapy | |
| *Rabies virus* | No known definitive therapy | |
| *Rubella* | No known definitive therapy | |
| *Varicella zoster virus (VZV)* | Aciclovir | |
| *West Nile virus (WNV)* | No known definitive therapy | |
| *Zika virus* | No known definitive therapy | |

## 7. References


1 World Health Organization. Monitoring the building blocks of health systems: a handbook of indicators and their measurement strategies. Geneva: World Health Organization, 2010 https://iris.who.int/handle/10665/258734.

2 Skivington K, Matthews L, Simpson SA, *et al.* A new framework for developing and evaluating complex interventions: update of Medical Research Council guidance. *BMJ* 2021; **374**: n2061.

3 Wight D, Wimbush E, Jepson R, Doi L. Six steps in quality intervention development (6SQuID). *J Epidemiol Community Health* 2016; **70**: 520–5.

4 Michie S, van Stralen MM, West R. The behaviour change wheel: a new method for characterising and designing behaviour change interventions. *Implement Sci IS* 2011; **6**: 42.

5 Granerod J, Cunningham R, Zuckerman M, *et al.* Causality in acute encephalitis: defining aetiologies. *Epidemiol Infect* 2010; **138**: 783–800.

6 Singh B, Lant S, McGill F. Defining causality in acute brain infections. OSF Preprints 2025. DOI:10.31219/osf.io/b3j8z_v1. Available at: https://osf.io/preprints/osf/b3j8z_v1.

7 Bernal JL, Cummins S, Gasparrini A. Interrupted time series regression for the evaluation of public health interventions: a tutorial. *Int J Epidemiol* 2017; **46**: 348–55.

8 Lau B, Cole SR, Gange SJ. Competing risk regression models for epidemiologic data. *Am J Epidemiol* 2009; **170**: 244–56.

9 Devlin N, Parkin D, Janssen B. Methods for Analysing and Reporting EQ-5D Data. Cham (CH), 2020 DOI:10.1007/978-3-030-47622-9.

10 Arlotti M, Grossi P, Pea F, *et al.* Consensus document on controversial issues for the treatment of infections of the central nervous system: bacterial brain abscesses. *Int J Infect Dis IJID Off Publ Int Soc Infect Dis* 2010; **14 Suppl 4**: S79-92.

11 Brazil Ministry of Health, Ministry of Health, Health Surveillance Secretariat. Health Surveillance Guide: Volume 1, 3rd edn. Brasília: Ministry of Health, 2019 https://bvsms.saude.gov.br/bvs/publicacoes/guia_vigilancia_saude_3ed.pdf.

12 Le Saux N. Position Statement: Guidelines for the management of suspected and confirmed bacterial meningitis in Canadian children older than 2 months of age. Ottawa: Canadian Paediatric Society, 2020 https://cps.ca/en/documents/position/management-of-bacterial-meningitis.

13 Department of Medicine, College of Medicine, Malawi. The Clinical Book, 2nd edn. Blantyre: College of Medicine, 2012.

14 Paediatric & Child Health Dept, Queen Elizabeth Central Hospital & College of Medicine, Malawi. Protocols for the management of common childhood illnesses in Malawi, 2nd edn. Blantyre: College of Medicine, 2011.

15 Domingues RB, Teixeira AL. Management of acute viral encephalitis in Brazil. *Braz J Infect Dis Off Publ Braz Soc Infect Dis* 2009; **13**: 433–9.

16 Indian Council of Medical Research. Treatment Guidelines for Antimicrobial Use in Common Syndromes. New Delhi: Indian Council of Medical Research, 2019 https://main.icmr.nic.in/sites/default/files/guidelines/Treatment_Guidelines_2019_Final.pdf.

17 McGill F, Heyderman RS, Michael BD, *et al.* The UK joint specialist societies guideline on the diagnosis and management of acute meningitis and meningococcal sepsis in immunocompetent adults. *J Infect* 2016; **72**: 405–38.

18 Antimicrobial Therapy, Inc. The Sanford Guide to Antimicrobial Therapy, 53rd edn. Sperryville, VA, USA: Antimicrobial Therapy, Inc., 2021.

19 Sonneville R, Ruimy R, Benzonana N, *et al.* An update on bacterial brain abscess in immunocompetent patients. *Clin Microbiol Infect Off Publ Eur Soc Clin Microbiol Infect Dis* 2017; **23**: 614–20.

20 van de Beek D, Brouwer MC, Thwaites GE, Tunkel AR. Advances in treatment of bacterial meningitis. *Lancet Lond Engl* 2012; **380**: 1693–702.

21 van de Beek D, Cabellos C, Dzupova O, *et al.* ESCMID guideline: diagnosis and treatment of acute bacterial meningitis. *Clin Microbiol Infect Off Publ Eur Soc Clin Microbiol Infect Dis* 2016; **22 Suppl 3**: S37-62.

22 Focaccia R, Siciliano RF (eds). Tratado de infectologia, 6th edn. Rio de Janeiro: Atheneu, 2021.

23 Backman R, Foy R, Diggle PJ, *et al.* A pragmatic cluster randomised controlled trial of a tailored intervention to improve the initial management of suspected encephalitis. *PloS One* 2018; **13**: e0202257.

## 8. Brain Infections Global Intervention Study Group

| First Name | Second/ Family Name | Institution 1 | Institution 2 | Study concept-ualisation & methodology | Data curation & validation | Data analysis & interp-retation | Funding acquisition | Super-vision | Project administration & study implementation | Writing - Manuscript review |
|---|---|---|---|---|---|---|---|---|---|---|
| Ajith | Sivadasan | Christian Medical College, Vellore, India | | X | | | X | X | X | X |
| Alex | Reginald | Christian Medical College, Vellore, India - Chittoor campus | | | | | | | X | X |
| Alex | Shabani | Malawi Liverpool Wellcome Trust, Blantyre, Malawi | | | X | | | | X | X |
| Aline | de Moura Brasil Matos | Instituto de Medicina Tropical, Universidade de São Paulo, São Paulo, Brazil | | | | X | | | | X |
| Anandhi | Arumugam | Christian Medical College, Vellore, India | | | X | X | | | X | X |
| Anbu | Suresh Rao | Scudder Memorial Hospital, Vellore, India | | | | | | | X | X |
| André | Silva Lira de Lucena | Oswaldo Cruz Foundation (Fiocruz), Recife, Pernambuco, Brazil | | | X | X | | | X | X |
| Angel | Miraclin | Christian Medical College, Vellore, India | | | | X | | | X | X |
| Anitha | Aswathanarayan | RL Jalappa Hospital, Bangalore, India | | | X | | | | X | X |
| Anna | Rosala-Hallas | Liverpool Clinical Trials Centre, University of Liverpool, Liverpool, UK | | X | | | | | | |
| Anna | Simon | Christian Medical College, Vellore, India | | | | | | | X | X |
| Anushri | Somasundaran | Institute of Infection, Veterinary and Ecological Sciences, University of Liverpool, Liverpool, United Kingdom | | | X | X | | | X | X |
| Aparna | Vasudev | National Institute of Mental Health and Neurosciences, Bangalore, India | | | X | X | | | X | X |
| Archana | | National Institute of Mental Health and Neurosciences, Bangalore, India | | | X | X | | | X | X |
| Arnold E | Kapachika | Ministry of Health, Malawi | | | | | | X | X | X |

| First Name | Second/ Family Name | Institution 1 | Institution 2 | Study concept-ualisation & methodology | Data curation & validation | Data analysis & interp-retation | Funding acquisition | Super-vision | Project administration & study implementation | Writing - Manuscript review |
|---|---|---|---|---|---|---|---|---|---|---|
| Arvind | Natarajan | RL Jalappa Hospital, Bangalore, India | | | | | | | X | X |
| Audrin | Lenin | Christian Medical College, Vellore, India | | | X | X | | | X | X |
| Balaji | Veeraraghavan | Christian Medical College, Vellore, India | | | | | | | X | X |
| Blessings | Kadzuwa | Chiradzulu District Hospital, Malawi | | | X | | | | X | X |
| Brigitte | Denis | Malawi Liverpool Wellcome Trust, Blantyre, Malawi | | | | | | | X | X |
| Catherine | Anscombe | Malawi Liverpool Wellcome Trust, Blantyre, Malawi | | | | | | | X | X |
| Chimwemwe | Maluwa | Machinga District Hospital, Malawi | | | X | | | | X | X |
| Chishala | Chafunya | Malawi Liverpool Wellcome Trust, Blantyre, Malawi | | | X | | | | X | X |
| Chitra | Pattabiraman | National Institute of Mental Health and Neurosciences, Bangalore, India | | | | | | | X | X |
| Clifford | Chitala | Chiradzulu District Hospital, Malawi | | | X | | | | X | X |
| Daisy | Sampreetha | National Institute of Mental Health and Neurosciences, Bangalore, India | | | X | X | | | X | X |
| Debasis Das | Adhikari | Christian Medical College, Vellore, India | | | | | | | X | X |
| Dhanalakshmi | | SNR Hospital, Kolar, India | | | | | | | X | X |
| Divya | Deodhar | Christian Medical College, Vellore, India | | | X | X | | | X | X |
| Divya | Mathew | Christian Medical College, Vellore, India | | | X | | | | X | X |
| Durjoy | Lahiri | National Institute for Health Research Health Protection Research Unit in Emerging and Zoonotic Infections, Institute of Infection, Veterinary and Ecological Sciences, University of Liverpool, Liverpool, United Kingdom | Bangur Institute of Neurosciences, Institute of Post-Graduate Medical Education and Research, Kolkata, India | | | X | | | | X |

| First Name | Second/ Family Name | Institution 1 | Institution 2 | Study concept-ualisation & methodology | Data curation & validation | Data analysis & interp-retation | Funding acquisition | Super-vision | Project administration & study implementation | Writing - Manuscript review |
|---|---|---|---|---|---|---|---|---|---|---|
| Elizabeth | Rodgers | Meningitis Research Foundation-UK | | X | | X | | | | X |
| Eva Maria | Hodel | Institute of Infection, Veterinary and Ecological Sciences, University of Liverpool, Liverpool, United Kingdom | Institute of Social and Preventive Medicine, University of Bern, Bern, Switzerland | | X | | | | X | X |
| Evelyn | López | National Institute for Health Research Health Protection Research Unit in Emerging and Zoonotic Infections, Institute of Infection, Veterinary and Ecological Sciences, University of Liverpool, Liverpool, United Kingdom | | | X | X | | | X | X |
| Eveness | Chiipanthenga | Malawi Liverpool Wellcome Trust, Blantyre, Malawi | | | X | | | | X | X |
| Felix | Jamu | Malawi Liverpool Wellcome Trust, Blantyre, Malawi | | | X | | | | X | X |
| Gina | Chandy | Christian Medical College, Vellore, India | | | X | X | | | X | X |
| Gnanadurai John | Fletcher | Christian Medical College, Vellore, India | | | | | | | | |
| Gopalkrishna | Gururaj | National Institute of Mental Health and Neurosciences, Bangalore, India | | | | | | X | X | X |
| Greta | Wood | Institute of Infection, Veterinary and Ecological Sciences, University of Liverpool, Liverpool, United Kingdom | | X | X | | | | | X |
| Gurrapu | Rakesh | National Institute of Mental Health and Neurosciences, Bangalore, India | | | X | X | | | X | X |
| GV | Basavaraja | Indira Gandhi Institute of Child Health, Bangalore, India | | | | | | X | X | X |
| Hannah | Persis | National Institute of Mental Health and Neurosciences, Bangalore, India | | | X | X | | | X | X |
| J | Vignesh Kumar | Christian Medical College, Vellore, India | | X | X | X | | | X | X |

| First Name | Second/ Family Name | Institution 1 | Institution 2 | Study concept-ualisation & methodology | Data curation & validation | Data analysis & interp-retation | Funding acquisition | Super-vision | Project administration & study implementation | Writing - Manuscript review |
|---|---|---|---|---|---|---|---|---|---|---|
| Jagadeesh | Munichannappa | District Health Office, Kolar, India | | | | | | | X | X |
| Jagan | S | National Institute of Mental Health and Neurosciences, Bangalore, India | | | X | X | | | X | X |
| Jailson B | Correia | Instituto de Medicina Integral Professor Fernando Figueira (IMIP), Recife, Brazil | | | | X | | | X | X |
| James | Tovey | Liverpool Clinical Trials Centre, University of Liverpool, Liverpool, United Kingdom | | | X | | | | | X |
| Janet | Harrison | Liverpool Clinical Trials Centre, University of Liverpool, Liverpool, United Kingdom | | | X | | | | | X |
| Jenala | Njirammadzi | Queen Elizabeth Central Hospital, Blantyre, Malawi | | | | | | X | X | X |
| Jenevi Margaret | Mendosa | National Institute of Mental Health and Neurosciences, Bangalore, India | | | X | X | | | X | X |
| John Jude Anthony | Prakash | Christian Medical College, Vellore, India | | | | | | | X | X |
| Jones | Kadewere | Thyolo District Hospital, Thyolo, Malawi | | | X | | | | X | X |
| Jyoti | Sharma | Christian Medical College, Vellore, India | | | X | X | | | X | X |
| Karen | Lobo | National Institute of Mental Health and Neurosciences, Bangalore, India | | | X | X | | | X | X |
| Karthik | Gunasekaran | Christian Medical College, Vellore, India | | | | X | | | X | X |
| Kasi | Sekar | Centre for Psycho Social Support in Disaster Management, National Institute of Mental Health and Neurosciences, Bangalore, India | | X | | X | | X | X | X |
| Kaustubh | Somalwar | National Institute of Mental Health and Neurosciences, Bangalore, India | | | X | X | | | X | X |

| First Name | Second/ Family Name | Institution 1 | Institution 2 | Study concept-ualisation & methodology | Data curation & validation | Data analysis & interp-retation | Funding acquisition | Super-vision | Project administration & study implementation | Writing - Manuscript review |
|---|---|---|---|---|---|---|---|---|---|---|
| Keshav | Murthy | Indira Gandhi Institute of Child Health, Bangalore, India | | | | | | | X | X |
| Lakshmi | | SNR Hospital, Kolar, India | | | | | | | X | X |
| Liam | Whittle | Liverpool Clinical Trials Centre, University of Liverpool, Liverpool, United Kingdom | | X | | X | | | | X |
| Lucia | Jansi Rani S | Christian Medical College, Vellore, India | | | X | X | | | X | X |
| Madalitso | Kalima | Chiradzulu District Hospital, Malawi | | | X | | | | X | X |
| Madhu | Sudan | National Institute of Mental Health and Neurosciences, Bangalore, India | | | X | X | | | X | X |
| Mahanthesh | | Indira Gandhi Institute of Child Health, Bangalore, India | | | | X | | | X | X |
| Mandara | Ganganakudige Manjappaiah | National Institute of Mental Health and Neurosciences, Bangalore, India | | | | X | | | | X |
| Maria Ellen | da Silva Antonio | Oswaldo Cruz Foundation (Fiocruz), Recife, Pernambuco, Brazil | | | X | X | | | X | X |
| Matthew | Smyth | National Institute for Health Research Health Protection Research Unit in Emerging and Zoonotic Infections, Institute of Infection, Veterinary and Ecological Sciences, University of Liverpool, Liverpool, United Kingdom | | | X | X | | | X | X |
| Mavis | Menyere | Malawi Liverpool Wellcome Trust | | | | | | | X | X |
| Memory | Siwombo | Queen Elizabeth Central Hospital, Blantyre, Malawi | | | | X | | | X | X |
| Monica | Kamwana | Malawi Liverpool Wellcome Trust, Blantyre, Malawi | | | X | | | | X | X |
| Morganna | Costa Lima | Oswaldo Cruz Foundation (Fiocruz), Recife, Pernambuco, Brazil | | | X | | | | X | X |

| First Name | Second/ Family Name | Institution 1 | Institution 2 | Study concept-ualisation & methodology | Data curation & validation | Data analysis & interp-retation | Funding acquisition | Super-vision | Project administration & study implementation | Writing - Manuscript review |
|---|---|---|---|---|---|---|---|---|---|---|
| Muniraju | SR | National Institute of Mental Health and Neurosciences, Bangalore, India | | | X | X | | | X | X |
| Nalini | Newbigging | Christian Medical College, Vellore, India | | | X | | | | X | X |
| Nathalie | van den Brekel | National Institute for Health Research Health Protection Research Unit in Emerging and Zoonotic Infections, Institute of Infection, Veterinary and Ecological Sciences, University of Liverpool, Liverpool, United Kingdom | | | | X | | | | X |
| Navya | C | National Institute of Mental Health and Neurosciences, Bangalore, India | | | X | X | | | X | X |
| Nihal | Thomas | Christian Medical College, Vellore, India - Chittoor campus | | | | | | X | X | X |
| Philip | Sajiwa | Thyolo District Hospital, Thyolo, Malawi | | | X | | | | X | X |
| Prabhakar | K | RL Jalappa Hospital, Bangalore, India | | | | | | | X | X |
| Prasannakuma r | Palanikumar | Christian Medical College, Vellore, India | | | X | X | | | X | X |
| Priscilla | Salley | Malawi Liverpool Wellcome Trust, Blantyre, Malawi | | | X | | | | X | X |
| Priya | SK | Scudder Memorial Hospital, Vellore, India | | | | | | | X | X |
| Rachael | Brookes | Institute of Infection, Veterinary and Ecological Sciences, University of Liverpool, Liverpool, United Kingdom | | | | X | | | | X |
| Radhika | | SNR Hospital, Kolar, India | | | X | | | | X | X |
| Ritika | Thakur | National Institute of Mental Health and Neurosciences, Bangalore, India | | | X | X | | | X | X |

| First Name | Second/ Family Name | Institution 1 | Institution 2 | Study concept-ualisation & methodology | Data curation & validation | Data analysis & interp-retation | Funding acquisition | Super-vision | Project administration & study implementation | Writing - Manuscript review |
|---|---|---|---|---|---|---|---|---|---|---|
| Rituwij | Kumar | National Institute of Mental Health and Neurosciences, Bangalore, India | | | X | X | | | X | X |
| Samuel George | Hansdak | Christian Medical College, Vellore, India | | | | | | | | |
| Sanjith | Aaron | Christian Medical College, Vellore, India | | | | | | | X | X |
| Santhoshkumar | Rajendran | Christian Medical College, Vellore, India | | | X | | | | X | X |
| Santosh | Chaturvedi | National Institute of Mental Health and Neurosciences, Bangalore, India | | | | | X | | | X |
| Sathish | Kumar | Christian Medical College, Vellore, India | | | | | | | X | X |
| Sathya | Prabhu | Christian Medical College, Vellore, India | | | X | X | | | X | X |
| Shoba | Mammen | Christian Medical College, Vellore, India | | | | | | | X | X |
| Sithembile | Bilima | Malawi Liverpool Wellcome Trust | | | | | | | X | X |
| Sithembinkosi | Mhlanga | Institute of Infection, Veterinary and Ecological Sciences, University of Liverpool, Liverpool, United Kingdom | | | X | | | | X | X |
| Sneha Deena | Varkki | Christian Medical College, Vellore, India | | | | | | | X | X |
| Sofia R | Valdoleiros | Infectious Diseases Department, Centro Hospitalar Universitário de São João, Porto, Portugal | Faculty of Medicine, University of Porto, Porto, Portugal | X | | | | | | X |
| Srihari | | SNR Hospital | | | | | | | X | X |
| Srinath | Chadam | SNR Hospital | | | | | | | X | X |
| Stephen | Ray | Institute of Infection, Veterinary and Ecological Sciences, University of Liverpool, Liverpool, United Kingdom | | X | | | X | | X | X |

| First Name | Second/ Family Name | Institution 1 | Institution 2 | Study concept-ualisation & methodology | Data curation & validation | Data analysis & interp-retation | Funding acquisition | Super-vision | Project administration & study implementation | Writing - Manuscript review |
|---|---|---|---|---|---|---|---|---|---|---|
| Sudha | Reddy VR | National Institute of Mental Health and Neurosciences, Bangalore, India | | | X | | | | X | X |
| Thaíse Yasmine | Vasconcelos de Lima Cavalcanti | Oswaldo Cruz Foundation (Fiocruz), Recife, Pernambuco, Brazil | | | X | | | | X | X |
| Tina | Damodar | National Institute of Mental Health and Neurosciences, Bangalore, India | | | | | | | X | X |
| Trudie | Lang | Nuffield Department of Medicine, Oxford University, UK | | | | | X | | X | X |
| Uddhav | Kinhal | IGICH, Bangalore, India | | | X | | | | X | X |
| Vasundharaa | S Nair | National Institute of Mental Health and Neurosciences, Bangalore, India | Jindal Institute of Behavioural Sciences, OP Jindal Global University. | X | X | X | | | X | X |
| Vijaykumar | SN | District Health Office, Kolar, India | | | | | | | X | X |
| Vikram | Holla | National Institute of Mental Health and Neurosciences, Bangalore, India | | | X | X | | | X | X |

## 9. Ethics committee approvals

| Country | Committee Name | Approval number |
|---|---|---|
| Brazil | FIOCRUZ / CPQAM Aggeu Magalhães Research Center, Recife, Pernambuco | 58932122.8.0000.5190 |
| India | Health Ministry Screening Committee, Indian Council of Medical Research | 2019-7983 |
| Malawi | National Health Sciences Research Committee | 2203 |
| UK | University of Liverpool Central University Research Ethics Committee | 5350 |

One overarching committee is listed for each country
