# Supplementary material for: A multifaceted intervention to improve diagnosis and early management of hospitalised patients with suspected acute brain infections in Brazil, India, and Malawi: an international multicentre intervention study
Source: Lancet. Author manuscript; Available in PMC 2025 Oct 28. (PMC7618301; doi:10.1016/S0140-6736(25)00263-6)
Supplement: Supplementary Materials [file EMS209560-supplement-Supplementary_Materials.zip › 1-s2.0-S0140673625002636-mmc2.pdf]

THE LANCET

Supplementary appendix 2

This appendix formed part of the original submission and has been peer reviewed. We post it as supplied by the authors.

Supplement to: Singh B, Lipunga GD, Thangavelu P, et al. A multifaceted intervention to improve diagnosis and early management of hospitalised patients with suspected acute brain infections in Brazil, India, and Malawi: an international multicentre intervention study. *Lancet* 2025; published online March 10. https://doi.org/10.1016/S0140-6736(25)00263-6.

Supplementary Appendix 2: Additional Figures and Tables

Table of contents

FIGURE S2.1: OVERALL STUDY DESIGN 2
FIGURE S2.2: CORE COMPONENTS OF THE INTERVENTION 3
FIGURE S2.3: CLINICAL ALGORITHM DEVELOPED FOR THE INTERVENTION: EXAMPLE FROM VELLORE, INDIA 4
FIGURE S2.4: GUIDANCE FOR THE LUMBAR PUNCTURE PACK: EXAMPLES FROM HOSPITAL CORREIA PICANÇO, BRAZIL, AND RL JALAPPA HOSPITAL, BANGALORE CENTRE, INDIA 5
FIGURE S2.5: DIAGNOSTIC TESTING PANEL USED IN THE INTERVENTION: BRAZIL 6
FIGURE S2.6: DIAGNOSTIC TESTING PANEL USED IN THE INTERVENTION: INDIA 7
FIGURE S2.7: DIAGNOSTIC TESTING PANEL USED IN THE INTERVENTION: MALAWI 8
FIGURE S2.8: ACHIEVEMENT OF SYNDROMIC DIAGNOSIS BY MONTH, IN EACH CENTRE 9
FIGURE S2.9: PATHOGENS IDENTIFIED AS CONFIRMED OR PROBABLE CAUSES OF SUSPECTED ACUTE BRAIN INFECTION 10
FIGURE S2.10: ACHIEVEMENT OF MICROBIOLOGICAL DIAGNOSIS BY MONTH 11
FIGURE S2.11: TIME TO LUMBAR PUNCTURE, OVERALL AND IN EACH CENTRE 12
FIGURE S2.12: TIME TO APPROPRIATE EMPIRICAL THERAPY, OVERALL AND IN EACH CENTRE 13
FIGURE S2.13: LIVERPOOL OUTCOME SCORE AT DISCHARGE FROM HOSPITAL AND AT 30-DAY FOLLOW-UP, OVERALL 14
TABLE S2.1: CONFIRMED AND PROBABLE PATHOGENS DIAGNOSED IN EACH CENTRE AND OVERALL 15
TABLE S2.2: CONFIRMED AND PROBABLE PATHOGENS DIAGNOSED PRE- AND POST-INTERVENTION 16
TABLE S2.3: SECONDARY OUTCOME RESULTS, BY CENTRE 17
TABLE S2.4: PROCESS MEASURES FOR PATHOGEN TESTING 20
TABLE S2.5: COST OF THE INTERVENTION IN EACH CENTRE 21
TABLE S2.6: SYNDROMIC DIAGNOSES ACHIEVED 22
TABLE S2.7: SENSITIVITY, SUBGROUP AND POST HOC ANALYSES FOR THE PRIMARY OUTCOMES IN THE OVERALL DATASET 23

Figure S2.1: Overall study design

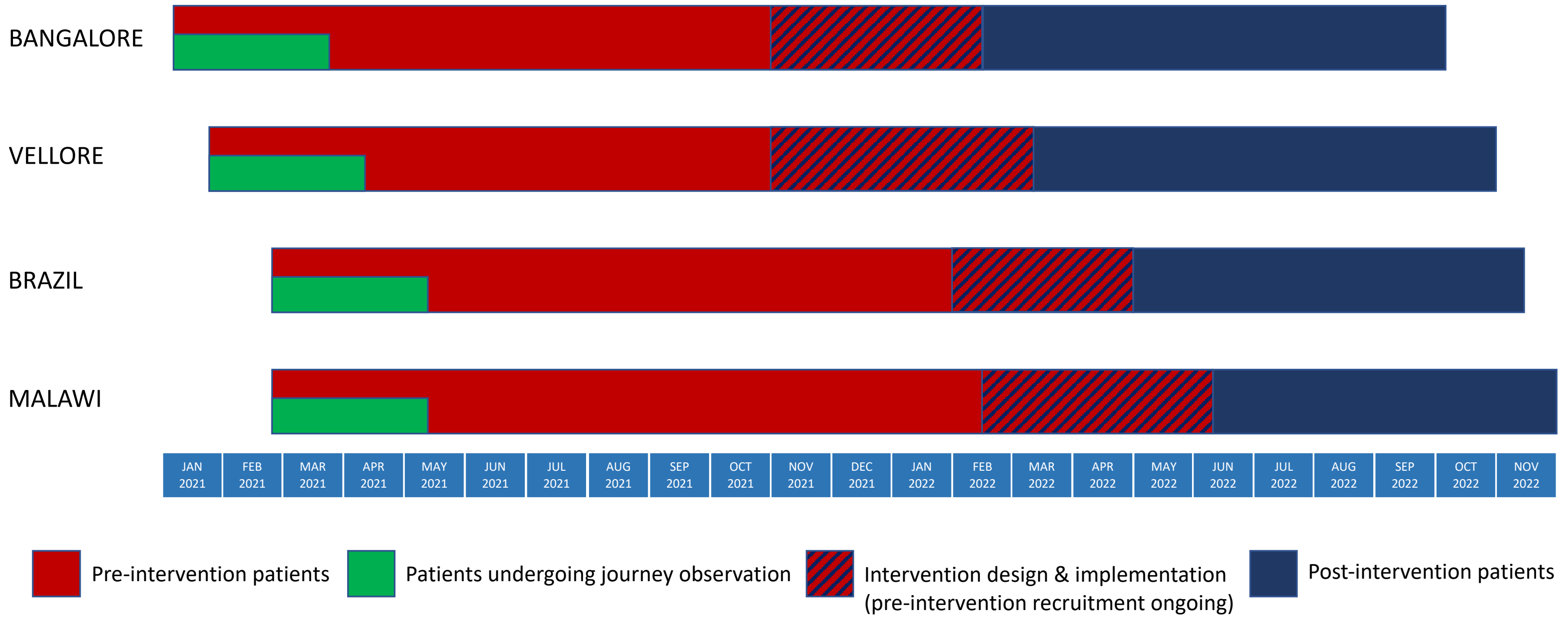

Recruitment and intervention implementation commenced at each centre when logistically possible, in line with the pragmatic study design. Detailed patient journeys were observed for patients recruited early in the pre-intervention phase.

Figure S2.2: Core components of the intervention

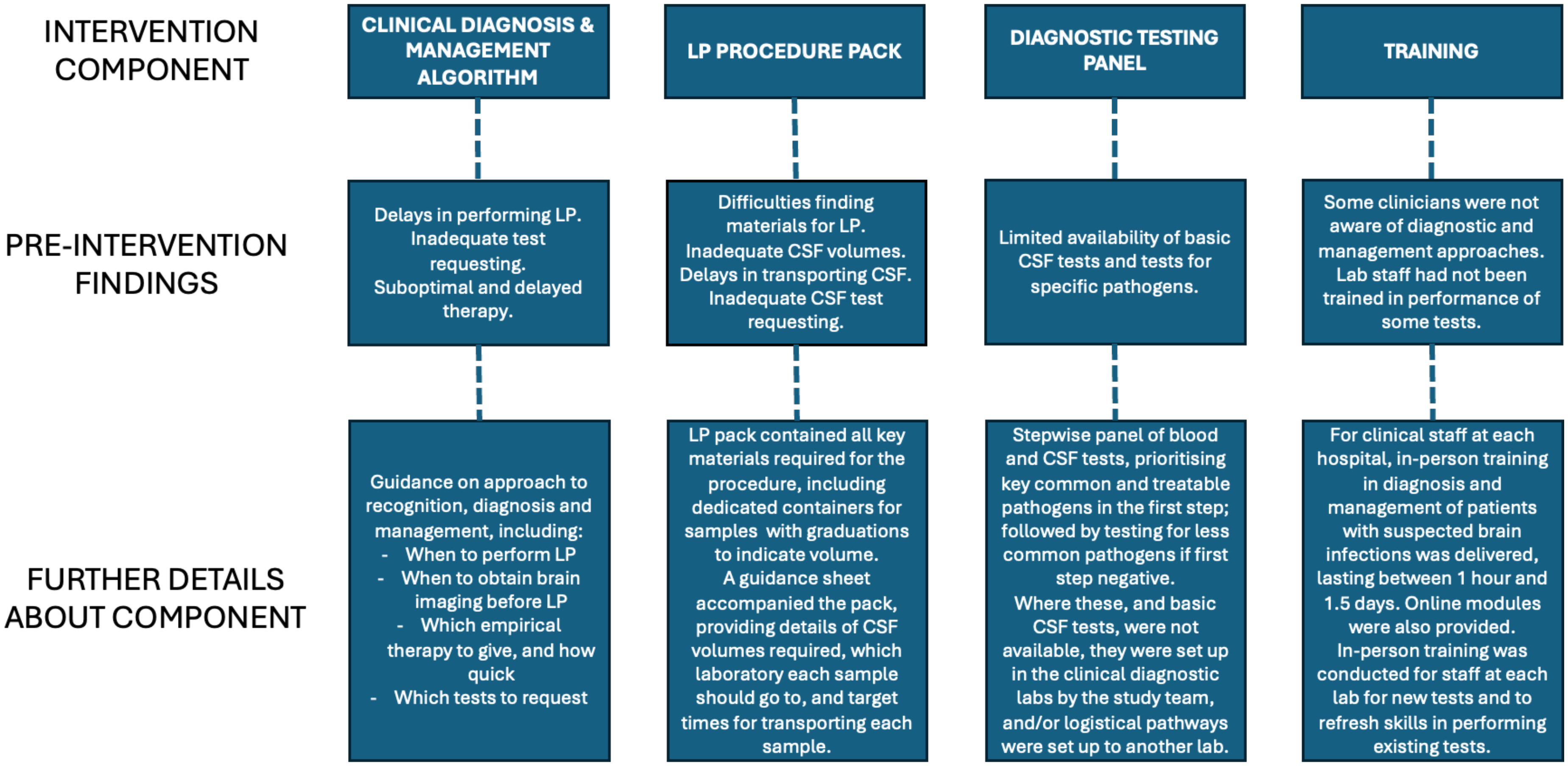

CSF, cerebrospinal fluid; LP, lumbar puncture

Figure S2.3: Clinical algorithm developed for the intervention: example from Vellore, India

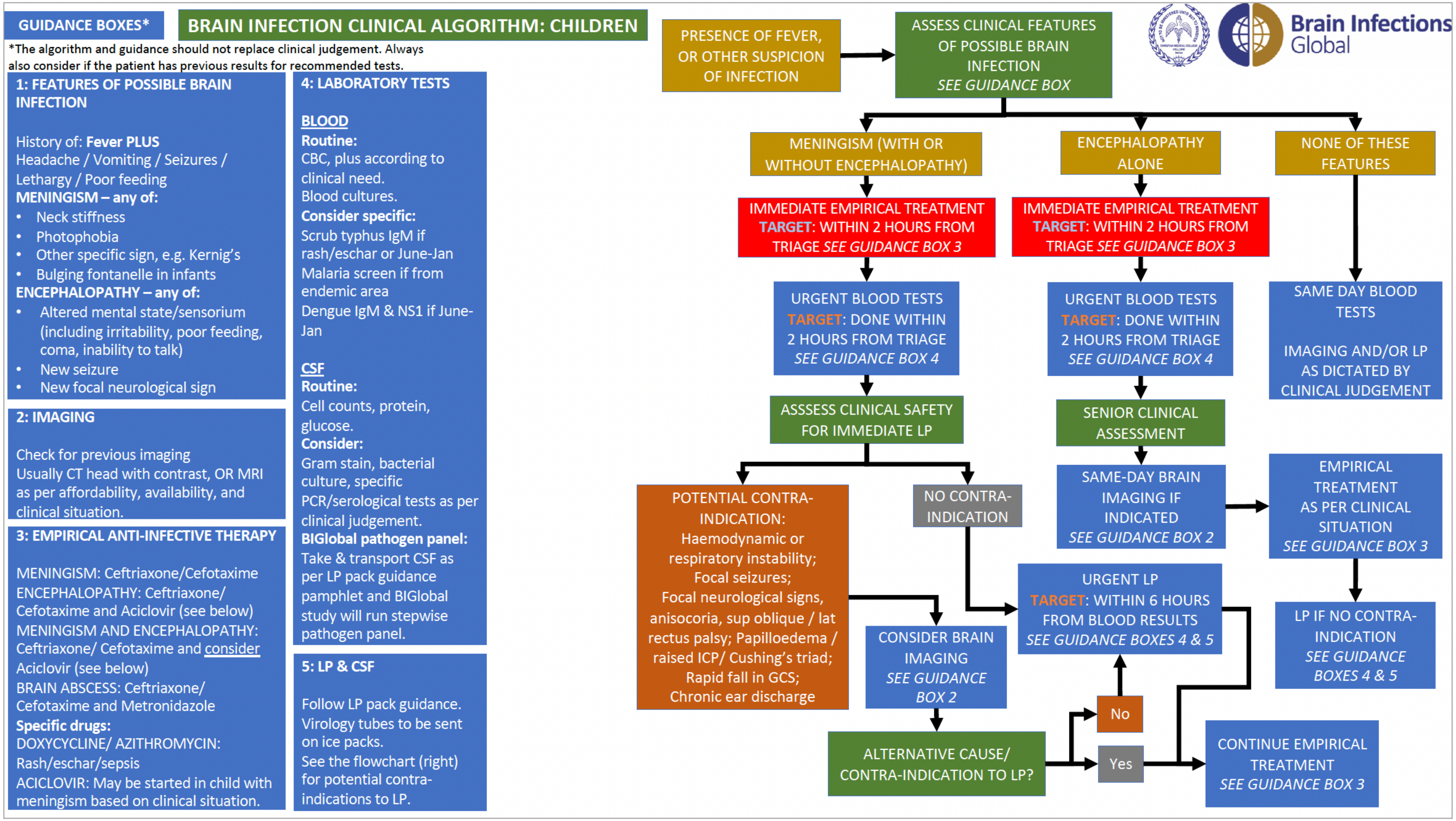

Figure S2.4: Guidance for the lumbar puncture pack: examples from Hospital Correia Picanço, Brazil, and RL Jalappa Hospital, Bangalore centre, India

Folheto de Orientação do Pacote de Punção Lombar - HOSPITAL CORREIA PICANÇO

RECIPIENTE NUMERO	RECIPIENTE	VOLUME MÍNIMO^	EXAMES		
1	Tubo de Cultura, com ágar chocolate	Adulto: 40 GOTAS (2 mL) Criança: 20 GOTAS (1 mL)	Cultura bacteriana	DENTRO DE 30 minutos de PL	HCP LABORATÓRIO
2	Tubo de Ensaio	Adulto: 40 GOTAS (2 mL) Criança: 20 GOTAS (1 mL)	Proteína e glicose (Realize a glicemia dentro de 1 hora de LP para interpretação)<br>Outros exames	DENTRO DE 30 minutos de PL	HCP LABORATÓRIO
3	Lâmina de vidro	1-2 GOTAS	Celularidade com diferencial<br>Gram	DENTRO DE 30 minutos de PL	HCP LABORATÓRIO
4	1.5 mL Tubo 'Eppendorf'	Adulto: 30 GOTAS (1.5 mL) Criança: 20 GOTAS (1 mL)	Anticorpo/antigeno e PCR exames – LACEN	DENTRO DE 30 minutos de PL	HCP LABORATÓRIO
5	1.5 mL Tubo 'Eppendorf'	Adulto: 30 GOTAS (1.5 mL) Criança: 20 GOTAS (1 mL) *MAIS 5 mL de sangue - em tubo de sangue vermelho, para exames BIGlobal*	Brain Infections Global estudo exames – Fiocruz*<br>(Anticorpo/antigeno e PCR exames conforme protocolo de estudo)	DENTRO DE 30 minutos de PL	HCP LABORATÓRIO

^ Volumes a serem modificados a critério do medico por exemplo:
- Neonatos e lactentes
- Crianças e adultos com agitação
- LCR muito viscoso ou purulento

* Na impossibilidade de coleta 2 Eppendorfs, priorisar pelo menos um para Fiocruz

Versão: 1 Agosto 2022

RL JALAPPA HOSPITAL LP Pack Guidance Pamphlet

Container No.	Container	Min CSF Volume^	Tests		
1	5mL graduated tube	1 mL (2 mL if TB suspected)	Gram, India ink & ZN (AFB) stains; bacterial & fungal culture<br>Other microbiological tests as required	WITHIN 30 mins of LP	MICROBIOLOGY LAB
2	5mL graduated tube	As per tests requested	Any other tests required	WITHIN 30 mins of LP	MICROBIOLOGY LAB
3	5mL graduated tube	Adults: 3 mL Children: 1 mL<br>*PLUS 5 mL blood - in red blood tube, for BIGlobal tests*	Brain Infections Global study samples<br>Antibody/antigen and PCR tests on CSF & *blood* as per study protocol	WITHIN 30 mins of LP	MICROBIOLOGY LAB
4	5mL graduated tube	1 mL (0.5mL for children)	Cell count and typing	WITHIN 30 mins of LP	CYTOLOGY LAB (HAEMATOLOGY LAB BETWEEN 4PM & 9AM)
5	5mL graduated tube	1 mL (0.5mL for children)	Protein & Glucose *(Perform random blood sugar within 1 hour of LP for interpretation)*	WITHIN 30 mins of LP	BIOCHEMISTRY LAB

^ Volumes to be modified as per clinician's discretion

Figure S2.5: Diagnostic testing panel used in the intervention: Brazil

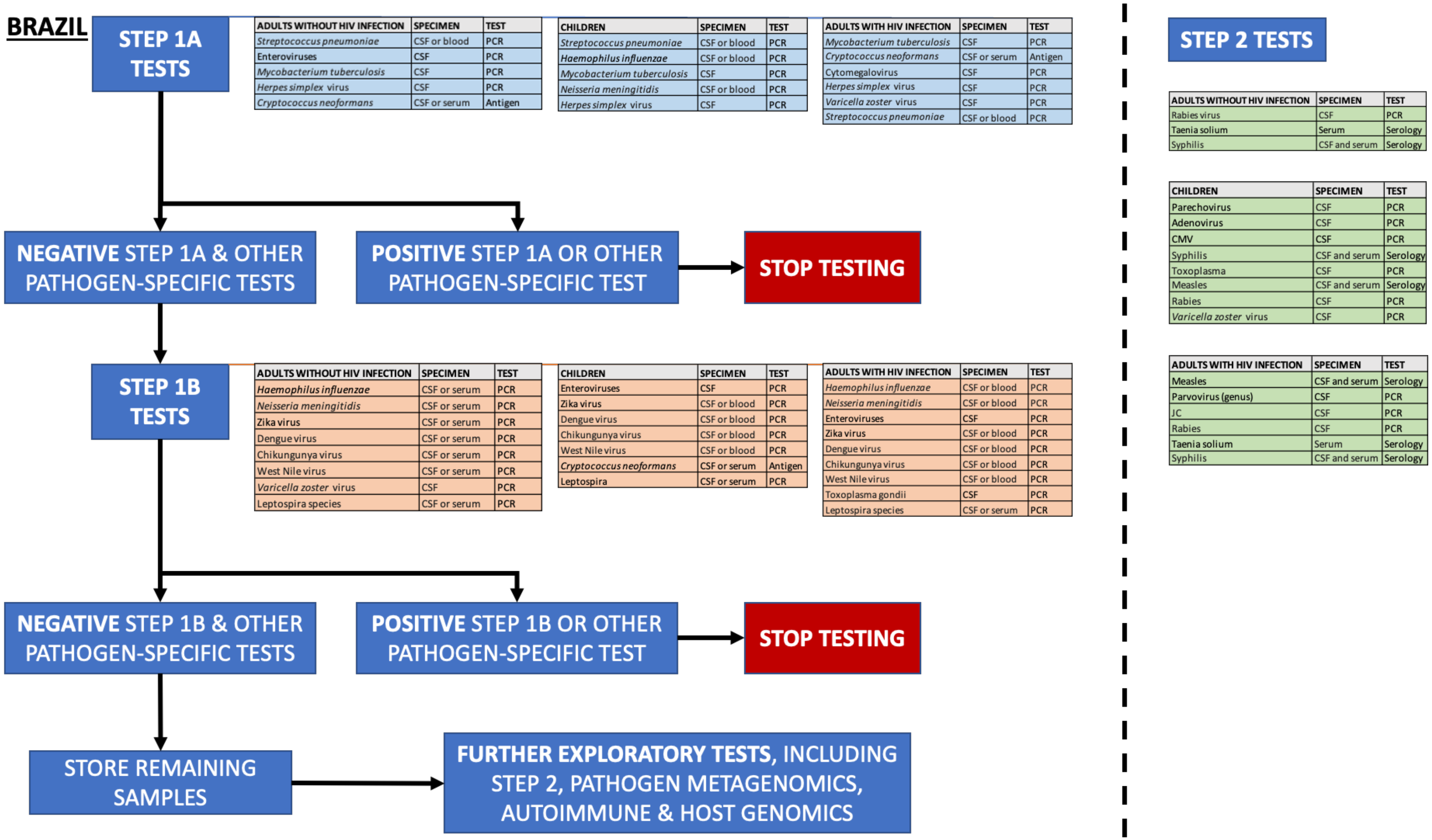

STEP 1A TESTS

ADULTS WITHOUT HIV INFECTION	SPECIMEN	TEST
Streptococcus pneumoniae	CSF or blood	PCR
Enteroviruses	CSF	PCR
Mycobacterium tuberculosis	CSF	PCR
Herpes simplex virus	CSF	PCR
Cryptococcus neoformans	CSF or serum	Antigen

CHILDREN	SPECIMEN	TEST
Streptococcus pneumoniae	CSF or blood	PCR
Haemophilus influenzae	CSF or blood	PCR
Mycobacterium tuberculosis	CSF	PCR
Neisseria meningitidis	CSF or blood	PCR
Herpes simplex virus	CSF	PCR

ADULTS WITH HIV INFECTION	SPECIMEN	TEST
Mycobacterium tuberculosis	CSF	PCR
Cryptococcus neoformans	CSF or serum	Antigen
Cytomegalovirus	CSF	PCR
Herpes simplex virus	CSF	PCR
Varicella zoster virus	CSF	PCR
Streptococcus pneumoniae	CSF or blood	PCR

STEP 1B TESTS

ADULTS WITHOUT HIV INFECTION	SPECIMEN	TEST
Haemophilus influenzae	CSF or serum	PCR
Neisseria meningitidis	CSF or serum	PCR
Zika virus	CSF or serum	PCR
Dengue virus	CSF or serum	PCR
Chikungunya virus	CSF or serum	PCR
West Nile virus	CSF or serum	PCR
Varicella zoster virus	CSF	PCR
Leptospira species	CSF or serum	PCR

CHILDREN	SPECIMEN	TEST
Enteroviruses	CSF	PCR
Zika virus	CSF or blood	PCR
Dengue virus	CSF or blood	PCR
Chikungunya virus	CSF or blood	PCR
West Nile virus	CSF or blood	PCR
Cryptococcus neoformans	CSF or serum	Antigen
Leptospira	CSF or serum	PCR

ADULTS WITH HIV INFECTION	SPECIMEN	TEST
Haemophilus influenzae	CSF or blood	PCR
Neisseria meningitidis	CSF or blood	PCR
Enteroviruses	CSF	PCR
Zika virus	CSF or blood	PCR
Dengue virus	CSF or blood	PCR
Chikungunya virus	CSF or blood	PCR
West Nile virus	CSF or blood	PCR
Toxoplasma gondii	CSF	PCR
Leptospira species	CSF or serum	PCR

STEP 2 TESTS

ADULTS WITHOUT HIV INFECTION	SPECIMEN	TEST
Rabies virus	CSF	PCR
Taenia solium	Serum	Serology
Syphilis	CSF and serum	Serology

CHILDREN	SPECIMEN	TEST
Parechovirus	CSF	PCR
Adenovirus	CSF	PCR
CMV	CSF	PCR
Syphilis	CSF and serum	Serology
Toxoplasma	CSF	PCR
Measles	CSF and serum	Serology
Rabies	CSF	PCR
Varicella zoster virus	CSF	PCR

ADULTS WITH HIV INFECTION	SPECIMEN	TEST
Measles	CSF and serum	Serology
Parvovirus (genus)	CSF	PCR
JC	CSF	PCR
Rabies	CSF	PCR
Taenia solium	Serum	Serology
Syphilis	CSF and serum	Serology

Tests for the following pathogens could not be implemented, for operational reasons: *Leptospira* species, Rabies virus, *Taenia solium*, Parechovirus, Adenovirus, *Toxoplasma*, Measles virus, and JC virus.
Validated in-house primers and probes were used for all PCR tests, except that commercial kits were used for *Mycobacterium tuberculosis*, dengue, chikungunya and Zika PCRs. A commercial lateral flow assay was used for cryptococcal antigen testing.

Figure S2.6: Diagnostic testing panel used in the intervention: India

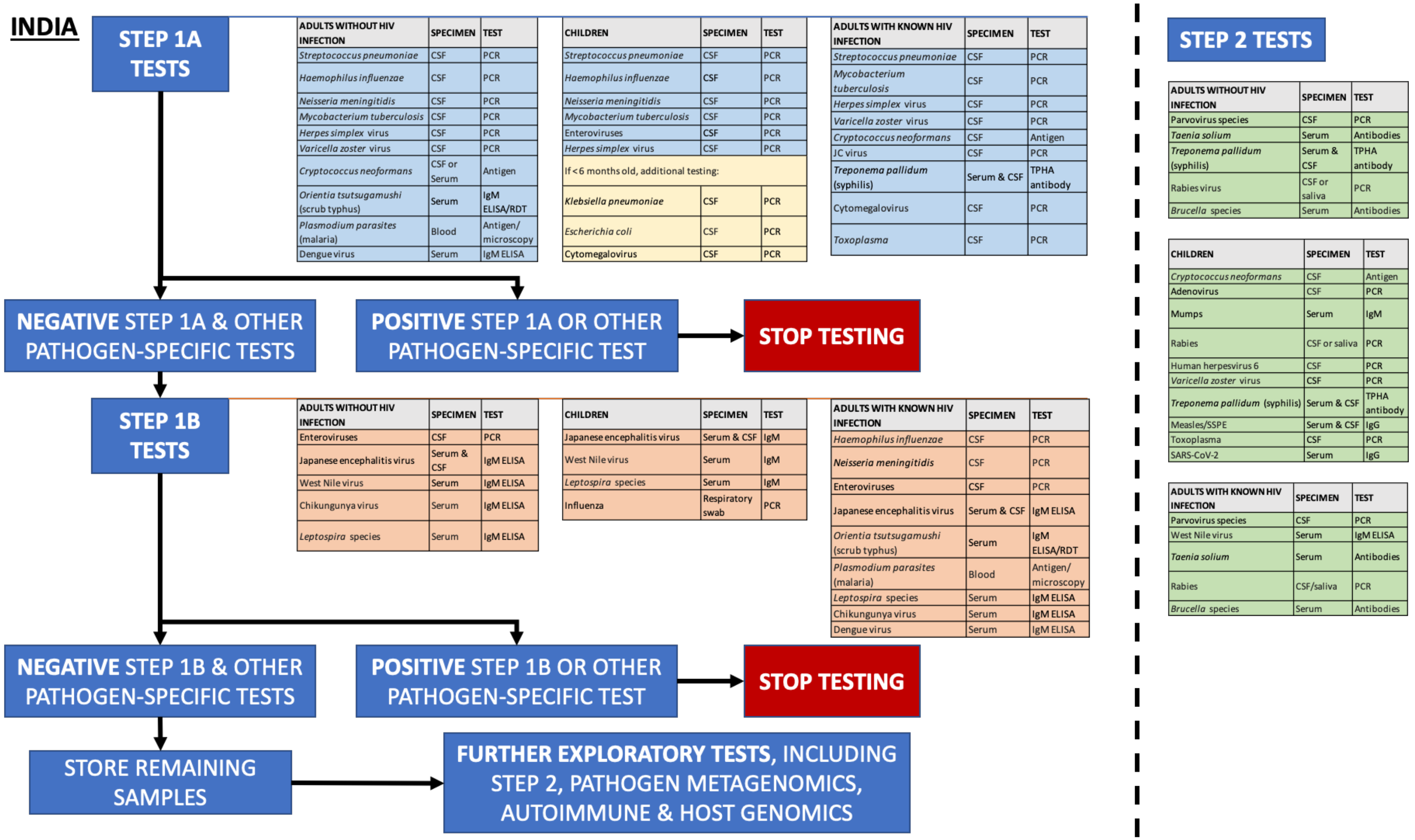

STEP 1A TESTS

ADULTS WITHOUT HIV INFECTION	SPECIMEN	TEST
Streptococcus pneumoniae	CSF	PCR
Haemophilus influenzae	CSF	PCR
Neisseria meningitidis	CSF	PCR
Mycobacterium tuberculosis	CSF	PCR
Herpes simplex virus	CSF	PCR
Varicella zoster virus	CSF	PCR
Cryptococcus neoformans	CSF or Serum	Antigen
Orientia tsutsugamushi (scrub typhus)	Serum	IgM ELISA/RDT
Plasmodium parasites (malaria)	Blood	Antigen/ microscopy
Dengue virus	Serum	IgM ELISA

CHILDREN	SPECIMEN	TEST
Streptococcus pneumoniae	CSF	PCR
Haemophilus influenzae	CSF	PCR
Neisseria meningitidis	CSF	PCR
Mycobacterium tuberculosis	CSF	PCR
Enteroviruses	CSF	PCR
Herpes simplex virus	CSF	PCR
If < 6 months old, additional testing:		
Klebsiella pneumoniae	CSF	PCR
Escherichia coli	CSF	PCR
Cytomegalovirus	CSF	PCR

ADULTS WITH KNOWN HIV INFECTION	SPECIMEN	TEST
Streptococcus pneumoniae	CSF	PCR
Mycobacterium tuberculosis	CSF	PCR
Herpes simplex virus	CSF	PCR
Varicella zoster virus	CSF	PCR
Cryptococcus neoformans	CSF	Antigen
JC virus	CSF	PCR
Treponema pallidum (syphilis)	Serum & CSF	TPHA antibody
Cytomegalovirus	CSF	PCR
Toxoplasma	CSF	PCR

STEP 1B TESTS

ADULTS WITHOUT HIV INFECTION	SPECIMEN	TEST
Enteroviruses	CSF	PCR
Japanese encephalitis virus	Serum & CSF	IgM ELISA
West Nile virus	Serum	IgM ELISA
Chikungunya virus	Serum	IgM ELISA
Leptospira species	Serum	IgM ELISA

CHILDREN	SPECIMEN	TEST
Japanese encephalitis virus	Serum & CSF	IgM
West Nile virus	Serum	IgM
Leptospira species	Serum	IgM
Influenza	Respiratory swab	PCR

ADULTS WITH KNOWN HIV INFECTION	SPECIMEN	TEST
Haemophilus influenzae	CSF	PCR
Neisseria meningitidis	CSF	PCR
Enteroviruses	CSF	PCR
Japanese encephalitis virus	Serum & CSF	IgM ELISA
Orientia tsutsugamushi (scrub typhus)	Serum	IgM ELISA/RDT
Plasmodium parasites (malaria)	Blood	Antigen/ microscopy
Leptospira species	Serum	IgM ELISA
Chikungunya virus	Serum	IgM ELISA
Dengue virus	Serum	IgM ELISA

STEP 2 TESTS

ADULTS WITHOUT HIV INFECTION	SPECIMEN	TEST
Parvovirus species	CSF	PCR
Taenia solium	Serum	Antibodies
Treponema pallidum (syphilis)	Serum & CSF	TPHA antibody
Rabies virus	CSF or saliva	PCR
Brucella species	Serum	Antibodies

CHILDREN	SPECIMEN	TEST
Cryptococcus neoformans	CSF	Antigen
Adenovirus	CSF	PCR
Mumps	Serum	IgM
Rabies	CSF or saliva	PCR
Human herpesvirus 6	CSF	PCR
Varicella zoster virus	CSF	PCR
Treponema pallidum (syphilis)	Serum & CSF	TPHA antibody
Measles/SSPE	Serum & CSF	IgG
Toxoplasma	CSF	PCR
SARS-CoV-2	Serum	IgG

ADULTS WITH KNOWN HIV INFECTION	SPECIMEN	TEST
Parvovirus species	CSF	PCR
West Nile virus	Serum	IgM ELISA
Taenia solium	Serum	Antibodies
Rabies	CSF/saliva	PCR
Brucella species	Serum	Antibodies

Tests for the following pathogens could not be implemented, for operational reasons: *Klebsiella pneumoniae*, *Escherichia coli*, and Influenza virus.
Validated in-house primers and probes were used for all PCR tests, except that commercial kits were used for *Mycobacterium tuberculosis* in both centres and for Varicella zoster virus in Bangalore. Commercial assays were used for all antigen/antibody tests, except for the in-house measles serology.

Figure S2.7: Diagnostic testing panel used in the intervention: Malawi

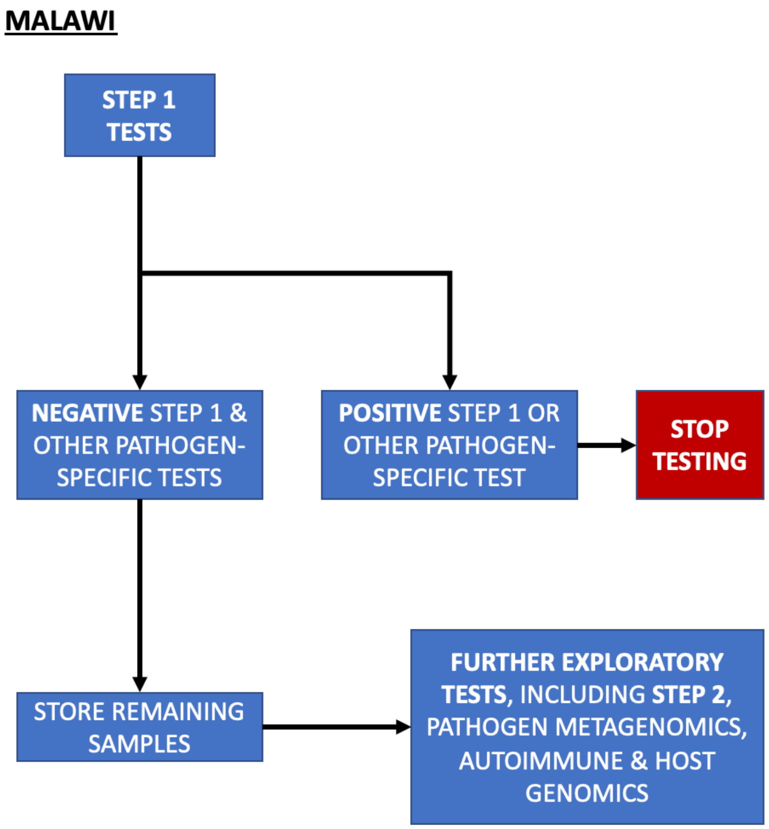

CENTRAL HOSPITAL

STEP	ADULTS AT CENTRAL HOSPITAL	SPECIMEN	TEST
1	*Mycobacterium tuberculosis*	CSF	PCR
	Cryptococcus neoformans	CSF or serum	Antigen
	Plasmodium falciparum	Blood	Antigen
	Streptococcus pneumoniae	CSF	PCR
	Enteroviruses	CSF	PCR
	Herpes simplex virus	CSF	PCR
	Haemophilus influenzae	CSF	PCR
	Neisseria meningitidis	CSF	PCR
	Salmonellae	CSF	PCR
	Listeria monocytogenes	CSF	PCR
2	*Varicella zoster* virus	CSF	PCR
	Toxoplasma gondii	CSF or serum	PCR
	Cytomegalovirus	CSF	PCR
	Parechovirus	CSF	PCR
	Escherichia coli	CSF	PCR
	Klebsiella species	CSF	PCR
	Streptococcus agalactiae (Group B *Strep.*)	CSF	PCR
	Rabies virus	CSF or blood	PCR

STEP	CHILDREN AT CENTRAL HOSPITAL	SPECIMEN	TEST
1	*Mycobacterium tuberculosis*	CSF	PCR
	Cryptococcus neoformans	CSF or serum	Antigen
	Plasmodium falciparum	Blood	Antigen
	Streptococcus pneumoniae	CSF	PCR
	Enteroviruses	CSF	PCR
	Herpes simplex virus	CSF	PCR
	Haemophilus influenzae	CSF	PCR
	Neisseria meningitidis	CSF	PCR
	Salmonellae	CSF	PCR
	Listeria monocytogenes	CSF	PCR
	Staphylococcus aureus	CSF	PCR
	Enterococcus	CSF	PCR
	Adenovirus	CSF	PCR
	Cytomegalovirus	CSF	PCR
	Parechovirus	CSF	PCR
	Escherichia coli	CSF	PCR
	Klebsiella species	CSF	PCR
	Streptococcus agalactiae (Group B Strep.)	CSF	PCR
	Varicella zoster virus	CSF	PCR
2	Rubella	CSF	PCR
	Streptococcus pyogenes (Group A *Strep*)	CSF	PCR
	Pseudomonas	CSF	PCR
	Rabies virus	CSF or blood	PCR

DISTRICT HOSPITALS

STEP	ADULTS AT DISTRICT HOSPITALS	SPECIMEN	TEST
1	*Mycobacterium tuberculosis*	CSF	PCR
	Cryptococcus neoformans	CSF or serum	Antigen
	Plasmodium falciparum	Blood	Antigen
2	*Streptococcus pneumoniae*	CSF	PCR
	Enteroviruses	CSF	PCR
	Herpes simplex virus	CSF	PCR
	Haemophilus influenzae	CSF	PCR
	Neisseria meningitidis	CSF	PCR
	Salmonellae	CSF	PCR
	Listeria monocytogenes	CSF	PCR
	Varicella zoster virus	CSF	PCR
	Toxoplasma gondii	CSF or serum	PCR
	Cytomegalovirus	CSF	PCR
	Parechovirus	CSF	PCR
	Escherichia coli	CSF	PCR
	Klebsiella species	CSF	PCR
	Streptococcus agalactiae (Group B *Strep.*)	CSF	PCR
	Rabies virus	CSF or blood	PCR

STEP	CHILDREN AT DISTRICT HOSPITALS	SPECIMEN	TEST
1	*Mycobacterium tuberculosis*	CSF	PCR
	Cryptococcus neoformans	CSF or serum	Antigen
	Plasmodium falciparum	Blood	Antigen
2	*Streptococcus pneumoniae*	CSF	PCR
	Enteroviruses	CSF	PCR
	Herpes simplex virus	CSF	PCR
	Haemophilus influenzae	CSF	PCR
	Neisseria meningitidis	CSF	PCR
	Salmonellae	CSF	PCR
	Listeria monocytogenes	CSF	PCR
	Staphylococcus aureus	CSF	PCR
	Enterococcus	CSF	PCR
	Adenovirus	CSF	PCR
	Cytomegalovirus	CSF	PCR
	Parechovirus	CSF	PCR
	Escherichia coli	CSF	PCR
	Klebsiella species	CSF	PCR
	Streptococcus agalactiae (Group B Strep.)	CSF	PCR
	Varicella zoster virus	CSF	PCR
	Rubella	CSF	PCR
	Streptococcus pyogenes (Group A Strep)	CSF	PCR
	Pseudomonas	CSF	PCR
	Rabies virus	CSF or blood	PCR

Tests for the following pathogens could not be implemented, for operational reasons: Rubella virus, *Streptococcus pyogenes* (group A *Streptococcus*), *Pseudomonas* and Rabies virus.
Validated in-house primers and probes were used for all PCR tests, except that commercial kits were used for *Mycobacterium tuberculosis*. Commercial assays were used for the antigen tests.

Figure S2.8: Achievement of syndromic diagnosis by month, in each centre

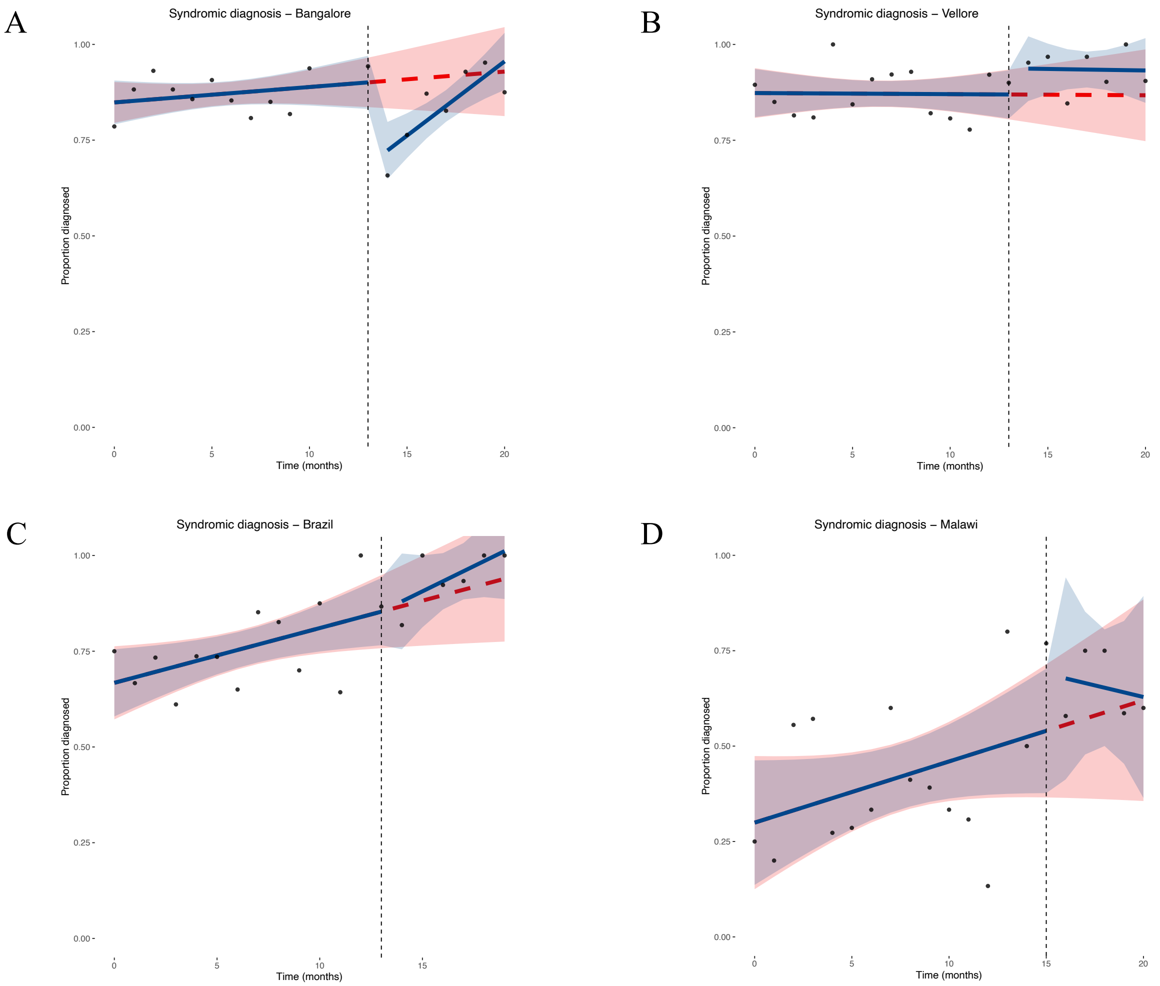

(A) Bangalore (B) Vellore (C) Brazil (D) Malawi.Dots represent proportions of patients achieving a diagnosis in each month of recruitment. The blue line represents the observed trend across these points. The blue ribbon around this line represents a 95% confidence interval around these proportions. The dashed red line represents the counterfactual situation: a predicted trend assuming no intervention was delivered, based on pre-intervention data. The red ribbon around this line represents a 95% confidence interval around these proportions. The vertical dashed black line represents the time point at which the intervention began to be implemented.

Figure S2.9: Pathogens identified as confirmed or probable causes of suspected acute brain infection

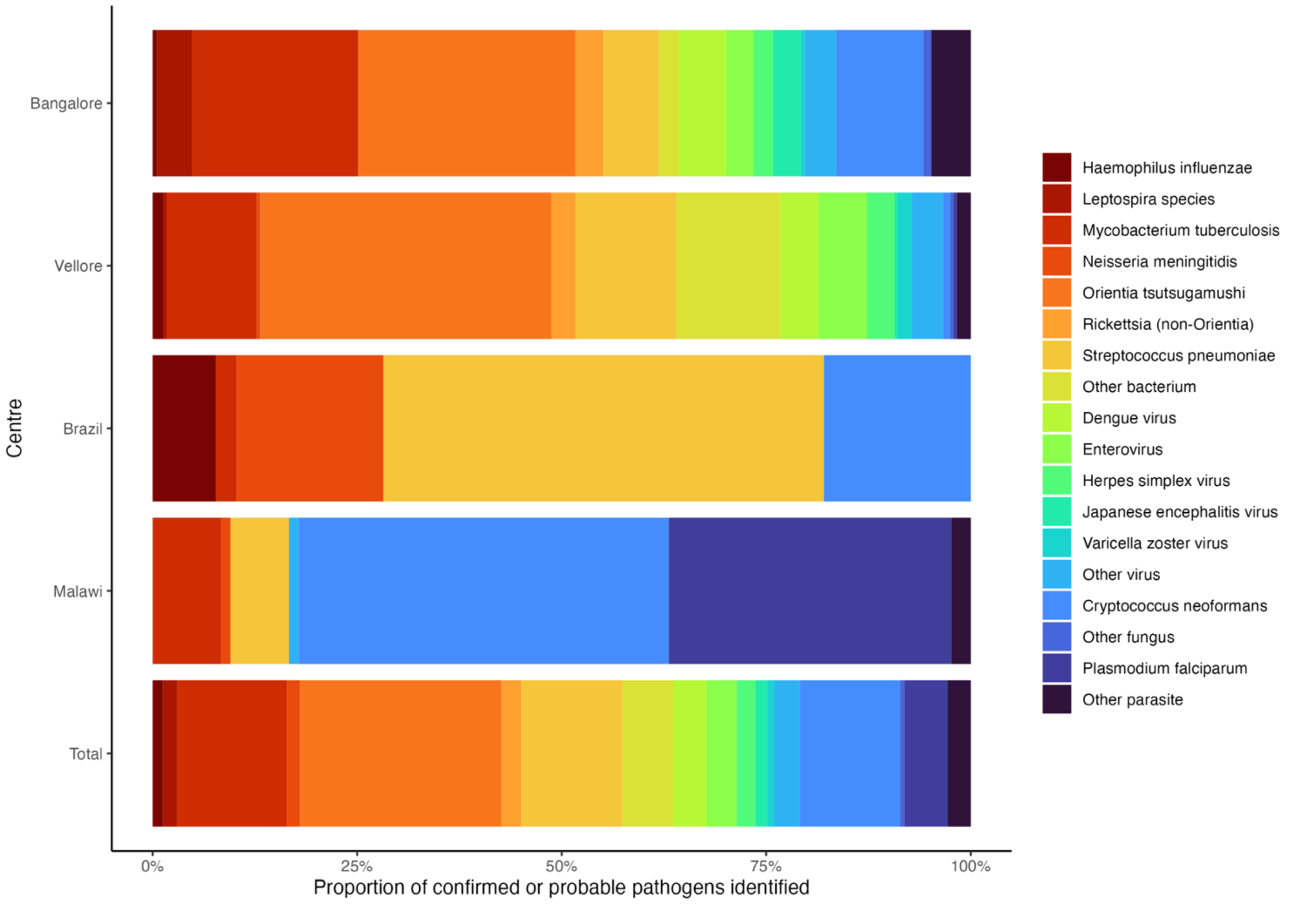

Figure S2.10: Achievement of microbiological diagnosis by month

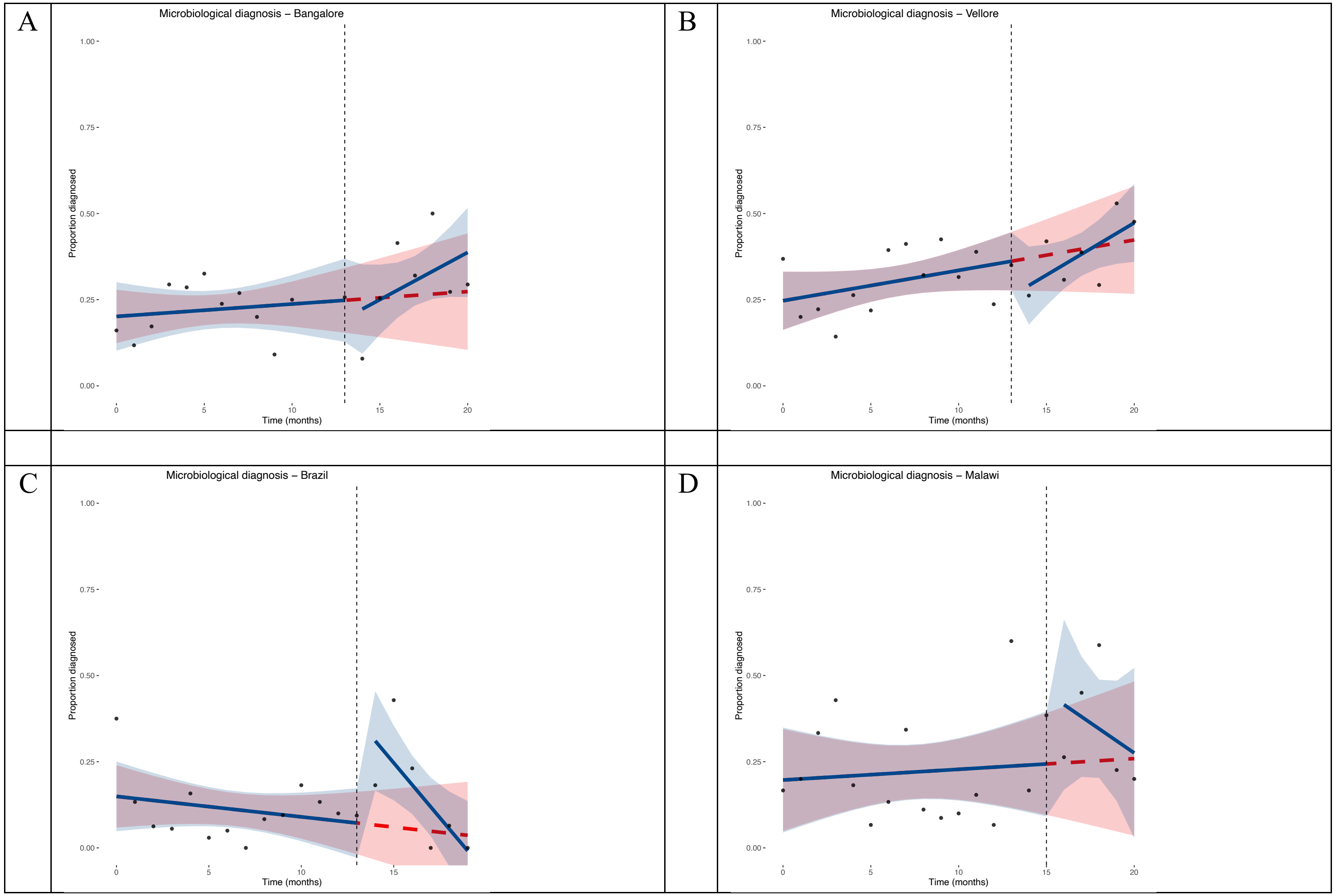

(A) Bangalore. (B) Vellore. (C) Brazil. (D) Malawi. Dots represent proportions of patients achieving a diagnosis in each month of recruitment. The blue line represents the observed trend across these points. The blue ribbon around this line represents a 95% confidence interval around these proportions. The dashed red line represents the counterfactual situation: a predicted trend assuming no intervention was delivered, based on pre-intervention data. The red ribbon around this line represents a 95% confidence interval around these proportions. The vertical dashed black line represents the time point at which the intervention began to be implemented.

Figure S2.11: Time to lumbar puncture, overall and in each centre

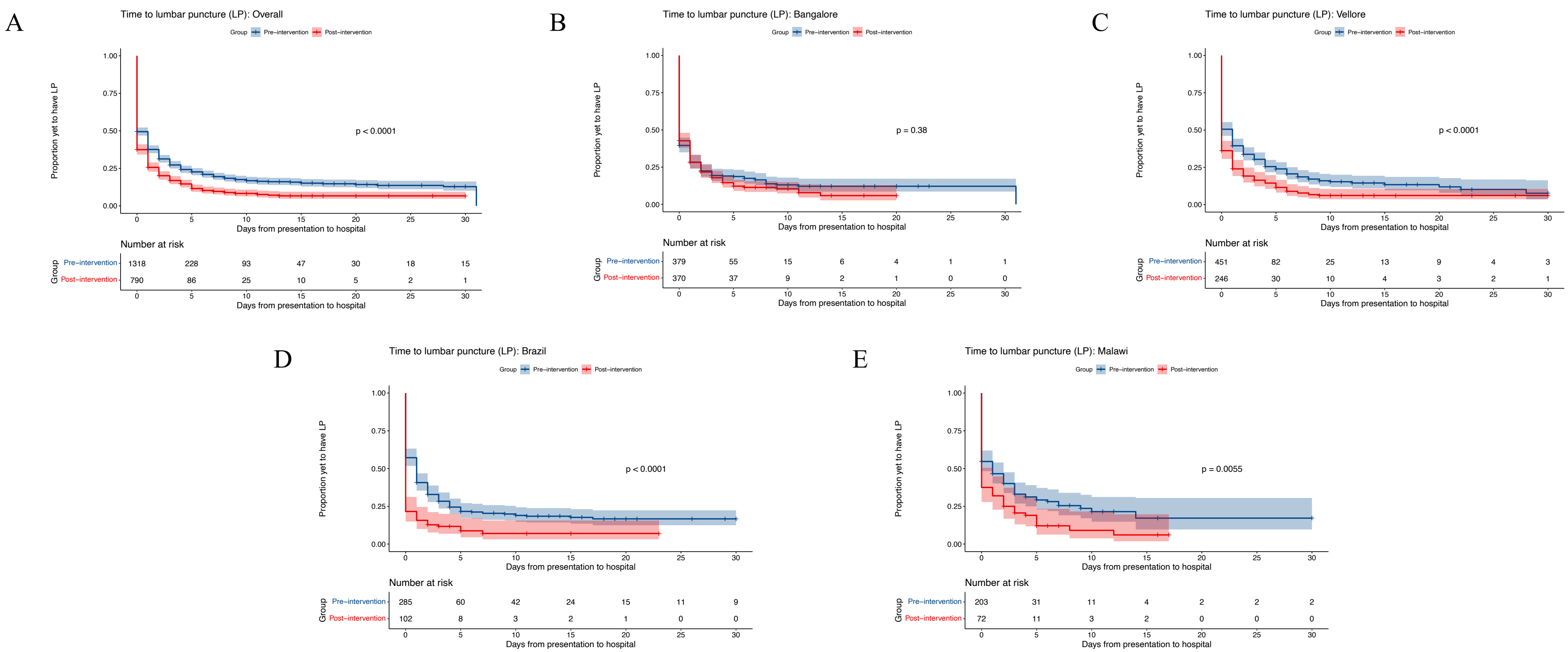

(A) Overall dataset (B) Bangalore centre (C) Vellore centre (D) Brazil centre (E) Malawi centre. The ribbons around the lines represent 95% confidence intervals.

Figure S2.12: Time to appropriate empirical therapy, overall and in each centre

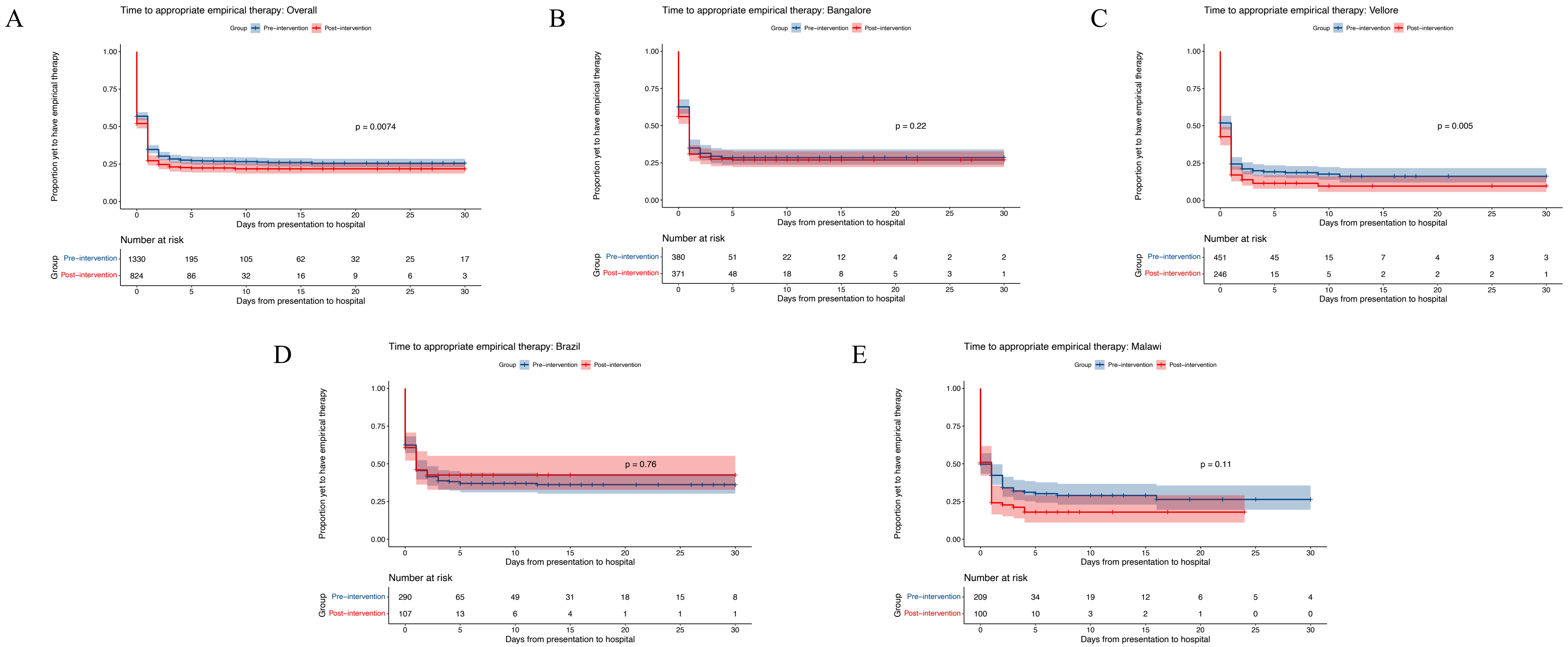

(A) Overall dataset (B) Bangalore centre (C) Vellore centre (D) Brazil centre (E) Malawi centre. The ribbons around the lines represent 95% confidence intervals.

Figure S2.13: Liverpool Outcome Score at discharge from hospital and at 30-day follow-up, overall

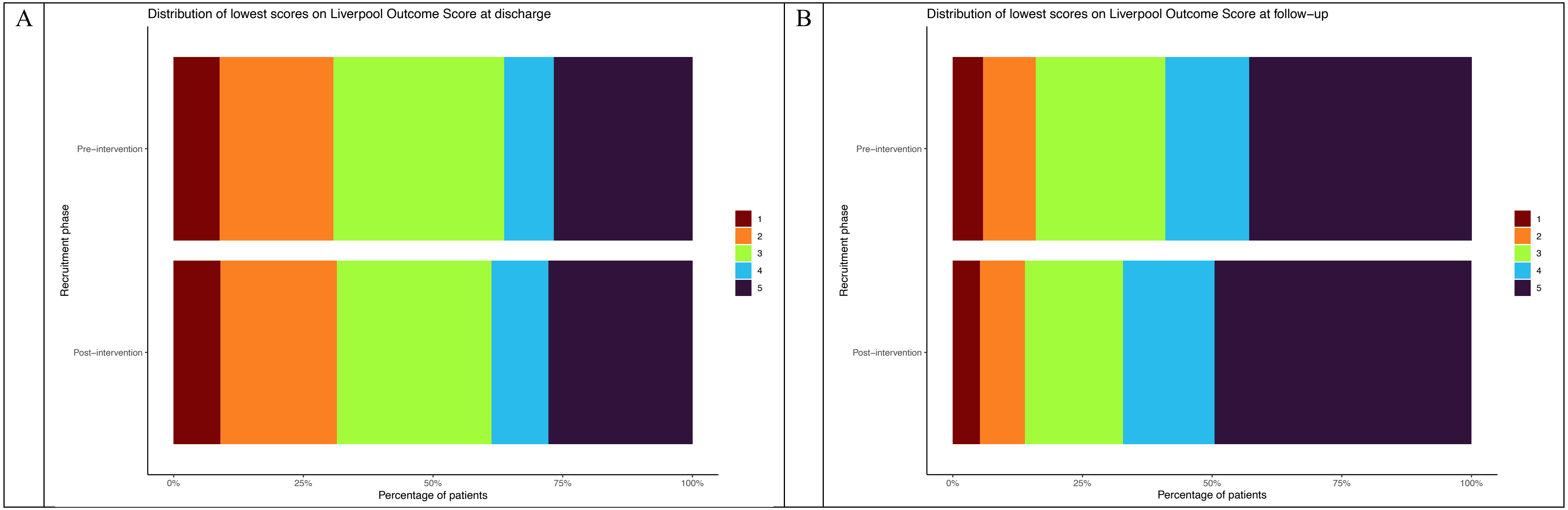

The final Liverpool Outcome Score is the lowest number scored for any single question, and represents the following functional outcome states: 5 = full recovery & normal neurological examination; 4 = minor sequelae with mild effects on function or personality change or on medication; 3 = moderate sequelae mildly affecting function, probably compatible with independent living; 2 = severe sequelae, impairing function sufficient to make patient dependent; 1 = death.
Table 3 (main paper) and Table S2.3 (appendix 2 p 19) provide numerical results for Liverpool Outcome Score for overall data and for each centre, respectively.

Table S2.1: Confirmed and probable pathogens diagnosed in each centre and overall

**Pathogen**	**Bangalore**	**Vellore**	**Brazil**	**Malawi**	**Overall**
Haemophilus influenzae	1 (0%)	3 (1%)	3 (8%)	0 (0%)	7 (1%)
Leptospira species	9 (4%)	1 (0%)	0 (0%)	0 (0%)	10 (2%)
Mycobacterium tuberculosis	42 (20%)	26 (11%)	1 (3%)	7 (8%)	76 (13%)
Neisseria meningitidis	0 (0%)	1 (0%)	7 (18%)	1 (1%)	9 (2%)
Orientia tsutsugamushi	55 (27%)	84 (36%)	0 (0%)	0 (0%)	139 (25%)
Rickettsia (non-Orientia)	7 (3%)	7 (3%)	0 (0%)	0 (0%)	14 (2%)
Streptococcus pneumoniae	14 (7%)	29 (12%)	21 (54%)	6 (7%)	70 (12%)
Other bacterium	5 (2%)	30 (13%)	0 (0%)	0 (0%)	35 (6%)
Dengue virus	12 (6%)	11 (5%)	0 (0%)	0 (0%)	23 (4%)
Enterovirus	7 (3%)	14 (6%)	0 (0%)	0 (0%)	21 (4%)
Herpes simplex virus	5 (2%)	8 (3%)	0 (0%)	0 (0%)	13 (2%)
Japanese encephalitis virus	7 (3%)	1 (0%)	0 (0%)	0 (0%)	8 (1%)
Varicella zoster virus	1 (0%)	4 (2%)	0 (0%)	0 (0%)	5 (1%)
Other virus	8 (4%)	9 (4%)	0 (0%)	1 (1%)	18 (3%)
Cryptococcus neoformans	22 (11%)	2 (1%)	7 (18%)	38 (45%)	69 (12%)
Other fungus	2 (1%)	1 (0%)	0 (0%)	0 (0%)	3 (1%)
Plasmodium falciparum	0 (0%)	1 (0%)	0 (0%)	29 (35%)	30 (5%)
Other parasite	10 (5%)	4 (2%)	0 (0%)	2 (2%)	16 (3%)
Total with a confirmed or probable pathogen	207	236	39	84	566

Table S2.2: Confirmed and probable pathogens diagnosed pre- and post-intervention

	**Pre-intervention (n=1330)**	**Post-intervention (n=824)**
**Confirmed or probable pathogens**	294 (22.1%)	250 (30.3%)
**Confirmed**	137 (10.3%)	130 (15.8%)
**Probable**	161 (12.1%)	126 (15.3%)
**Confirmed or probable bacterial infection**	189 (14.2%)	160 (19.4%)
Streptococcus pneumoniae	32 (2.4%)	38 (4.6%)
Orientia tsutsugamushi	82 (6.2%)	57 (6.9%)
Mycobacterium tuberculosis	42 (3.2%)	34 (4.1%)
Leptospira	3 (0.2%)	7 (0.8%)
Rickettsia (non-Orientia)	11 (0.8%)	3 (0.4%)
**Confirmed or probable viral infection**	35 (2.6%)	52 (6.3%)
**Herpes simplex virus**	11 (0.8%)	2 (0.2%)
**Dengue virus**	12 (0.9%)	11 (1.3%)
**Confirmed or probable fungal infection**	42 (3.2%)	29 (3.5%)
Cryptococcus neoformans	40 (3%)	29 (3.5%)
**Confirmed or probable parasitic infection**	28 (2.1%)	16 (1.9%)
Plasmodium falciparum	19 (1.4%)	11 (1.3%)

Table S2.3: Secondary outcome results, by centre

	Bangalore		Vellore		Brazil		Malawi	
	Pre-intervention (n=380)	Post-intervention (n=371)	Pre-intervention (n=451)	Post-intervention (n=246)	Pre-intervention (n=290)	Post-intervention (n=107)	Pre-intervention (n=209)	Post-intervention (n=100)
**Lumbar puncture performed**								
n (%)	315 (83%)	320 (86%)	360 (80%)	223 (91%)	234 (81%)	98 (92%)	146 (70%)	92 (92%)
Univariate OR (95% CI) vs pre-intervention	··	1·29 (0·87–1·93)	··	2·45 (1·53–4·07)	··	2·61 (1·30–5·83)	··	4·96 (2·40–11·65)
Univariate p value	··	0·20	··	0·00031	··	0·011	··	<0·0001
aOR (95% CI) vs pre-intervention	··	1·72 (1·02–2·88)	··	2·23 (1·34–3·70)	··	0·88 (0·36–2·17)	··	2·19 (0·81–5·94)
Adjusted p value	··	0·042	··	0·0019	··	0·78	··	0·12
Step change vs pre-intervention*	··	0·10	··	0·026	··	··	··	··
Slope change vs pre-intervention*	··	0·61	··	0·063	··	··	··	··
**Time to lumbar puncture, hours**								
Median (IQR)	9 (4–26)	14 (4–31·5)	15 (5–44)	9 (4–28)	21 (1–50)	1 (0–4·5)	16 (1–42·5)	10 (1–29·5)
Univariate HR (95% CI) vs pre-intervention	··	1·04 (0·89–1·22)	··	1·46 (1·23–1·72)	··	1·95 (1·53–2·49)	··	1·54 (1·14–2·07)
Univariate p value	··	0·61	··	<0·0001	··	<0·0001	··	0·0046
aHR (95% CI) vs pre-intervention	··	1·15 (0·98–1·35)	··	1·46 (1·23–1·74)	··	0·87 (0·65–1·17)	··	1·25 (0·89–1·75)
Adjusted p value	··	0·088	··	<0·0001	··	0·37	··	0·20
**Basic CSF tests performed – all†**								
n/N (%)	190/312 (61%)	151/313 (48%)	258/359 (72%)	173/221 (78%)	0/232	0/97	0/145	0/92
Univariate OR (95% CI) vs pre-intervention	··	0·60 (0·44–0·82)	··	1·41 (0·96–2·10)	··	··	··	··
Univariate p value	··	0·002	··	0·087	··	··	··	··
aOR (95% CI) vs pre-intervention	··	0·70 (0·48–1·01)	··	1·40 (0·90–2·16)	··	··	··	··
Adjusted p value	··	0·055	··	0·13	··	··	··	··
**Basic CSF tests performed – excluding paired blood glucose†**								
n/N (%)	247/312 (79%)	245/320 (77%)	282/359 (79%)	178/221 (81%)	47/232 (20%)	28/95 (30%)	5/145 (3%)	20/92 (22%)
Univariate OR (95% CI) vs pre-intervention	··	0·86 (0·59–1·25)	··	1·13 (0·75–1·73)	··	1·64 (0·95–2·83)	··	··
Univariate p value	··	0·43	··	0·57	··	0·073	··	··

	Bangalore		Vellore		Brazil		Malawi	
	**Pre-intervention (n=380)**	**Post-intervention (n=371)**	**Pre-intervention (n=451)**	**Post-intervention (n=246)**	**Pre-intervention (n=290)**	**Post-intervention (n=107)**	**Pre-intervention (n=209)**	**Post-intervention (n=100)**
aOR (95% CI) vs pre-intervention	··	0·93 (0·62–1·40)	··	0·99 (0·63–1·55)	··	0·67 (0·35–1·28)	··	··
Adjusted p value	··	0·73	··	0·96	··	0·23	··	··
**Time to appropriate empirical therapy, days**								
Median (IQR)	0 (0–1)	0 (0–1)	0 (0–1)	0 (0–1)	0 (0–1)	0 (0–0)	0 (0–1)	0 (0–1)
Received on day of or day after presentation	282/380 (74%)	296/371 (80%)	362/451 (80%)	214/246 (87%)	201/290 (69%)	80/107 (75%)	132/209 (63%)	83/100 (83%)
Univariate HR (95% CI) vs pre-intervention	··	1·12 (0·94–1·34)	··	1·27 (1·07–1·51)	··	0·97 (0·71–1·32)	··	1·22 (0·92–1·63)
Univariate p value	··	0·20	··	0·0063	··	0·82	··	0·17
aHR (95% CI) vs pre-intervention	··	1·01 (0·82–1·25)	··	1·26 (1·05–1·51)	··	0·60 (0·41–0·87)	··	1·03 (0·70–1·51)
Adjusted p value	··	0·92	··	0·012	··	0·0066	··	0·87
**Time to appropriate definitive therapy, days**								
Median (IQR)	1 (0–2)	1 (0–1)	0 (0–1)	0 (0–1)	0 (0–1)	0 (0–1)	0 (0–2)	0 (0–3)
Univariate HR (95% CI) vs pre-intervention	··	0·83 (0·59–1·16)	··	1·07 (0·79–1·45)	··	1·74 (0·83–3·64)	··	0·91 (0·55–1·52)
Univariate p value	··	0·27	··	0·64	··	0·14	··	0·73
aHR (95% CI) vs pre-intervention	··	0·67 (0·45–1·01)	··	1·21 (0·88–1·66)	··	1·48 (0·60–3·68)	··	1·06 (0·50–2·24)
Adjusted p value	··	0·058	··	0·25	··	0·40	··	0·89
**Time to discharge from hospital, days**	n=230							
Median (IQR)	7 (4–11)	7 (3–11)	6 (4–10)	7 (3–12)	13·5 (8–23)	8 (4–13·75)	6 (3–12)	7 (4–15)
Univariate HR (95% CI) vs pre-intervention	··	0·96 (0·81–1·15)	··	0·88 (0·74–1·04)	··	2.00 (1·57–2·54)	··	0·92 (0·71–1·21)
Univariate p value	··	0·67	··	0·13	··	<0·001	··	0·56
aHR (95% CI) vs pre-intervention	··	1.04 (0·88–1·22)	··	0·96 (0·81–1·12)	··	1·54 (1·18–2·01)	··	1·06 (0·81–1·37)
Adjusted p value	··	0·66	··	0·59	··	0·001	··	0·68
**EQ-5D score at discharge**	n=230	n=172	n=279	n=145	n=201	n=36	n=84	n=43
Mean (SD)	0·52 (0·47)	0·53 (0·49)	0·50 (0·51)	0·45 (0·49)	0·69 (0·39)	0·94 (0·17)	0·76 (0·34)	0·73 (0·30)
Mean difference (95% CI) vs pre-intervention	··	0·01 (–0·09–0·10)	··	–0·05 (–0·15–0·05)	··	0·25 (0·17–0·33)	··	–0·03 (–0·15–0·09)
p value	··	0·88	··	0·32	··	<0·0001	··	0·62

	Bangalore		Vellore		Brazil		Malawi	
	Pre-intervention (n=380)	Post-intervention (n=371)	Pre-intervention (n=451)	Post-intervention (n=246)	Pre-intervention (n=290)	Post-intervention (n=107)	Pre-intervention (n=209)	Post-intervention (n=100)
**EQ-5D score at follow-up**	n=201	n=130	n=261	n=137	n=180	n=31	n=26	n=34
Mean (SD)	0·74 (0·32)	0·73 (0·46)	0·77 (0·38)	0·77 (0·37)	0·75 (0·33)	0·95 (0·15)	0·93 (0·24)	0·90 (0·28)
Mean difference (95% CI) vs pre-intervention	··	+0·01 (–0·10–0·09)	··	0·00 (–0·07–0·08)	··	+0·20 (0·12–0·27)	··	–0·03 (–0·16–0·10)
p value	··	0·90	··	0·91	··	<0·0001	··	0·68
**Liverpool Outcome Score at discharge**	n=266	n=215	n=343	n=177	n=228	n=52	n=133	n=67
Median (IQR)	3 (2–3)	3 (2–4)	3 (2–4·5)	3 (2–4)	3·5 (3–5)	5 (5–5)	4 (2–5)	3 (2–5)
Good outcome (lowest score of 4 or 5)	48 (18%)	66 (31%)	117 (34%)	54 (31%)	114 (50%)	48 (92%)	73 (55%)	30 (45%)
aOR (95% CI) vs pre-intervention	··	1·53 (1·10–2·13)	··	0·83 (0·60–1·16)	··	4·73 (2·42–9·88)	··	0·81 (0·48–1·40)
Adjusted p value	··	0·012	··	0·28	··	<0·0001	··	0·46
**Liverpool Outcome Score at follow-up**	n=229	n=156	n=321	n=159	n=201	n=45	n=35	n=42
Median (IQR)	3 (3–5)	4 (3–5)	4 (3–5)	4 (3–5)	4 (3–5)	5 (4–5)	5 (3–5)	5 (3–5)
Good outcome (lowest score of 4 or 5)	100 (44%)	96 (62%)	213 (66%)	109 (69%)	127 (63%)	37 (82%)	24 (69%)	28 (67%)
aOR (95% CI) vs pre-intervention	··	1·61 (1·10–2·36)	··	1·07 (0·76–1·52)	··	2·20 (1·11–4·59)	··	0·95 (0·37–2·41)
Adjusted p value	··	0·014	··	0·71	··	0·028	··	0·91
**Mortality**								
n (%)	35 (9%)	31 (8%)	21 (5%)	14 (6%)	10 (3%)	1 (1%)	30 (14%)	12 (12%)
Univariate OR (95% CI) vs pre-intervention	··	0·88 (0·53–1·45)	··	1·28 (0·63–2·55)	··	0·26 (0·01–1·40)	··	0·81 (0·38–1·63)
Univariate p value	··	0·61	··	0·49	··	0·21	··	0·57
aOR (95% CI) vs pre-intervention	··	0·86 (0·51–1·46)	··	1·29 (0·62–2·65)	··	0·94 (0·11–8·09)	··	0·84 (0·40–1·77)
Adjusted p value	··	0·57	··	0·50	··	0·96	··	0·65

aHR = adjusted hazard ratio; aOR = adjusted odds ratio; CSF=cerebrospinal fluid; HR = hazard ratio; OR = odds ratio. *From interrupted time series analysis, performed only for outcomes measured as proportions, for which a pre- versus post-intervention comparison yielded a significant improvement. †Includes only patients who had a lumbar puncture.

Table S2.4: Process measures for pathogen testing

	Pre-intervention	Post-intervention	p value
**Any serological test**			
Bangalore	282 (74%)	353 (95%)	<0·0001
Vellore	274 (61%)	231 (94%)	<0·0001
Brazil	28 (10%)	16 (15%)	0·19
Malawi	130 (62%)	77 (77%)	0·014
Overall	714 (54%)	677 (82%)	<0·0001
**Any PCR test**			
Bangalore	159 (42%)	263 (71%)	<0·0001
Vellore	213 (47%)	220 (89%)	<0·0001
Brazil	54 (19%)	65 (61%)	<0·0001
Malawi	7 (3%)	68 (68%)	<0·0001
Overall	433 (33%)	616 (75%)	<0·0001
**Priority pathogens as defined in diagnostic panel***			
Bangalore	23 (6%)	236 (64%)	<0·0001
Vellore	72 (16%)	214 (87%)	<0·0001
Brazil	0	12 (11%)	<0·0001
Malawi	2 (1%)	37 (37%)	<0·0001
Overall	97 (7%)	499 (61%)	<0·0001

*For the Indian centres, this was testing for any five pathogens from the first step of the diagnostic panel

Table S2.5: Cost of the intervention in each centre

	Bangalore, India	Vellore, India	Brazil	Malawi
Total cost of the intervention	$41 713	$71 664	$32 360	$25 684
Patients in post-intervention phase	371	246	107	100
Cost of the intervention per patient	$112	$291	$302	$257

Table S2.6: Syndromic diagnoses achieved

	Pre-intervention	Post-intervention	Total
**Outcome of syndromic diagnosis assessments**	**n=1330**	**n=824**	**n=2154**
Achieved - brain infection syndrome	825 (62%)	603 (73%)	1428 (66%)
Achieved - non-brain infection syndrome	195 (15%)	98 (12%)	293 (14%)
Syndromic diagnosis not achieved	300 (23%)	112 (14%)	412 (19%)
Unclassified	10 (1%)	11 (1%)	21 (1%)
**Brain infection diagnoses achieved**	**n=825**	**n=603**	**n=1428**
Encephalitis	324 (39%)	237 (39%)	561 (39%)
Meningitis	86 (10%)	73 (12%)	159 (11%)
Meningoencephalitis	376 (46%)	253 (42%)	629 (44%)
Other brain infection	39 (5%)	40 (7%)	79 (6%)

Table S2.7: Sensitivity, subgroup and post hoc analyses for the primary outcomes in the overall dataset

	Pre-intervention, n/N (%)	Post-intervention, n/N (%)	Multilevel analysis pre- versus post-intervention	
			Adjusted odds ratio (95% CI)	p value*
**Syndromic diagnosis achieved**				
Adjustment for hospital site†	··	··	1·65 (1·24–2·18)	0·00049
Adjustment for HIV infection†	··	··	1·73 (1·33–2·25)	<0·0001
Subgroup analysis: adults	610/802 (76%)	387/451 (86%)	1·63 (1·16–2·31)	0·0055
Subgroup analysis: children	410/518 (79%)	314/362 (87%)	2·00 (1·35–2·97)	0·00055
**Microbiological diagnosis achieved**				
Adjustment for hospital site†	··	··	1·33 (1·07–1·65)	0·0089
Adjustment for HIV infection†	··	··	1·42 (1·15–1·76)	0.0011
Subgroup analysis: adults	190/800 (24%)	150/457 (33%)	1·36 (1·03–1·78)	0·028
Subgroup analysis: children	102/518 (20%)	100/367 (27%)	1·58 (1·14–2·20)	0·0063
Including possible microbiological diagnoses	517/1330 (39%)	417/824 (51%)	1·46 (1·21–1·77)	<0·0001

*All p values represented a statistically significant change at a threshold of 0.05. †The sensitivity analyses adjusting for hospital site and for HIV infection were performed by adding them as covariates in the models used for the primary analyses, so numerators, denominators and percentages are as shown in table 2 of the main manuscript.
